# Unlocking the Activity of Molecular Assemblies for CO_2_ Electroreduction in Zero‐Gap Electrolysers via Catalyst Ink Engineering

**DOI:** 10.1002/smll.202408154

**Published:** 2024-10-31

**Authors:** Kevinjeorjios Pellumbi, Mena‐Alexander Kräenbring, Dominik Krisch, Wiebke Wiesner, Sebastian Sanden, Daniel Siegmund, Fatih Özcan, Kai junge Puring, Rui Cao, Wolfgang Schöfberger, Doris Segets, Ulf‐Peter Apfel

**Affiliations:** ^1^ Fraunhofer Institute for Environmental Safety and Energy Technology UMSICHT Osterfelderstraße 3 46047 Oberhausen Germany; ^2^ Institute for Energy and Materials Processes – Particle Science and Technology (EMPI‐PST) University of Duisburg‐Essen Carl‐Benz‐Straße 199 47057 Duisburg Germany; ^3^ Institute of Organic Chemistry, Laboratory for Sustainable Chemistry and Catalysis (LSusCat) Johannes Kepler University (JKU) Altenberger Straße 69 Linz 4040 Austria; ^4^ Inorganic Chemistry I, Ruhr University Bochum Universitätsstraße 150 44780 Bochum Germany; ^5^ Key Laboratory of Applied Surface and Colloid Chemistry Ministry of Education School of Chemistry and Chemical Engineering Shaanxi Normal University Xi'an 710119 China; ^6^ Center for Nanointegration Duisburg‐Essen (CENIDE) University of Duisburg‐Essen Duisburg Germany

**Keywords:** Ag‐BIAN, CO_2_ Electrolysis, MEA, molecular catalysts, zero‐gap cell

## Abstract

In recent years, CO_2_ electrolysis, particularly the electrochemical reduction of CO_2_ to CO in zero‐gap systems, has gained significant attention. While Ag‐coated gas diffusion electrodes are commonly used in state‐of‐the‐art systems, heterogenized molecular catalysts like bis‐coordinated homoleptic silver(I) N,N‐bis(arylimino)‐acenaphthene (Ag‐BIAN) complexes are emerging as a promising alternative due to their tunability and high mass activity. In this study, the influence of ink composition on the performance of Ag‐BIAN‐based GDEs in zero‐gap electrolyzers (ZGEs) are systematically explored at 60 °C and 600 mA cm⁻^2^. Sedimentation analyses across various solvents informed the selection of optimal solvent‐catalyst and solvent‐carbon additive combinations, streamlining the GDE optimization process and reducing associated costs and time. These results demonstrate that solvent choice and dilution state of the ink are critical factors impacting CO_2_ reduction, achieving faradaic efficiencies for CO production (FE_CO_) up to 67% at 600 mA cm⁻^2^ with catalyst loadings as low as 0.2 mg cm⁻^2^. These findings lay the groundwork for advancing from homogeneous H‐type cells to industrial ZGE systems through tailored ink engineering.

## Graphical abstract

Ink composition plays an even more crucial role than thought before in the activity of molecular electrocatalysts. By refining solvent‐catalyst and solvent‐carbon combinations for different Ag‐BIAN catalysts, up to 67% faradaic efficiency is achieved for CO production at 600 mA cm⁻^2^ with minimal catalyst loading, providing a pathway to enhance CO_2_ electrolysis in industrial applications, with a stable Ag(I) species.

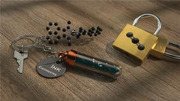

## 1 Introduction

In recent years, CO_2_ electrolysis has gained significant interest as a promising technology toward the creation of a closed carbon‐cycle.^[^ ^1^ ^]^ Especially, the electrochemical reduction of CO_2_ toward the generation of CO, in so called zero‐gap electrolyzers (ZGEs), in which the two half‐cells are separated by a thin ion‐exchange membrane, constitutes the most promising route. State‐of‐the‐art systems currently mainly feature Ag‐coated gas diffusion electrodes (GDEs), while heterogenized molecular systems have recently risen to the foreground as a tunable catalytic alternative associated with elevated mass activities for the production of CO.^[^ ^2^, ^3^, ^4^, ^5^, ^6^ ^]^

Here, we have recently demonstrated the promising ability of Ag(I)‐bis(arylimino)acenaphthene (Ag‐BIAN) complexes to reduce CO_2_ to CO at elevated current densities >300 mA cm^−2^ in industrially relevant ZGEs while demonstrating highly competitive mass activity values.^[^ ^7^, ^8^ ^]^ Notably, in our previous investigation we also showed that the observed trends between CO_2_ electrolysis experiments in the homogeneous state and the heterogenized state on a GDE were not directly transferable.^[^ ^7^, ^9^ ^]^ One postulated reason for this difference was the effect of agglomeration of the complexes when transferred to a GDE architecture, not allowing CO_2_ and electrons to efficiently reach the catalytic centers. This observation prompted us to take a step back from optimizing the electrode composition and focus on the starting point, i.e., the catalyst ink.

While catalyst inks containing molecular electrocatalysts are only a four‐component system, featuring the catalyst, ionomer, carbon, and solvent, numerous open questions still remain.^[^ ^10^, ^11^, ^12^, ^13^ ^]^ These knowledge gaps expand across the effect of employed solvent, degree of dilution in the ink, type, and amount of the carbon additive, among others, during CO_2_ electrolysis. Overall, it is currently unclear which ink‐component and respective value governs the obtained CO_2_R activity during the optimization of a GDE for CO production.^[^ ^13^, ^14^, ^15^ ^]^ Currently, studies focusing on these issues are limited, featuring mainly cobalt phthalocyanine‐based GDEs,^[^ ^16^, ^17^ ^]^ with little information currently available on how ink compositions can be further tuned toward other molecular variants, especially when aiming toward elevated current densities >300 mA cm^−2^.

We therefore set out to unravel of how different ink composition affect the efficiency of Ag‐BIAN‐based GDEs employed in industrially relevant ZGEs at 60 °C and at elevated current densities of 600 mA cm^−2^.^[^ ^18^, ^19^ ^]^ Notably, instead of directly testing different ink compositions, sedimentation studies in multiple solvents became the basis of our investigation,^[^ ^20^, ^21^ ^]^ allowing us to analyze and decipher the most promising solvent‐catalyst and solvent‐carbon additive interaction prior to the fabrication of GDEs, therefore decreasing the associated cost and time connected with such optimization efforts.

With the help of a coherent workflow, previously developed by us,^[^ ^20^ ^]^ we are able to show that the employed solvent during ink preparation as well as the used dilution play a crucial role in the observed performance. Our ink‐engineering allowed us to unlock the CO_2_ reducing capabilities of catalytic species previously considered inactive under such conditions, reaching FE_CO_ values of 67% at 600 mA cm^−2^ and a cell voltage of 3.1 V. Crucially, we are able to show that optimized Ag‐BIAN GDEs can maintain their Ag(I) oxidation state after electrolysis under such conditions, with our work creating a stepping‐stone toward bridging research between homogeneous H‐type cell investigations and studies in ZGEs operated under application‐relevant conditions.^[^ ^19^ ^]^

## 2 Results and Discussion

### 2.1 Sedimentation Studies

**Figure 1:**
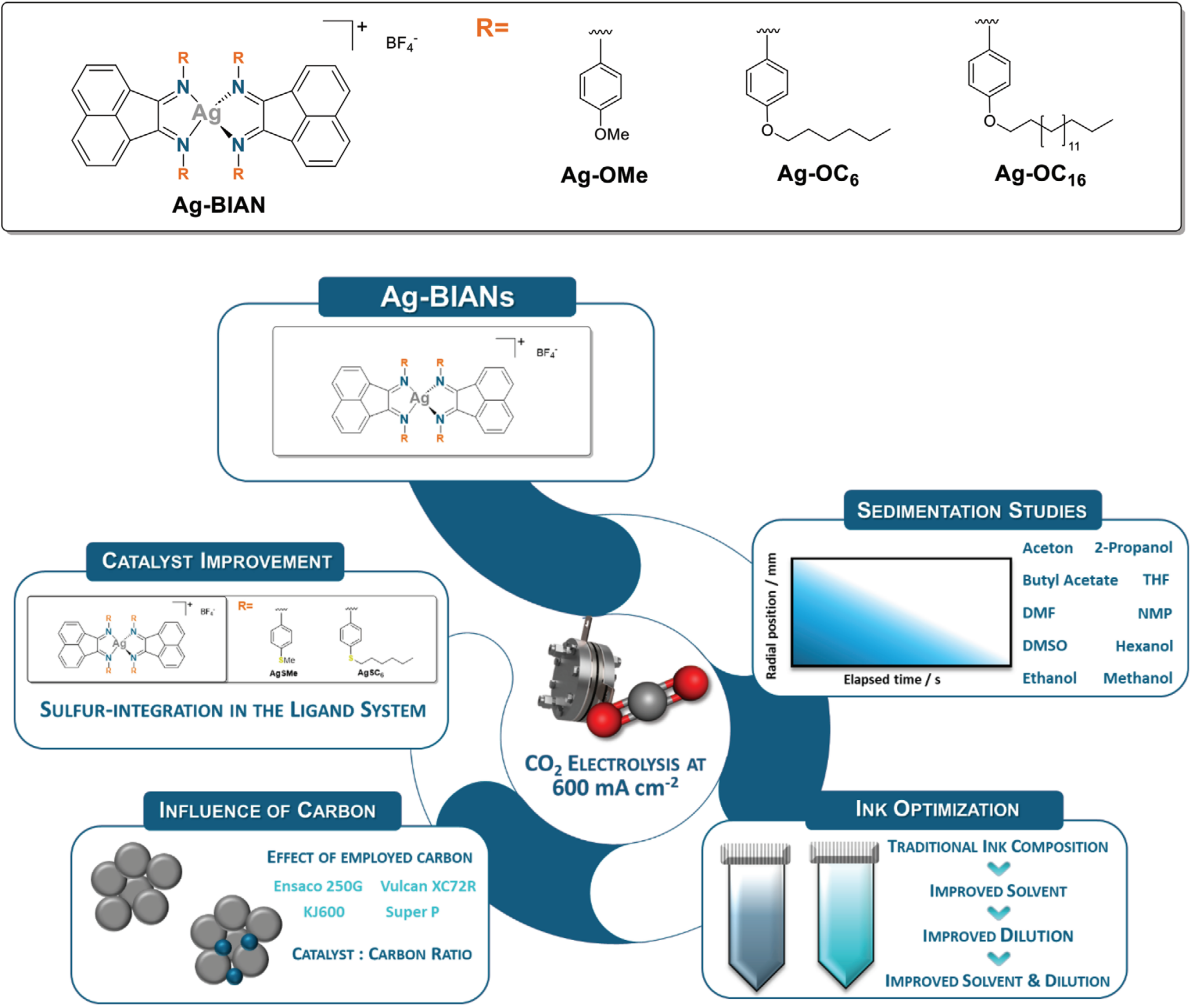
Schematic representation of the optimization procedure at 600 mA cm^−2^ developed and applied in this work.

Analytical centrifugation was employed to gauge the sedimentation characteristics of various carbon black materials in different solvents as well as their capability to disperse the utilized Ag‐BIAN catalyst. Here, three different variants of our Ag‐BIAN catalysts are investigated with alkyl chains of different lengths at the phenyl ring, ranging from methoxy‐ (AgOMe), to hexyloxy‐ (Ag‐OC_6_) and finally hexadecyloxy‐ (AgOC_16_) substituents (**Figure** **1**). This screening will better allow us to understand how catalyst inks need to be best tailored depending on the integrated substituents.

During a typical measurement, monochromatic light passes through the respective measurement cell while it is being centrifugated and an array of charge coupled devices measures the transmitted light at set intervals. The change in transmission values can then be visualized in transmittograms for a more intuitive understanding of the data.^[^ ^22^, ^23^, ^24^ ^]^ To measure the dispersion state of the catalysts in the solvents, 10 mg of the catalysts were added to 5 mL of the solvents and the solution was tip sonicated for 5 min at an amplitude of 20%. The heating characteristics of the tip sonicator at these conditions was investigated previously.^[^ ^25^ ^]^ During sonication, the vessel was cooled using ice water to prevent the solvent from evaporating. The ten solvents employed in this study (acetone (ACE), 2‐butoxyethyl acetate (BAc), dimethyl formamide (DMF), dimethyl sulfoxide (DMSO), ethanol (EtOH), hexane (HEX), isopropyl alcohol (IPA), methanol (MeOH), n‐methyl pyrrolidone (NMP), tetrahydrofuran (THF)) were chosen based on their Hansen solubility parameters (HSPs) and their ability to cover a large volume in the 3D Hansen space.^[^ ^20^ ^]^

**Figure 2:**
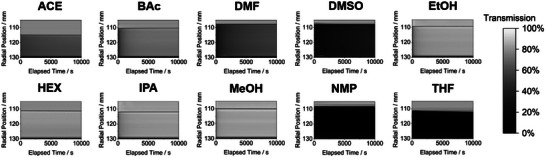
Transmittograms of solutions containing 10 mg Ag‐OC_16_ in 5 ml of the respective solvent. The transmission value legend can be found on the right.

Exemplary dissolution capacities of Ag‐OC_16_ in the chosen solvents can be seen in **Figure** **2**. The darker the value of the transmittograms, the more incident light is being blocked by the solution and the higher the dissolution capacity of the respective solvent. It is directly visible at a glance that NMP and THF possess the highest dissolution capacity based on how dark the transmittograms are and how the transmission value remains close to 0% over time. A non‐stable transmission value (such as with DMF) indicates that the molecular catalyst forms insoluble agglomerates, which are merely dispersed in the solvent and slowly sediment during the centrifugation process, being therefore not stable against agglomeration in the respective solvent. This distinction was achieved by setting the centrifugal force of the analytical centrifuge to be high enough to make particles down to ≈30 nm readily sediment but not high enough to force solvated molecules to migrate through the measurement cell. For the purposes of this study, a solvent is labeled as “good” when the transmission value stays <1% throughout the duration of the measurement and “poor” when it does not.

**Figure 3:**
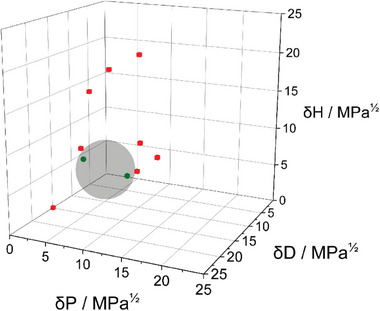
Exemplary Hansen sphere of Ag‐OC_16_.

The same measurements were conducted for Ag‐OMe and Ag‐OC_6_ (Figure S1, Supporting Information) and revealed that the best solvents are MeOH for Ag‐OMe and THF for Ag‐OC_6_ although both catalysts are significantly less selective about the employed solvent when compared to Ag‐OC_16_. This can be seen in the respective transmittograms and the radius of the corresponding Hansen sphere in **Table** **1**. The optimal solvent selection was made not only considering the dissolution capacity of the solvent, but also its boiling point. The boiling point needs to be low enough to allow for the solvent to evaporate without subsequently thermally decomposing the catalyst. Therefore, low boiling point solvents such as MeOH and THF were chosen for further electrocatalytic testing. The gathered transmission data can then be utilized to calculate the HSP of the catalysts to gain further insights into the surface properties of the utilized ligands, based on the principle that like dissolves like. The determined HSP are shown in Table 1. An exemplary Hansen sphere of Ag‐OC_16_ is shown in **Figure** **3**. The poor solvents are shown as red cubes while the good solvents are shown as green spheres. The coordinates and radius of the Hansen sphere are selected to encompass the good solvents within the sphere while excluding the poor solvents outside it. The remaining Hansen spheres of Ag‐OMe and Ag‐ OC_16_ can be found in Figures S2 and S3 (Supporting Information). Specially, in the case of good solvents, the catalyst particles are homogeneous dissolved, while for poor ones the molecules tend to rapidly form insoluble agglomerates, which rapidly sediment with the ink. As we have shown, such agglomerates tend to limit the CO_2_R activity of Ag‐BIAN catalysts.^[^ ^7^ ^]^

**Table 1:** HSP values of the analyzed catalysts.

Name	Ag‐OMe	Ag‐OC_6_	Ag‐OC_16_
Disperse Interactions δD in MPa^1/2^	15.6	13.5	17.3
Polar Interactions δP in MPa^1/2^	16.7	17.4	9.0
Hydrogen Bond Interactions δH in MPa^1/2^	14.1	9.9	7.3
Radius in MPa^1/2^	9.5	13.6	3.6
Wrong solvents in/out	0/0	0/0	0/0
Good solvents	MeOH, NMP, DMF, DMSO, ACE	MeOH, NMP, DMF, DMSO, ACE, THF, BAc	NMP, THF
Poor solvents	EtOH, IPA, THF, BAc, HEX	EtOH, IPA, HEX	EtOH, IPA, HEX, BAc, MeOH, DMSO, DMF, ACE

The calculated HSP show a clear trend regarding a higher affinity for hydrogen bonds with shorter ligand lengths. A jump in the affinity for polar interactions between Ag‐OC_16_ and Ag‐OC_6_ is visible as well. The selectivity of the solvent preference of the catalyst is reflected in the radius of the respective Hansen sphere, showing a trend where Ag‐OC_6_ is the least selective about the solvent and Ag‐OC_16_ is the most selective.

To measure the optimal dilution in the optimal solvent, the bottom of the ink vessels was visually inspected for undissolved material after each tip sonication procedure. The catalyst concentration was gradually increased and showed first signs of incomplete catalyst powder dissolution after ≈0.7 mg mL^−1^. As a result, 0.5 mg ml^−1^ were determined to be the optimal concentration. In addition to the catalysts, the sedimentation stability of the employed carbon black supports in the chosen solvents was also investigated to maximize the ink stability and the resulting surface area of the catalyst layers by minimizing the particle size of the carbon support. This can be seen in Figure S4 (Supporting Information). Within the context of the herein presented measurements, NMP, DMSO, DMF, EtOH, and THF showed the highest stability for ENSACO 250G and are thus suitable for further ink composition considerations. The remaining carbon black supports were investigated previously.^[^ ^26^ ^]^ We believe that maximizing the dissolution of the Ag‐BIAN as well as the dispersion of the employed carbon black, will allow for a higher electrical conductivity to the catalytic centers. To ensure that the ink remains stable when all components, including the various ionomers, are added, full‐ink level stability measurements were conducted (Figure S5, Supporting Information). All optimized catalyst inks remained stable throughout the duration of the measurement.

### 2.2 Electrochemical Investigation in Zero‐Gap Electrolysers

Notably, since Ag‐BIANs already showed elevated CO selectivity >90% at current densities of 300 mA cm^−2^,^[^ ^7^ ^]^ we decided to perform our electrolytic investigation at a higher current density of 600 mA cm^−2^, to further unravel possible effects of the ink composition while further pushing the limits of molecular electrocatalysts. All electrochemical investigations were performed in our previously optimized set‐up featuring an optimized humidification value in relation to the applied current density, as well as applying a λ_CO2_ of 12 (Figure S4, Supporting Information).^[^ ^7^, ^27^, ^28^ ^]^ To further improve upon the reproducibility and stability of the gained results, a step‐wise electrochemical protocol was applied, increasing the applied current density in 50 mA cm^−2^ steps, held for 30 s, prior to performing electrolysis at 600 mA cm^−2^ for 30 min (Figure S6, Supporting Information).

Since one of the most attractive points of Ag‐BIAN is their highly competitive mass activity, we herein decided to maintain a low loading of only 0.2 mg cm^−2^ of Ag‐BIAN, with the actual Ag‐loading varying across the different tested Ag‐BIAN (Table S1, Supporting Information). While Ag‐BIAN catalysts can be easily and scalably synthesized in a few steps, we believe that maintaining this low catalytic loading also allows us to even better counterbalance the synthetic cost/workload against the necessary amount of molecular catalyst for efficient CO_2_ electrolysis, further focusing on tuning their application toward industrially relevant environments.

**Figure 4:**
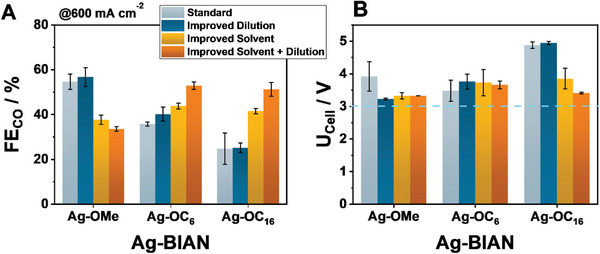
Achieved Faradaic Efficiency for the different ink compositions and Ag‐BIAN catalysts at 600 mA cm^−2^ at a loading of 0.2 mg cm^−2^ at 60 °C (A). Cell voltage obtained throughout the different Ag‐BIAN GDE variations (B). In the case of Ag‐OMe the improved solvent was MeOH, while in the case of Ag‐OC_6_ and Ag‐OC_16_ it was THF. No other products beyond CO and H_2_ were detected during electrolysis (Figure S7, Supporting Information).

To clearly assess the transferability of our sedimentation studies, Ag‐BIAN loaded GDEs were prepared in four different fashions: i) our previously reported variant employing ethanol as the solvent and maintaining a dilution value of 2 mg mL^−1^ for the Ag‐BIAN catalyst, ii) a diluted variation of 0.5 mg mL^−1^ in ethanol, as well as iii) and iv) the respective variants employing the most promising solvents from our sedimentation study, methanol for Ag–OMe, THF for Ag–OC_6_ and Ag–OC_16_ under the lower and higher dilution values (**Figure** **4**).

Observing both the obtained Faradaic Efficiency for CO (FE_CO_) and the respective cell voltage value (U_Cell_) the role of the employed ink‐parameters appears to be highly impactful and not equal among the different electrocatalysts. This effect is notably most evident in the case of Ag‐OC_16_. As previously highlighted, Ag‐OC_16_ shows significantly lower dispersibility in ethanol, the solvent employed in our “standard” GDE composition. Increasing the dilution value in this case from 2 to 0.5 mg mL^−1^ appears to have marginal influence on the observed activity. Nevertheless, employing THF as the ink‐solvent leads to a dramatic improvement with FE_CO_ values ranging from 41% to 51% for the undiluted and diluted THF‐variant, respectively. This improvement in the obtained FE_CO_ value is also accompanied by significant changes in the observed cell voltage value at 600 mA cm^−2^ decreasing from close 5.0 V in the case of the ethanol ink to 3.8 and 3.4 V when employing THF and its diluted variant.

A similar trend can be observed in the case of Ag‐OC_6_, with the FE_CO_ increasing from 35%, at the starting point of our investigation, to 52% in the diluted THF variant. Since, Ag‐OC_6_ does not show such a strict dispersibility spectrum as Ag‐OC_16_, the effect of the solvent on the U_Cell_ values is not so prominent (ranging around 3.4 V).

Notably the improved performance, from the observed for Ag‐OC_6_ and Ag‐OC_16_ is not mirrored in the case of Ag‐OMe. Transitioning from ethanol to methanol, the FE_CO_ value demonstrates a significant decrease from 56% to 37% as well as 33% when the dilution value is further increased. Interestingly, such changes in selectivity are heavily mirrored in the respective cell voltages, with the U_Cell_ values increasing by 100 mV when transitioning from EtOH (3.2 V) to MeOH (3.4 V) (Figure S8, Supporting Information). Interestingly, dilution in EtOH appears to lead to a decrease of the cell voltage from 4 to 3.2 V.

Crucially, this counter‐intuitive decrease in the observed performance can be attributed to the physicochemical properties of the employed carbon black, Ensaco 250G, instead of the Ag‐OMe catalyst. Additional sedimentation studies showed that Ensaco 250G exhibits an especially unstable behavior in MeOH (Figure S4, Supporting Information). This observation clearly highlights how the final GDE activity is not only the result of optimizing the ink‐composition around the molecular system but also considering the employed carbon support, which must properly “match” with the ink. Furthermore, whilst under the optimized ink conditions the different Ag‐BIAN GDEs show similar FE_CO_ values, the employed Ag‐loading varies among the complexes (Table S1, Supporting Information), leading to an increase of the obtained mass activity per deposited Ag‐center from about 15 000 mA_CO_ mg_Ag_^−1^ for Ag‐OMe to approximately 25 000 mA_CO_ mg_Ag_^−1^ for Ag‐OC_16_ (Figure S9, Supporting Information). Crucially, this elevated activity is the result of the direct co‐operation between the Ag center and the BIAN ligand. Through its non‐innocent character, the ligand is able to accept two electrons during electrolysis at the cathode, and acts in essence as an electron reservoir providing electrons to the silver center during the catalytic cycle.^[^ ^7^ ^]^

**Figure 5:**
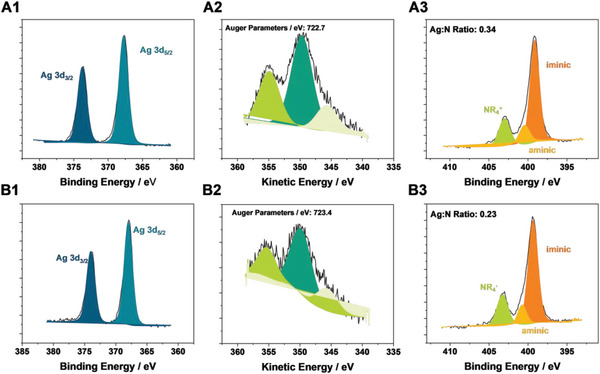
XPS analysis of the Ag‐OC_16_ GDEs prior (A) and post electrolysis (B) at 600 mA cm^−2^ and 60 °C in a ZGE.

Surprisingly, the post‐electrolysis analysis of the Ag‐OC_16_ GDEs via XPS by monitoring changes in the Auger parameters and the Ag:N ratio shows minimal changes compared to the pristine samples, showing the Ag(I) center maintains its oxidation state (**Figure** **5**). This result is in accordance with our previous quasi in situ investigations of Ag‐OC_16_ at −2.0 V versus RHE in 0.1 M KHCO_3_, having shown that Ag‐OC_16_ is able to maintain its molecular form due to the highly electron‐donating nature of the ligand.^[^ ^7^ ^]^ Most crucially, this interesting finding and elevated FE_CO_ values clearly demonstrate the crucial role of the correct ink solvent alongside the proper dilution to significantly elevate the activity of heterogenized molecular systems. On the other hand, independently of the electrode composition, Ag‐OMe and Ag‐OC_6_ follow the previously observed trends, with Ag‐OMe forming mainly Ag‐NPs and Ag‐OC_6_ a complex mixture of Ag‐NPs engulfed by the BIAN ligand (Figure S10, Supporting Information). In order to take full advantage of our XPS post‐mortem analysis, we tried to further analyze how the ink composition affects the Ag:N ratio as well as the overall stability of the Ag(I) center within the ligand. Specifically, the peak centered around 399.3 eV in the N_1s_ spectra is assigned to the iminoaryl‐groups bound to Ag^+^ and the second peak at 400.9 eV may be associated to the BIAN ligand without the Ag center, with comparable binding energies reported in the literature for similar ligands, as well as in our earlier work.^[^ ^7^, ^29^ ^]^ Assuming a constant amount of PiperION binder present on the electrode surfaces, the initially prepared ink (labeled as Standard) shows a significant loss of BIAN complex with and without Ag centers in the N_1s_ spectra (Figure S11, Supporting Information). The Ag‐OMe and Ag‐OC_6_ complexes show after dilution and improvements of the catalyst inks lower BIAN content than Ag‐OC_16_, which could have impacted the catalytic performance. Interestingly, for the best performing electrode Ag‐OC_16_ (with diluted and improved solvent), the peaks assigned to the iminoaryl‐groups only show a minor change in spectral contribution within 1% of the total signal intensity. This suggests that close to 85% of BIAN, which have an Ag center before electrolysis, retain this metal center also after the application at 600 mA cm^−2^.

Determination of the total Ag and N content of the electrodes through survey scans shows, that the best performing electrode Ag‐OC_16_ with an improved and diluted catalytic ink has a higher overall ligand content than most measured samples (2.2 and 2.6 at.% N before and after CO_2_R respectively, Figure S12, Supporting Information). As the Ag Auger parameter of the fully optimized Ag‐OC_16_ GDE also remains close to 623.3 eV, the complexes on these electrodes possibly could retain their structural integrity and stabilize the Ag(I) oxidation state through the presence of the BIAN ligand, leading to better CO_2_R performance. As an interesting side note, aiming here to better identify the actual concentration of electroactive species, we performed CVs in a flow cell in 0.1 M KHCO_3_ under an Ar‐atmosphere focusing on the 1‐electron reduction of the BIAN ligand (Figure S13, Supporting Information). Surprisingly compared to the ethanol ink, in which Ag‐OC_16_ showed low CO_2_R activity, the amount of electro‐active species is doubled compared to its THF‐counterpart (0.6 nmol cm^−2^), both lying in the 1 nmol cm^−2^ range, significantly lower than the 300 nmol cm^−2^ in the GDE itself. This observation is in stark contrast to our results of the analytical centrifugation, showing that aggregation should be majorly decreased and a large number of active centers must participate in catalysis to reach the obtained CO‐production values at 600 mA cm^−2^. In essence, this discrepancy points out the need to develop more tailored operando zero‐gap cells to concretely understand the chemical changes in molecular‐based GDEs.

#### 2.2.1 Influence of Satellite Components

In CO_2_R reports featuring molecular electrocatalysts, the employed carbon type, and catalyst:carbon ratio has often been cited as an additional tuning point for the obtained performance. While the influence of the employed carbon additive has been heavily investigated in the case of CoPc, this is not the case for other molecular variants. Here, we aimed to fill this gap by employing different carbon blacks, such as Super P, Ketjenblack EC‐600JD (KJ600), and Vulcan XC72R, which show different dispersibility profiles, alongside different characteristics regarding their hydrophobicity and surface groups.^[^ ^26^ ^]^

**Figure 6:**
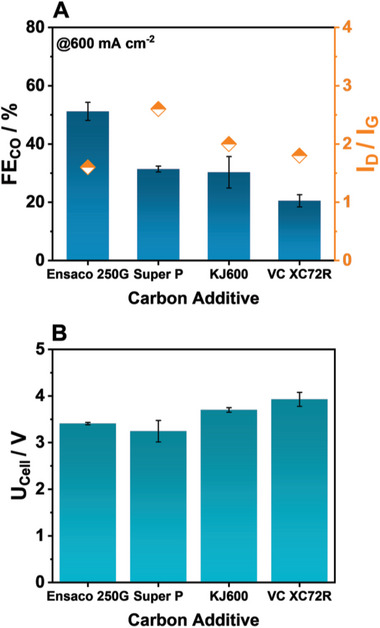
Role of the employed carbon material on the CO_2_R performance of Ag‐OC_16_‐based GDEs, generated through inks in THF with a dilution of 0.5 mg ml^−1^ after electrolysis at 600 mA cm^−2^.

Notably, employing different carbon materials other than Ensaco 250G leads to a decrease in the obtained FE_CO_ to ≈35% for Super P and KJ600, with Vulcan XC72R containing GDEs delivering a value of only 22% (**Figure** **6**). Crucially, in the case of KJ600 and Vulcan, the highest U_Cell_ value at 3.7 and 3.9 V, respectively is obtained. Especially, in the case of KJ600 this is surprising as it has been shown to possess an elevated dispersibility in THF compared to the other carbon materials.

Aiming to find some discerning differences, in Figure 6A we have also plotted the I_D_/I_G_ ratio of the different carbon blacks (yellow diamonds) according to literature values. ^27^From these, Ensaco 250G appears to possess the lowest I_D_/I_G_, denoting a high degree of graphitization and decrease of surface defects. An additional highlight of Ensaco 250G, according to the manufacturer Imerys, is its low water retention compared to acetylene black, with similar information not being available for the other carbon counterparts.^[^ ^30^ ^]^ With the balance between incoming H_2_O and CO_2_ in the electrolyzer having been shown to be crucial for the obtained activity, possibly the low water retention activity of Ensaco 250G could be the main driving force for its elevated performance in GDEs. Another noteworthy parameter is the total pore volume of the employed carbon blacks, reaching values of 0.18, 0.32, 0.67, and 2.47 cm^3^ g^−1^ for Ensaco 250G, Super P, XC72R, and KJ600, respectively.^[^ ^31^, ^32^ ^]^ Unfortunately, due to the lack of standardized investigations on the complete characterization of carbon black for electrolytic applications with suggested solutions just being reported in our recent work,^[^ ^26^ ^]^ it is difficult to pinpoint the clear reason regarding the observed trends, urging the community to cooperatively fill these gaps.

Furthermore, changes in the applied ratio between Ag‐OC_16_ and Ensaco 250G, did not yield an improvement but a decrease of the FE_CO_ from 51% to 43% and 33%, when transitioning from a catalyst:carbon ratio of 2:1, 1:1, and 1:2 respectively. Possibly at lower catalyst:carbon ratios the thickness of the catalyst layer increases. This thickness increase at which carbon is the most prominent element, possibly leads to a promotion of the parasitic HER, and significantly altering the local micro‐environment around the molecular catalytic centers, affecting also the observed U_Cell_ values (Figure S14, Supporting Information).

Moreover, the homogeneous dispersion of the ionomer within the catalytic layer could be another limiting factor. We thus also applied sedimentation tests for six different ionomers (PiperION, Sustainion XA‐9, XC‐1, XC‐2, Durion, Pention). Here the dispersibility of the ionomer was evaluated on its ability to better stabilize the sedimentation of the Ensaco 250G carbon black in THF, which is the most promising solvent for our mass activity‐focused investigations. Nevertheless, the transmittograms of all investigated ionomer dispersions did not show considerably different behavior compared to the most‐ionically conductive ionomer already employed in our work, PiperiON. This observation further shows how current ionomer dispersions must be further tailored for molecular electrocatalysts, which are not readily dispersible in common solvents, such as THF (Figure S5, Supporting Information). Moreover, comparing the influence of direct mixing against immobilization, we immobilized Ag‐OC_16_ both on Fe‐free CNTs and Ensaco 250G comparing the activity of the resulting GDEs with the directly mixed variant. Surprisingly, direct mixing of CNTs did not yield any improvements in terms of the FE_CO_ (34% with CNTs), whilst in the case of the immobilization route elevated U_Cell_ values >3 V and short‐circuits are observed for Ensaco 250 G and CNTs respectively (Figure S15, Supporting Information).

Overall, at elevated current densities > 300 mA cm^−2^ the effect of the satellite components such as the carbon support appears to be secondary to the activity of the employed electrocatalysts, with the ink‐composition and the resulting GDE architecture playing here a more crucial role. Evidently, improper choice of the employed carbon can have dramatic effects on the obtained CO_2_R activity, requiring more systematic studies focused specifically on CO_2_ electrolysis, involving an array of molecular electrocatalysts. Additionally, our comparison experiments do not necessarily denote that direct mixing of the carbon and molecular catalysts fully outweighs immobilization routes, rather strongly hinting that depending on the fabrication routes different ink compositions must be developed.

#### 2.2.2 Closing the Optimization Cycle by Further Catalytic Tailoring

In prior research by our group a direct correlation between the electron donating properties of the employed BIAN ligand and the catalytic activity of a silver(I) metal center for the electrochemical reduction of CO_2_ has been demonstrated.^[^ ^8^ ^]^ Thus we hypothesized that substituting the oxygen atoms in the already exceedingly performant Ag‐BIANs that we explored with more Lewis basic sulfur should augment their reactivity even further.^[^ ^7^ ^]^ Additionally, previous studies involving Ni‐Cyclam derivatives have shown that the inclusion of S in the ligand moiety can lead to more active electrocatalysts.^[^ ^33^, ^34^ ^]^ Inspired by this idea, we synthesized the S‐bridging variants of our complexes namely Ag‐SMe and Ag‐SC_6_, and tested them under the optimized conditions. Initial evidence that sulfur increases the electron density at the silver centers was observed in UV–vis spectroscopy, where the lowest energy absorptions of the sulfur‐containing complexes were bathochromically shifted, indicating a lower‐lying HOMO in Ag‐SC_6_ compared to Ag‐OC_6_.^[^ ^8^ ^]^ Furthermore, the alkylthio groups help reduce phase transition temperatures and inhibit aggregate formation in organic molecules. This is primarily due to the increased conformational flexibility, steric bulk, and reduced molecular anisotropy introduced by the C–S–C linkages. Additionally, the sulfur atoms promote intermolecular attractive interactions, reflected in phase transitions as spatially averaged phenomena, such as dispersion forces and more polarizable π‐systems. These interactions become more pronounced as the alkyl chain length increases. Serendipitously, our theory has furthermore manifested to hold true for CO_2_ electrolysis as exchanging oxygen for the more electron‐donating S leads to a direct increase in the observed activity in our ZGE set‐up. It is important to note that here we assumed that both Ag‐SMe and Ag‐SC_6_ follow the same dissolution behavior as their oxygen linked counterparts.

**Figure 7:**
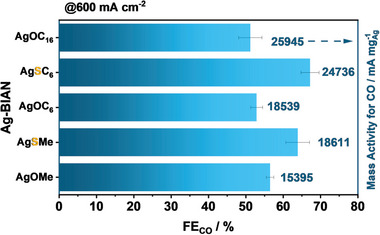
Comparison of the FE_CO_ value achieved for oxygen as well as sulfur‐containing Ag‐BIAN catalysts at 600 mA cm^−2^. On the right Y‐axis the mass activities of the different complexes are also given.

Transitioning from Ag‐OMe to Ag‐SMe, the FE_CO_ value increases from 56% to 64%, with the ameliorating effect being mirrored also in the case of Ag‐SC_6_, reaching an FE value of 67% at 600 mA cm^−2^. Notably, in the case of Ag‐OC_6_, this improvement in the FE_CO_ value is accompanied by a significant improvement in the obtained U_Cell_ value of 500 mV, obtaining a value of 3.1 V at 600 mA cm^−2^ (**Figure** **7**).

In terms of mass activity, the Ag‐SC_6_ complex is able to reach a value of 24 736 mA mg_Ag_^−1^ lying close to Ag‐OC_16_ at a value of 25 945 mA mg_Ag_^−1^, albeit at a much higher FE_CO_ value. Overall, with the help of our coherent workflow, the herein presented systems constitute one of the most selective and mass active electrocatalysts, especially among the molecular variants, in current literature (Table S2, Supporting Information).^[^ ^35^, ^36^, ^37^, ^38^ ^]^ Notably, we highlight how this performance is not only the result of testing a more active electrocatalyst but tailoring the complete ink environment around it and the employed carbon black support, which must respectively also show an elevated graphitization factor and low water retention as our comparison data suggests. It is nevertheless important to point out that further operando characterizations are necessary to further elucidate the catalytic species in the case of these new Ag‐BIAN compounds and to understand how they interact with their microenvironment.

## 3 Conclusion

Herein, we demonstrate how sedimentation studies by analytical centrifugation can become an important tool toward unveiling the most promising ink‐solvent and respective dilution state for the generation of highly active Ag‐BIAN based catalytic layers. We are able to show that in the case of Ag‐BIANs the optimal solvent strongly depends on the structure of the ligand environments, highlighting how maintaining an elevated dilution state of 0.5 mg mL^−1^ can unlock the CO_2_R activity both in terms of achieved FE_CO_ as well as cell voltage. Moreover, we investigate on the effect of secondary ink components, such as the carbon type, and catalyst‐ratio, showing how improper choice and combination of ink solvent and carbon can have dramatic effects on the obtained activity for CO_2_R. The herein presented coherent workflow allowed us to achieve a FE_CO_ value of 67% at 600 mA cm⁻^2^ and 3.1 V, constituting simultaneously one of the most mass active and selective molecular systems in literature. Most notably, we are able to show that our electron‐rich Ag‐BIAN Ag‐OC_16_ is able to maintain Ag(I) oxidation state during electrolysis, being one of the few cases in current literature that a molecular rather than a metallic species is responsible for the CO_2_R activity.

We therefore would like to present a holistic approach for the integration of molecular catalysts directly into GDEs, summarized in the following steps:

Step 1) Successful synthesis, and quantification of the CO production in H‐type cell experiments.
Step 2) Sedimentation studies at low and high dilution (here: 2 and 0.5 mg mL^−1^ could be used as initial starting values for most molecular systems) in different solvents to locate the optimal solvent type for the molecular electrocatalyst and employed carbon.
Step 3) Creating a mix‐and‐match‐table between solvents and ink components.

- In the case of alcohols, the employed ionomer and ionomer content on the GDE can be improved.
- In the case of non‐commonly used solvents for the dispersion of ionomer, either in‐house generated dispersions from the solid resin can be generated, or the catalyst loading on the GDE can be varied.

Step 4) Variation of the surrounding parameters such as the relative humification of the gas stream.
Step 5) Fabrication of optimized electrocatalysts, integrating different functional groups as well as different immobilization approaches, setting of again the holistic pathway (Step 1).

## Conflict of Interest

The authors declare no conflict of interest.

## Supporting information



Supporting Information

## Data Availability

The data that support the findings of this study are openly available in Zenodo at https://doi.org/10.5281/zenodo.14012211.
