# Supplementary material for: Unlocking the Activity of Molecular Assemblies for CO2 Electroreduction in Zero‐Gap Electrolysers via Catalyst Ink Engineering
Source: Small. 2024 Oct 31;21(8):2408154. doi: 10.1002/smll.202408154 (PMC11855236; doi:10.1002/smll.202408154)
Supplement: Supplementary file 1 — Supporting Information [file SMLL-21-2408154-s001.pdf]

NANO · MICRO
small

Supporting Information

for *Small*, DOI 10.1002/smll.202408154

Unlocking the Activity of Molecular Assemblies for CO_2 Electroreduction in Zero-Gap Electrolysers via Catalyst Ink Engineering

Kevinjeorjios Pellumbi, Mena-Alexander Kräenbring, Dominik Krisch, Wiebke Wiesner, Sebastian Sanden, Daniel Siegmund, Fatih Özcan, Kai junge Puring, Rui Cao, Wolfgang Schöfberger, Doris Segets* and Ulf-Peter Apfel**

Supporting Information for:

Unlocking the Activity of Molecular Assemblies for CO_2 Electroreduction in Zero-Gap Electrolysers via Catalyst Ink Engineering

Kevinjeorjios Pellumbi,[A] Mena-Alexander Kräenbring†,[B] Dominik Krisch†,[C] Wiebke Wiesner,[D] Sebastian Sanden,[D] Daniel Siegmund,[A,D] Fatih Özcan,[B] Kai junge Puring,[A] Rui Cao,[E] Wolfgang Schöfberger,[C] Doris Segets*,[B,F] Ulf-Peter Apfel*[A,D]*

[A] Kevinjeorjios Pellumbi, Daniel Siegmund, Kai junge Puring, Ulf-Peter Apfel
Fraunhofer Institute for Environmental, Safety and Energy Technology UMSICHT, Osterfelderstraße 3, 46047 Oberhausen, Germany E-mail: ulf-peter.apfel@umsicht.fraunhofer.de

[B] Mena-Alexander Kräenbring, Fatih Özcan, and Doris Segets
Institute for Energy and Materials Processes - Particle Science and Technology (EMPI-PST), University of Duisburg-Essen, Carl-Benz-Straße 199, 47057 Duisburg, Germany, E-mail: doris.segets@uni-due.de

[C] Dominik Krisch and Wolfgang Schöfberger
Institute of Organic Chemistry, Laboratory for Sustainable Chemistry and Catalysis (LSusCat), Johannes Kepler University (JKU), Altenberger Straße 69, 4040 Linz, Austria
E-mail: wolfgang.schoefberger@jku.at

[D] Wiebke Wiesner, Sebastian Sanden and Ulf-Peter Apfel
Inorganic Chemistry I, Ruhr University Bochum, Universitätsstraße 150, 44780 Bochum, Germany E-Mail: ulf.apfel@rub.de

[E] Rui Cao
Key Laboratory of Applied Surface and Colloid Chemistry, Ministry of Education, School of Chemistry and Chemical Engineering, Shaanxi Normal University, Xi'an 710119, China
E-Mail: ruicao@snnu.edu.cn

[F] Doris Segets
Center for Nanointegration Duisburg-Essen (CENIDE), University of Duisburg-Essen, Carl-Benz-Straße 199, 47057 Duisburg, Germany E-mail: doris.segets@uni-due.de

Experimental Section

Materials and Methods

All chemicals were purchased from Sigma-Aldrich, Strem, BLDpharm, TCI or Merck and used without further purification unless stated otherwise. Anhydrous ACN was obtained from a molar sieve MB-SPS-7 (M. Braun Inertgas-Systeme GmbH) under argon atmosphere. The deuterated NMR solvents $CDCl_3$ and CD_3CN were obtained from Euriso-Top. Proton (^1H NMR) and carbon (^{13}C NMR) nuclear magnetic resonance spectra were recorded on a Bruker Advance 300 MHz NMR spectrometer and a Bruker DRX 500 MHz spectrometer equipped with a cryoprobe (TXI). The chemical shifts are given in parts per million (ppm) on the delta scale (δ) and referenced to the employed deuterated solvent for ^1H NMR and ^{13}C NMR spectra. UV-vis absorption spectra were collected on a Varian CARY 300 Bio spectrophotometer from 200 to 900 nm at ambient temperatures. High resolution mass spectra were obtained utilizing an Agilent 6520 Q-TOF mass spectrometer with an ESI source, an Agilent G1607A coaxial sprayer and ammonium formate containing eluents.

Preparation of 4-*n*-hexylthioaniline

4-Amino-thiophenol (4.35 g, 33.7 mmol) was dissolved in 175 mL dry ACN in a flame-dried Schlenk flask, cooled in an ice-bath and KO*t*bu (4.73 g, 42.2 mmol, 1.25 eq.) added under argon. The resulting light pink suspension was stirred for 1 h while being allowed to warm to rt. Ensuing, 1-iodhexane (7.51 g, 35.4 mmol, 1.05 eq.) was added and the mixture stirred for further 21 h at rt under argon. Afterwards it was extracted with DCM (300 mL) and water (200 mL) with the addition of brine to enhance phase separation. The organic extract was washed with brine (200 mL), filtered over a silica plug, dried over Na_2SO_4 and finally concentrated *in vacuo* to afford the desired product as brown-yellow oil in quantitative yield and sufficient purity for the next synthetic step.

General Procedure for the Preparation of BIAN Ligands

BIAN ligands were synthesized via the methodology established by the group of Ragaini.[1] In general, acenaphthenequinone and zinc(II) chloride (2.7 eq.) were stirred in acetic acid (0.25M) at 80 °C under argon for 30 min. Subsequently, 2.2 eq. of the respective aniline were added and the mixtures refluxed for 15 (OMe, SMe) or 20 min (OC6, OC16, SC6) leading to the formation of colorful BIAN ligated zinc(II) dichloride precipitates, which were collected via suction filtration. The residues were washed with cold HOAc (2 mL/100 mg acenaphthenequinone) and Et_2O (10 mL/100 mg acenaphthenequinone) before being extracted with DCM and saturated aqueous potassium oxalate solution (5 mL/100 mg acenaphthenequinone). Finally, the organic layers were dried over Na_2SO_4 and concentrated *in vacuo* to obtain the free BIAN ligands.

General Procedure for the Preparation of $[Ag(I)(BIAN)_2]BF_4$ Complexes

Homoleptic Ag(I) BIAN complexes were prepared in analogy to a literature procedure.[2] $[Ag(I)(ACN)_4]BF_4$ (0.2 mmol) was dissolved in 10 mL DCM and then treated with solutions of BIAN ligand (0.4 mmol, 2 eq.) in 20 mL DCM, leading to immediate color changes. The mixtures were subsequently stirred at room temperature in the dark for 15 min, before the reaction volumes were filtered and subsequently concentrated *in vacuo* to yield the respective pure Ag(I) bis-BIAN complexes.

Preparation of the gas diffusion Electrodes and different catalyst layers

For the preparation of the Ag-BIAN inks at a dilution value of 0.5 mg ml^{-1}, 15 mg of the respective Ag-BIAN catalyst, were mixed with 7.5 mg of carbon black as well as 75 µL of PiperION A5 Ionomer (Versogen), adding finally 30 mL of the employed solvents

All inks were sonicated in an ice-bath for 30 min and sprayed directly with the help of an Iwata Takumi Air-brush ontp 410 -µm-thick carbon cloth (W1S1011, Fuel Cell Store) at 80°C until a total Ag-BIAN loading of 0.2 mg cm^{-2} was achieved.

In the case of the immobilization comparison experiments, Ag-OC_{16} inks were prepared in THF similarly to the above-mentioned procedure, without the addition of ionomer. After sonication for 1 h and stirring overnight, the solvent was removed and EtOH was used as the ink solvent employing again a dilution value of 0.5 mg mg^{-1}.

The cathodes were cut with a punching iron of 16 mm diameter directly resulting in an active area of 2 cm^2.

Electrochemical investigation

Electrochemical investigation were performed at our previously developped set-up. [3] The cell was place in an oven together with the 0.1 M CsOH anolyte at 60°C and allowed to condition. For the detection of changes in the volume of the gas flow due to CO_2 crossover through the AEM and gas-forming reactions an Argon flow of 10 mL min^{-1} (9 vol.%) was added as an internal standard to the 100 mL min^{-1} CO_2 flow (λ_{CO2}: 12). Both inlet gases were controlled by mass flow controllers (Bronkhorst). For the humidification of the inlet gas stream, a bubbler filled with MilliQ water (18.2 MΩ · cm) was put into a temperature-controlled water bath at 65 °C outside of the oven. A slight overpressure of 100 mbar(g) was applied on the cathode side through the help of a back-pressure controller (Specken & Drumag). The anode circuit flow was controlled by a peristaltic pump at 100 mL min^{-1} using 0.1 M CsOH as the anolyte. For all experiments, the cell was operated with the anode facing upwards and the cathode facing downwards. The cathode and anode substrate flows were directed through the cell in a counterflow arrangement meaning that the directions of the gas inlet flow and the anolyte flow are opposite to each other.

For the performance of electrolytic experiments, a step-wise protocol was employed. After allowing the cell to condition for 10 minutes within the oven, conditioning steps of 50 mA cm^2

were applied directly after one another and held for 30 s prior to performing electrolysis at 600 mA cm^{-2} for 30 minutes.

Product analysis

The product gas composition was determined by online gas chromatography coupled with a mass spectrometer. A Shimadzu GC-MS-QP2020 equipped with a Supelco Carboxen 1010 PLOT Column was used with samples being taken every 20 min. For the long-term experiment, samples were collected every 2 h.

For the gas product quantification, faradic efficiency (FE_i) was calculated as follows:

$$FE_i = \frac{zF}{i} \frac{x_{Ar,inout}}{x_{Ar,output}} \frac{pFv}{RT}$$

where z is the number of the transferred electrons for the formation of each product, F is the Faraday constant, F_V is the volumetric flow of CO_2 and Ar, p is the applied back-pressure, R the gas constant and T the temperature, i is the total applied current, with the mole fraction of the generated products being corrected by the mole fractions of the internal Ar standard.

Analytical Centrifugation

All measurements were conducted using an LUMiSizer 6514-14 (LUM GmbH) at 2000 rpm and 22 °C using monochromatic light with a wavelength of 870 nm. To measure the dissolution capacity of the catalysts in the solvents, 10 mg of the catalysts were added to 5 ml of the solvents and the solution was tip sonicated using a Branson SFX550 tip sonicator for 5 min at an amplitude of 20 %. During sonication, the vessel was cooled using ice water to prevent the solvent from evaporating. The ten solvents employed (acetone (ACE), 2-butoxyethyl acetate (Bac), dimethyl formamide (DMF), dimethyl sulfoxide (DMSO), ethanol (EtOH), hexane (HEX), isopropyl alcohol (IPA), methanol (MeOH), n-methyl pyrrolidone (NMP), tetrahydrofuran (THF)) in this study were chosen based on their Hansen solubility parameters (HSPs) and their ability to cover a large volume in the 3D Hansen space. All solvents were bought from VWR and used without further purification.

Characterisation *via* X-ray Photoelectron Spectroscopy

X-ray photoelectron spectra were recorded using a Nexsa G2 Surface Analysis System (Thermofischer) equipped with a monochromated and micro-focused Al Kα X-ray source and a 180°, double-focusing, hemispherical analyzer with a 128-channel detector. The samples were analyzed using a band pass energy of 50.0 eV for high resolution elemental spectra and 200.0 eV for survey scans with a 0.2 eV measurement interval. The resulting peaks were fitted using

a Shirley-type background and an asymmetric Lorentzian lineshape, as implemented in CasaXPS 2.4.24. All sample spectra were calibrated against adventitious carbon, which was set to 284.8 eV.

To determine the oxidation state of Ag, the Auger parameters were calculated for each sample and are listed at the corresponding $AgM_5N_{45}N_{45}$ spectra. As reference, we measured an Auger parameter of 725.4 for metallic silver and 724.6 for Ag_2S in our previous work.

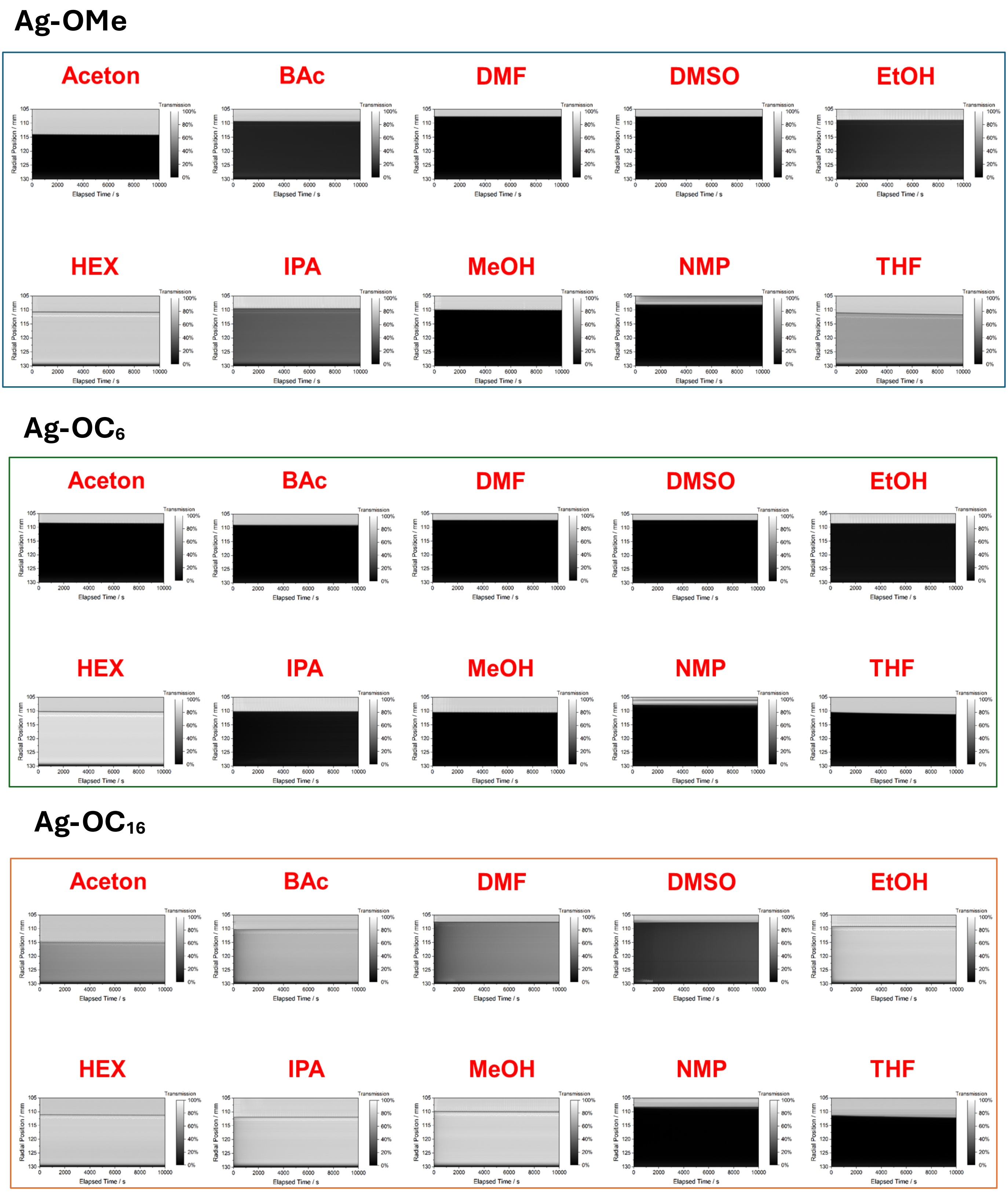

Figure S 1: Transmittograms of the herein investigated Ag-BIAN complexes in the different solvent with the help of analytic centrifugation at a dilution value of 2 mg mL^{-1}.

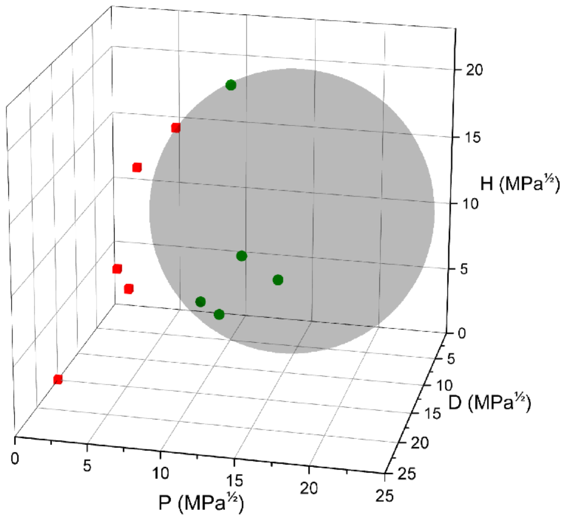

AgOMe							
	In= 5 Out= 5 Total= 10	Good:	MeOH	NMP	DMF	DMSO	Aceton
	δD= 15.6 δP=16.7 δH=14.1	Bad:	EtOH	i-PrOH	THF	BAc	Hexane
	δTot = 26.8 R= 9.5						
	Fit= 1.000						
	Core= ±[0.75, 0.70, 0.55]						
	Wrong In= 0						
	Wrong Out= 0						

Figure S 2: Hansen sphere of Ag-OMe.

.

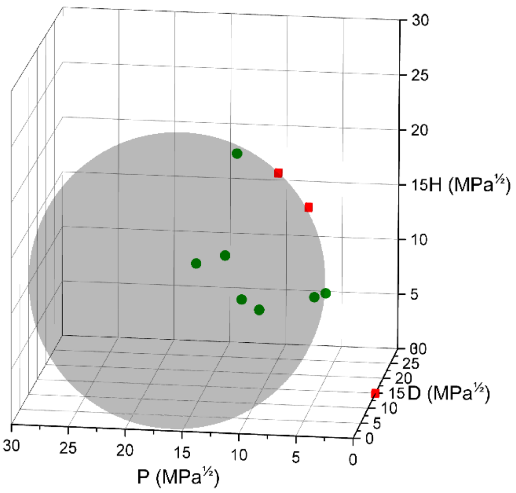

AgOC6									
	In= 7 Out= 3 Total= 10	Good:	MeOH	NMP	DMF	DMSO	Aceton	THF	BAc
	δD= 13.5 δP=17.4 δH=9.9	Bad:	EtOH	i-PrOH	Hexane				
	δTot = 24.1 R= 13.6								
	Fit= 1.000								
	Core= ±[1.35, 1.40, 0.40]								
	Wrong In= 0								
	Wrong Out= 0								

Figure S 3: Hansen sphere of Ag-OC_6.

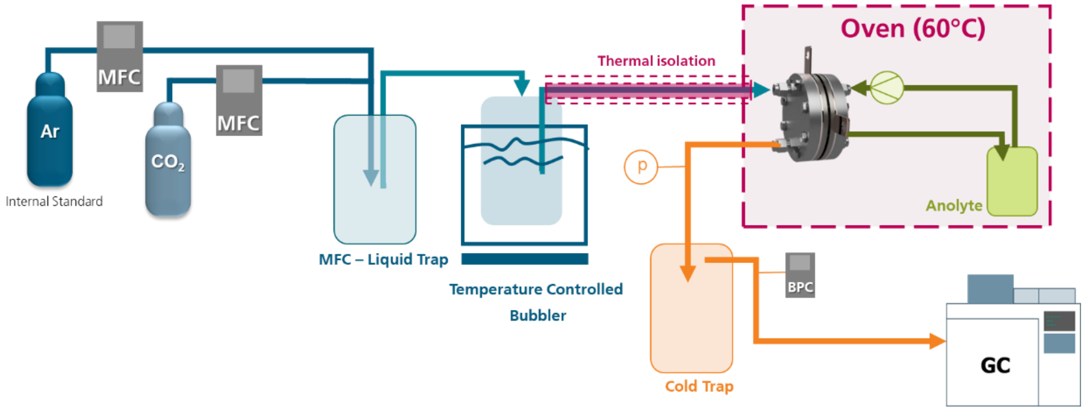

Figure S 4: Schematic represenation of the CO_2 electrolysis set-up employed in this work.

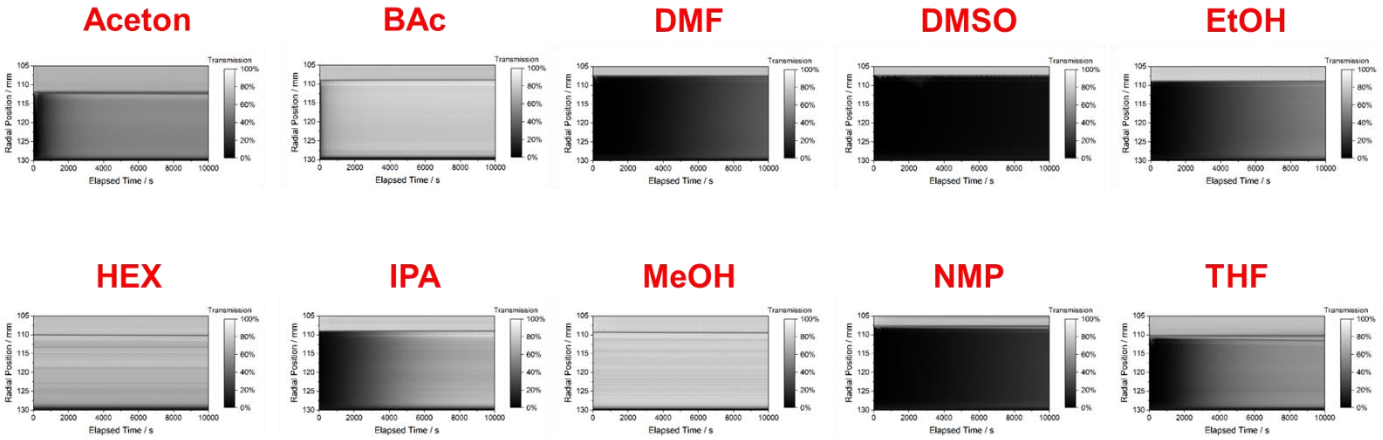

Figure S 5: Transmittograms of the ENSACO 250G in the different solvent with the help of analytic centrifugation at a dilution value of 1 mg mL^{-1}.

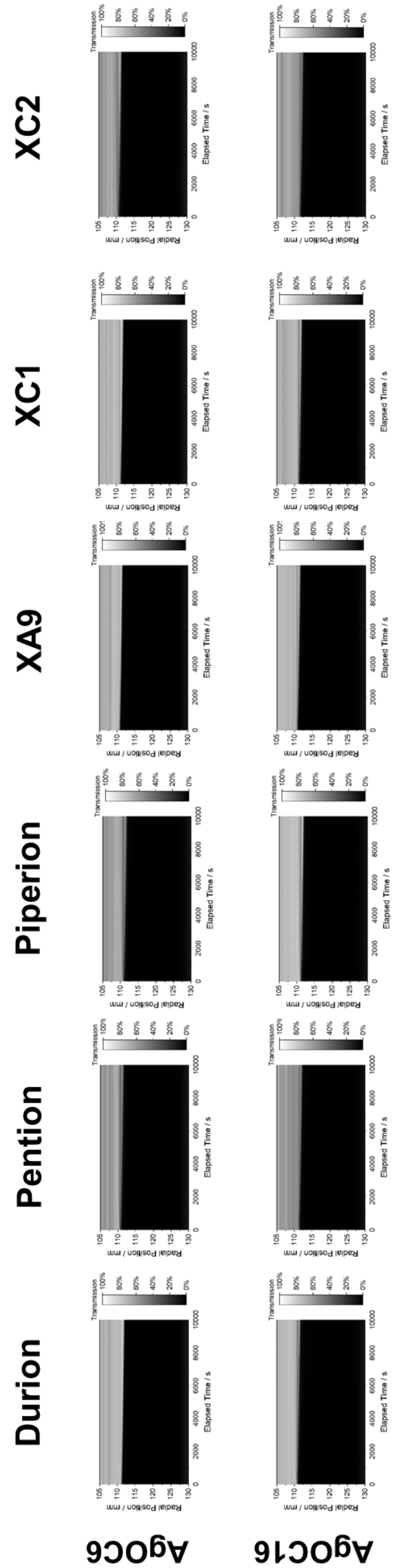

Figure S 6: Transmittograms of different commercially available ionomer mixed with ENSACO 250G and Ag-OC_6, Ag-OC_{16} in THF, aimed at finding an even better dispersible ionomer solution in THF compared to PiperION A5.

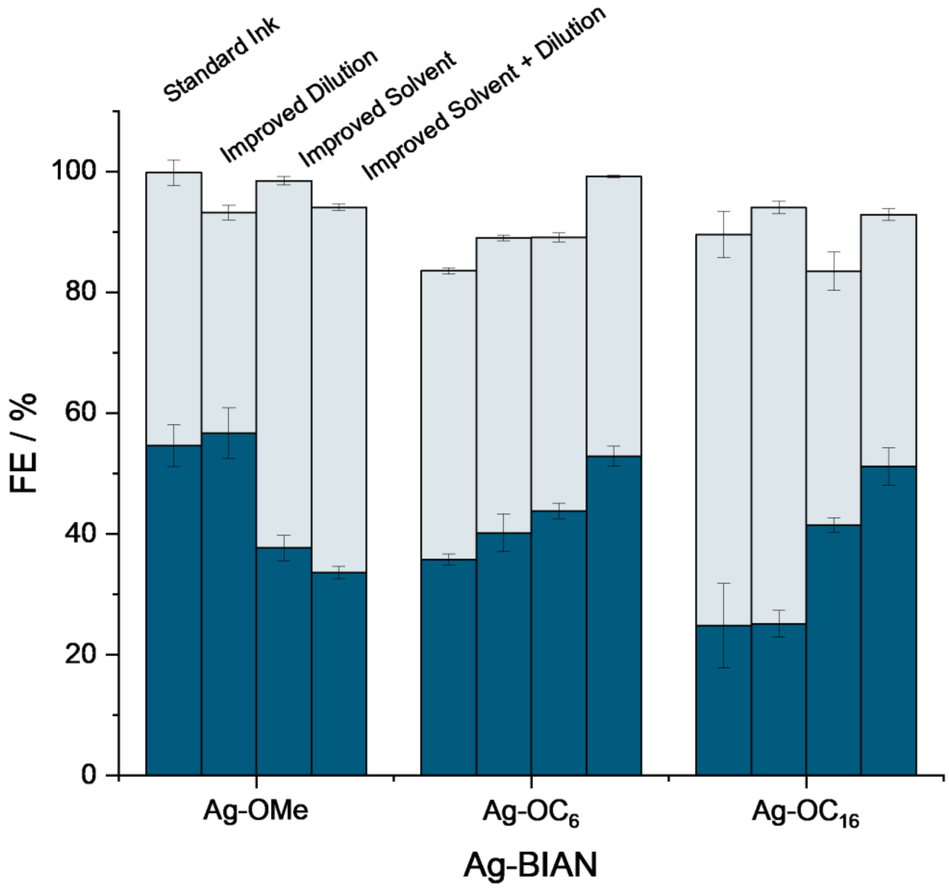

Figure S 7: Products spectrum obtained after electrolysis at 600 mA cm^{-2}. No liquid products were detected

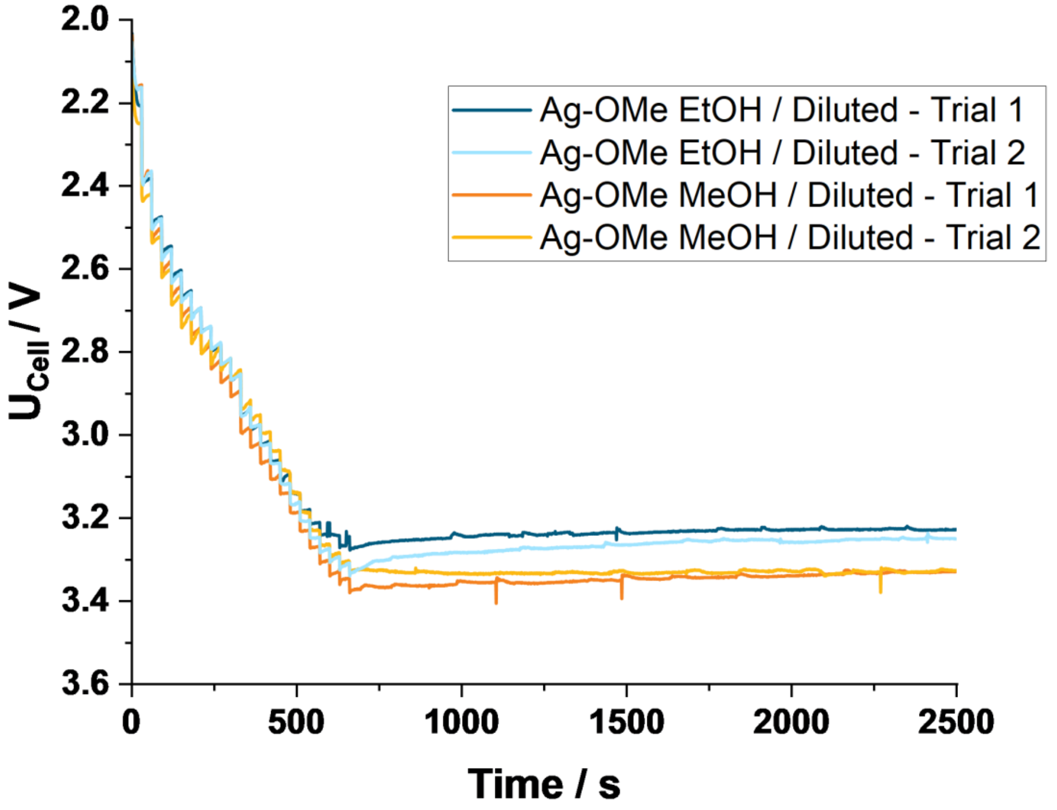

Figure S 8: Exemplary cell voltage curves demonstrating the applied electrochemical protocol from Ag-OMe in two different ink compositions.

Table S1 Structure and characteristics of the different Ag-BIAN catalysts.

**Catalyst**	**Structure**	**Molecular Weight / mol g^{-1}**	**Molecular Formula**	**Wt% Metal**
Ag-OMe	$[Ag(L)_2]BF_4$ L= 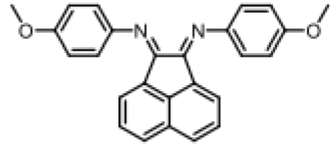	979.59	$AgC_{52}H_{40}N_4O_4$ BF_4	11.01
Ag-OC$_6$	$[Ag(L)_2]BF_4$ L= 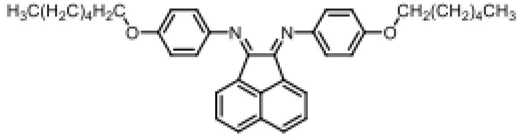	1260.10	$AgC_{72}H_{80}N_4O_4$ BF_4	8.56
Ag-OC$_{16}$	$[Ag(L)_2]BF_4$ L= 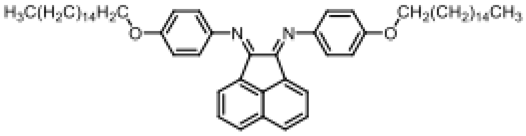	1821.17	$AgC_{112}H_{160}N_4$ O_4BF_4	5.92

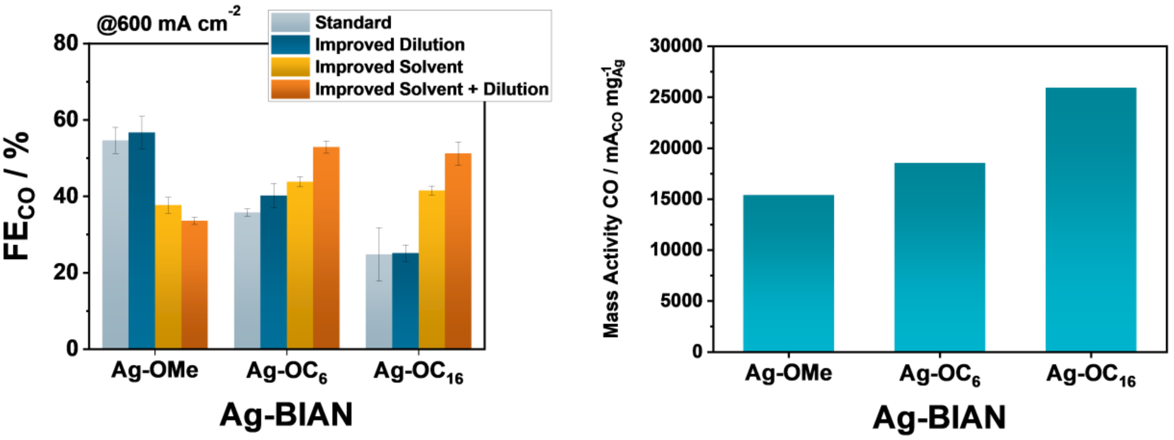

Figure S 9: Comparison of the achieved FE_{CO} within the different ink composition of the Ag-BIAN catalysts, alongside the achieved mass activity under the optimal conditions at 600 mA cm^{-2}.

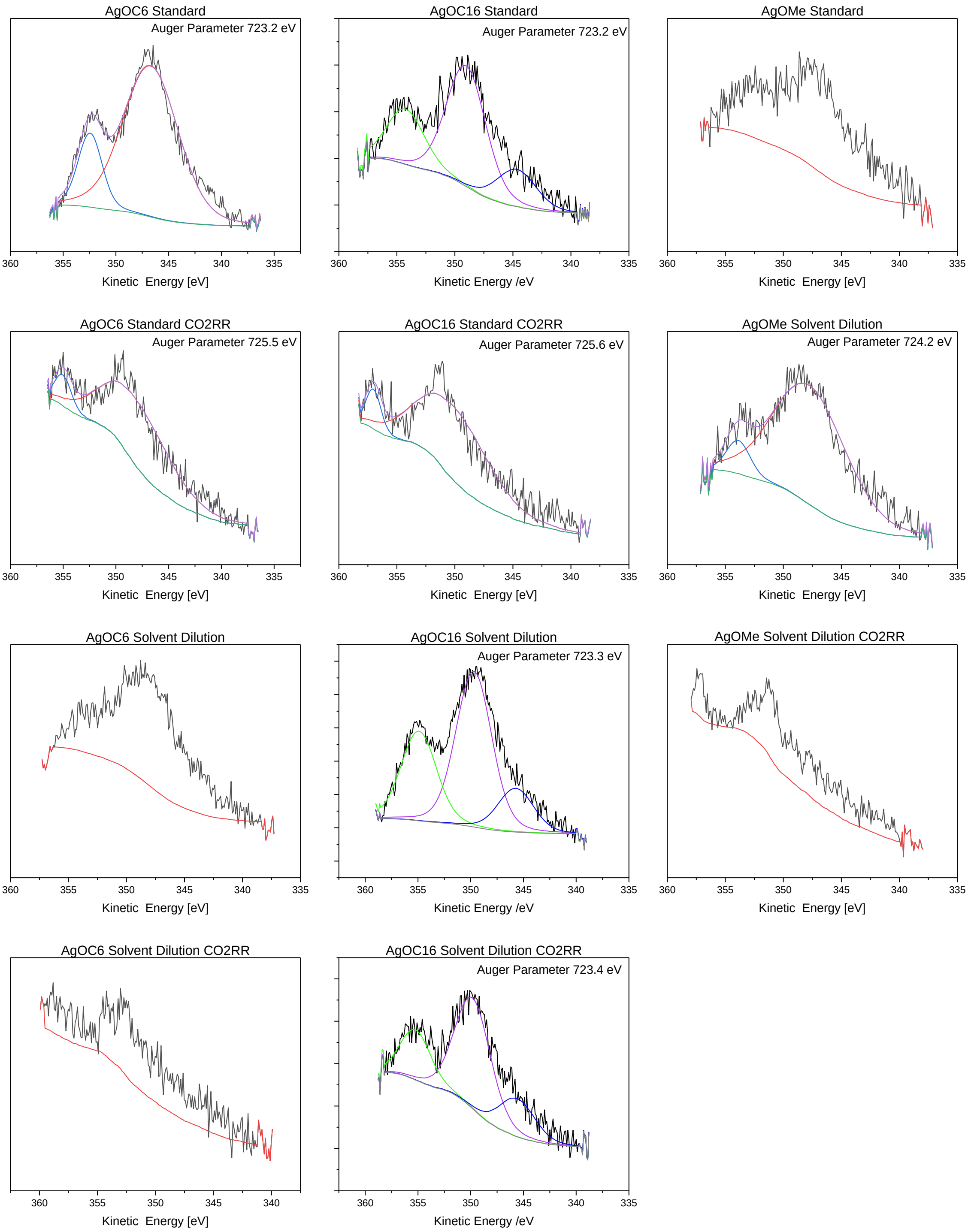

Figure S 10: Ag $M_5N_{45}N_{45}$ X-ray photoelectron spectra of and BIAN gas diffusion electrodes as prepared and electrodes to which a current of 600mA cm^{-2} was applied (labeled as CO2RR). The calculated Auger parameter is indicated in the respective panel.

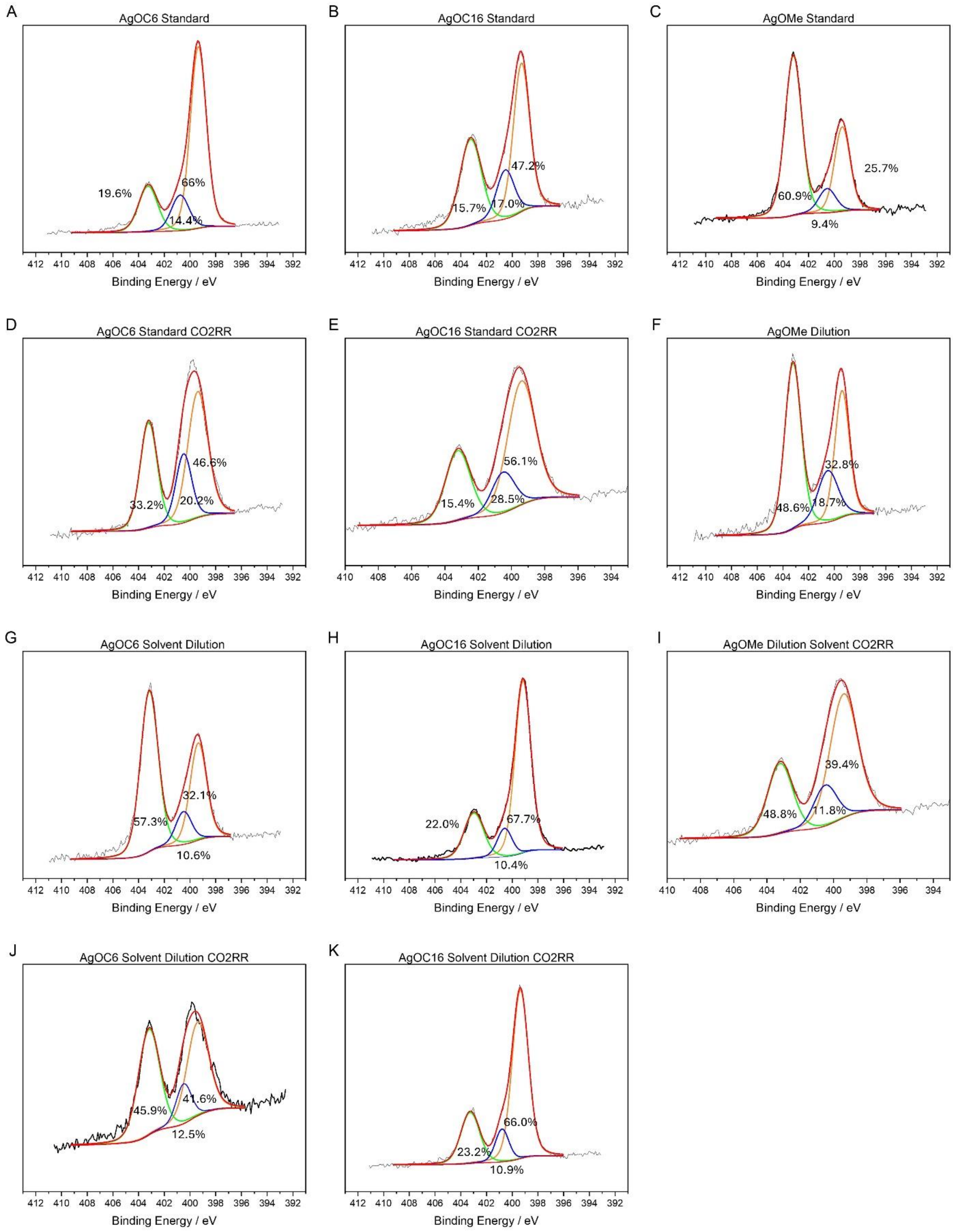

Figure S 11: N1s X-ray photoelectron spectra of BIAN gas diffusion electrodes as prepared and electrodes to which a current of 600mA cm^{-2} was applied (labeled as CO2RR). The orange peak is assigned to the arylimino-groups of BIAN 399.3eV, blue to the demetallated complex (400.9 ev) and green to the quaternary amine of the PiperIon binder at 403.45 eV.

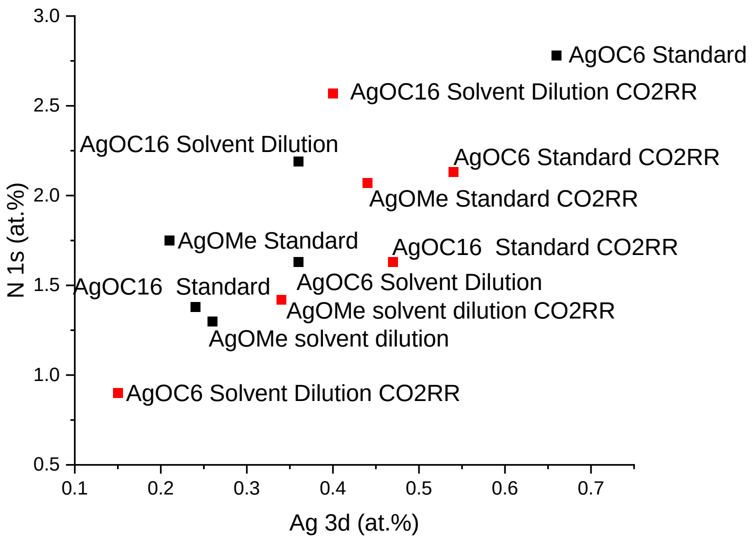

Figure S 12: Atomic weight percentages calculated from the Silver Ag5d and N1s transitions in the recorded survey scans for GDE before and after electrolysis in black and red respectively.

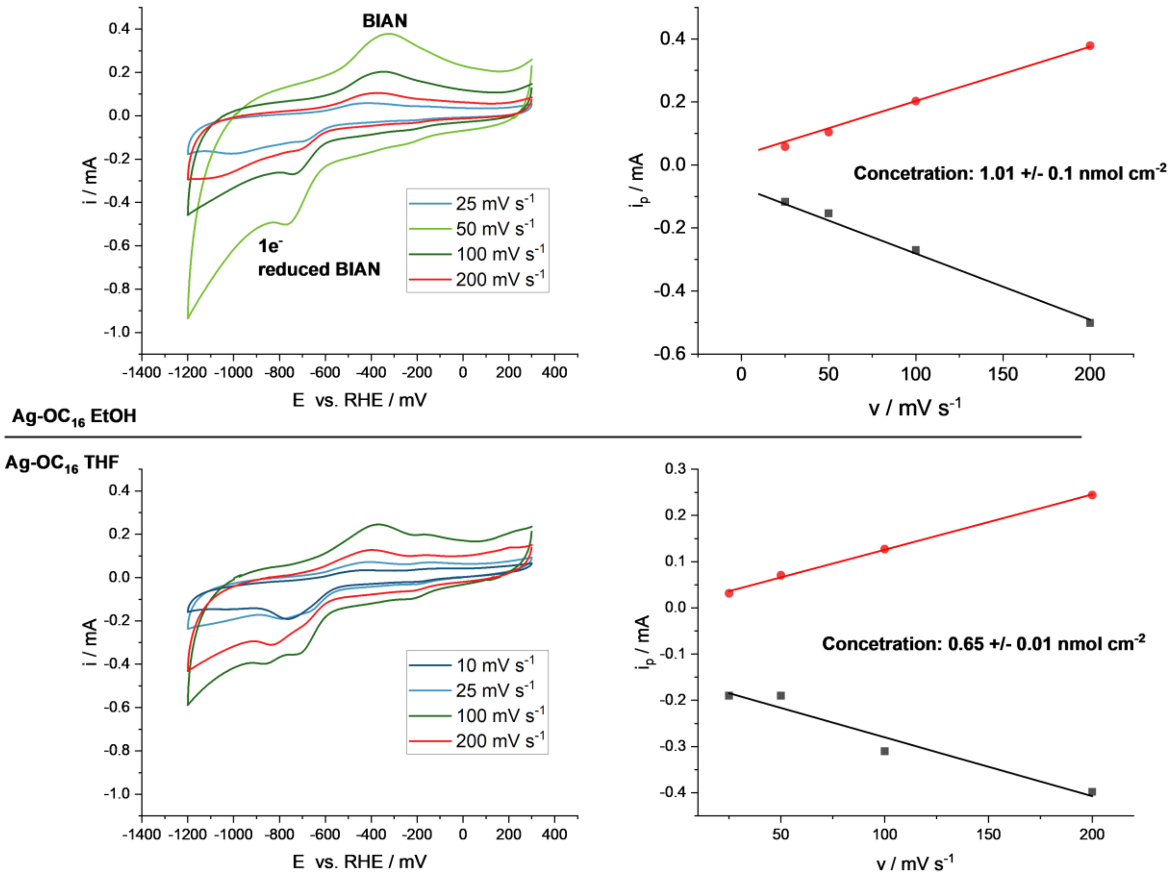

Figure S 13: Quantification of the electro-active species via cyclovoltammetry at different scan rates in 0.1 M $KHCO_3$ for the Ag-OC_{16} Ethanol and THF-generated GDEs.

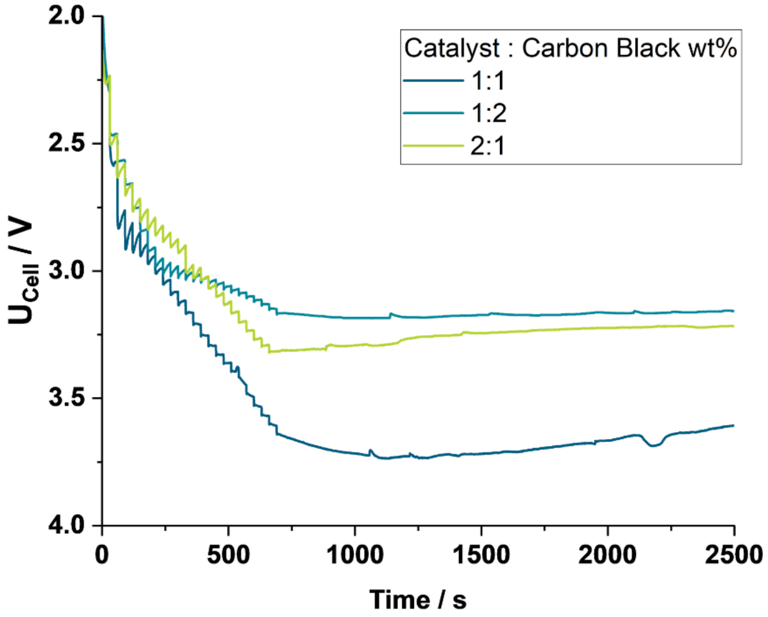

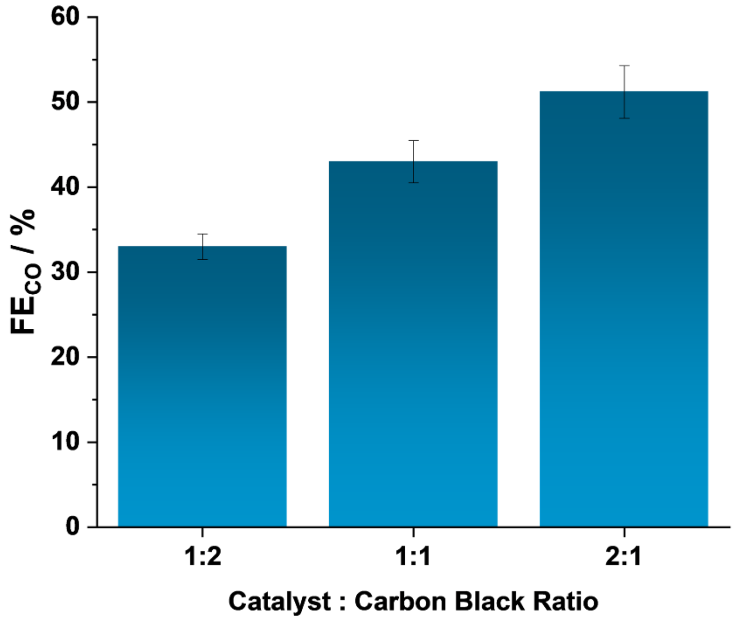

Figure S 14: Influence of the Ag-OC_{16} – Ensaco 250G ratio in the optimized THF-based ink at 600 mA cm^{-2} in terms of FE_{CO} and cell voltage.

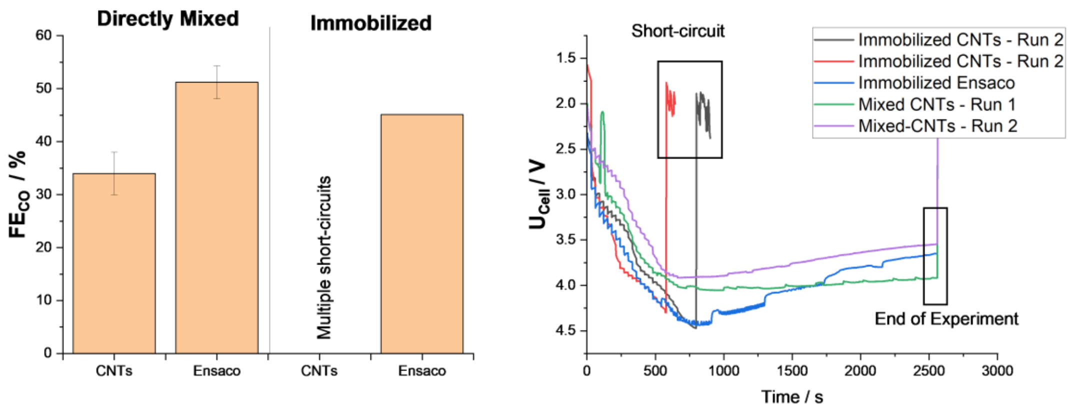

Figure S 15: Comparison of the influence of direct mixing vs. prior inmmobilizatio of Ag-OC$_{16}$ on the carbon support THF-based ink at 600 mA cm^{-2} in terms of FE$_{CO}$ and cell voltage.

Table S2 Comparison of the achieved partial current density for CO and associated mass activity of this report with molecular and Ag-based GDEs

Catalyst	j_{CO} / mA cm^{-2}	Mass Activity / mA mg^{-1}	Reference
Ag-NPs	600	600	[4]
Ag/TiO_2	125	2500	[5]
CoPc	50	5882	[6]
Co-CNTs-MW	191	1060	[7]
Ag/C_{Ketjen}-PTFE	270	5400	[8]
Ag/C_{Vuzlca}-PiperION	360	12300	[9]
Ag-OC_{16}	307	25495	This work
Ag-SC_6	402	24736	This work

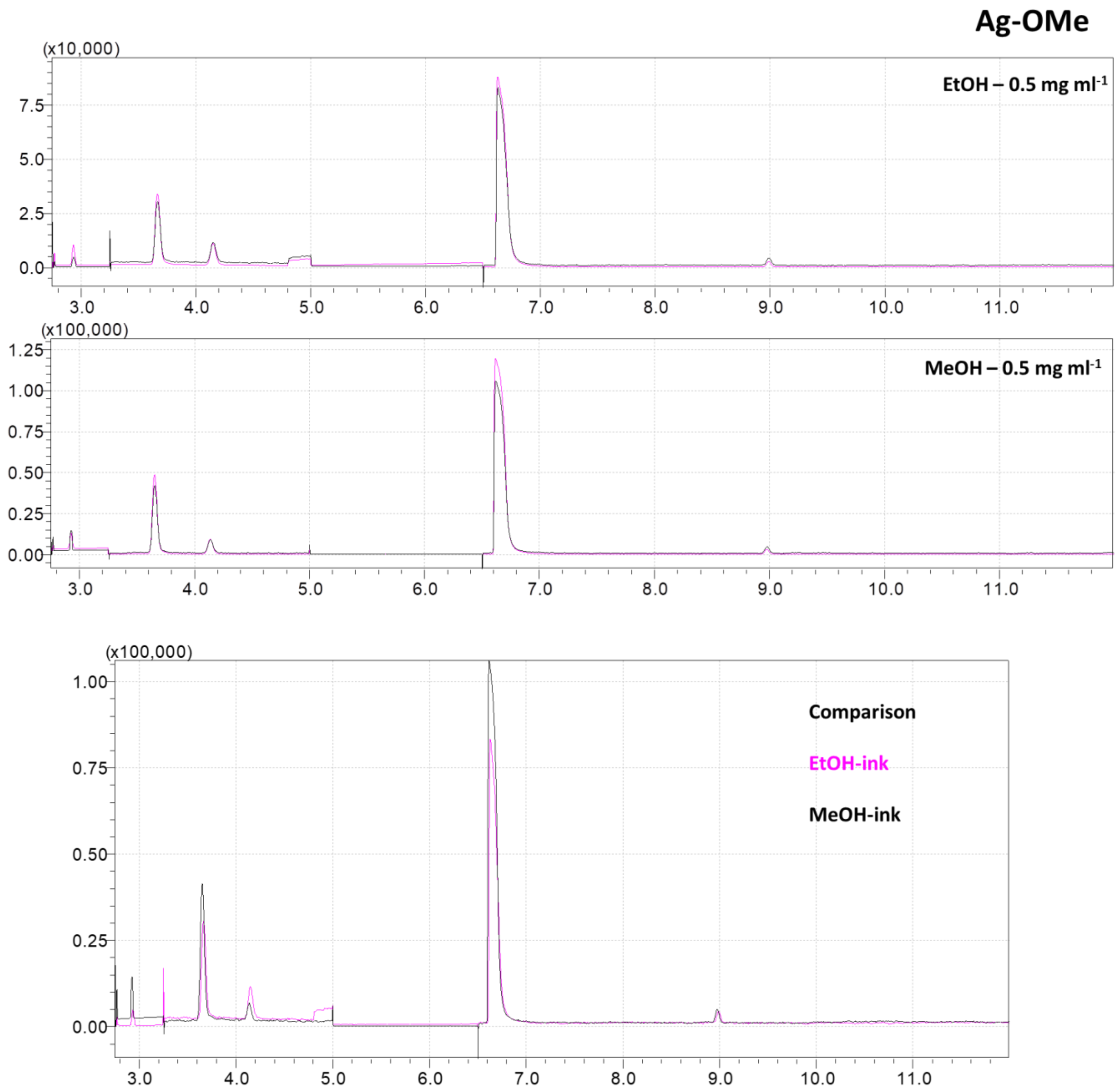

Figure S 16: Exemplary GC-traces for the Ag-OMe tested GDEs.

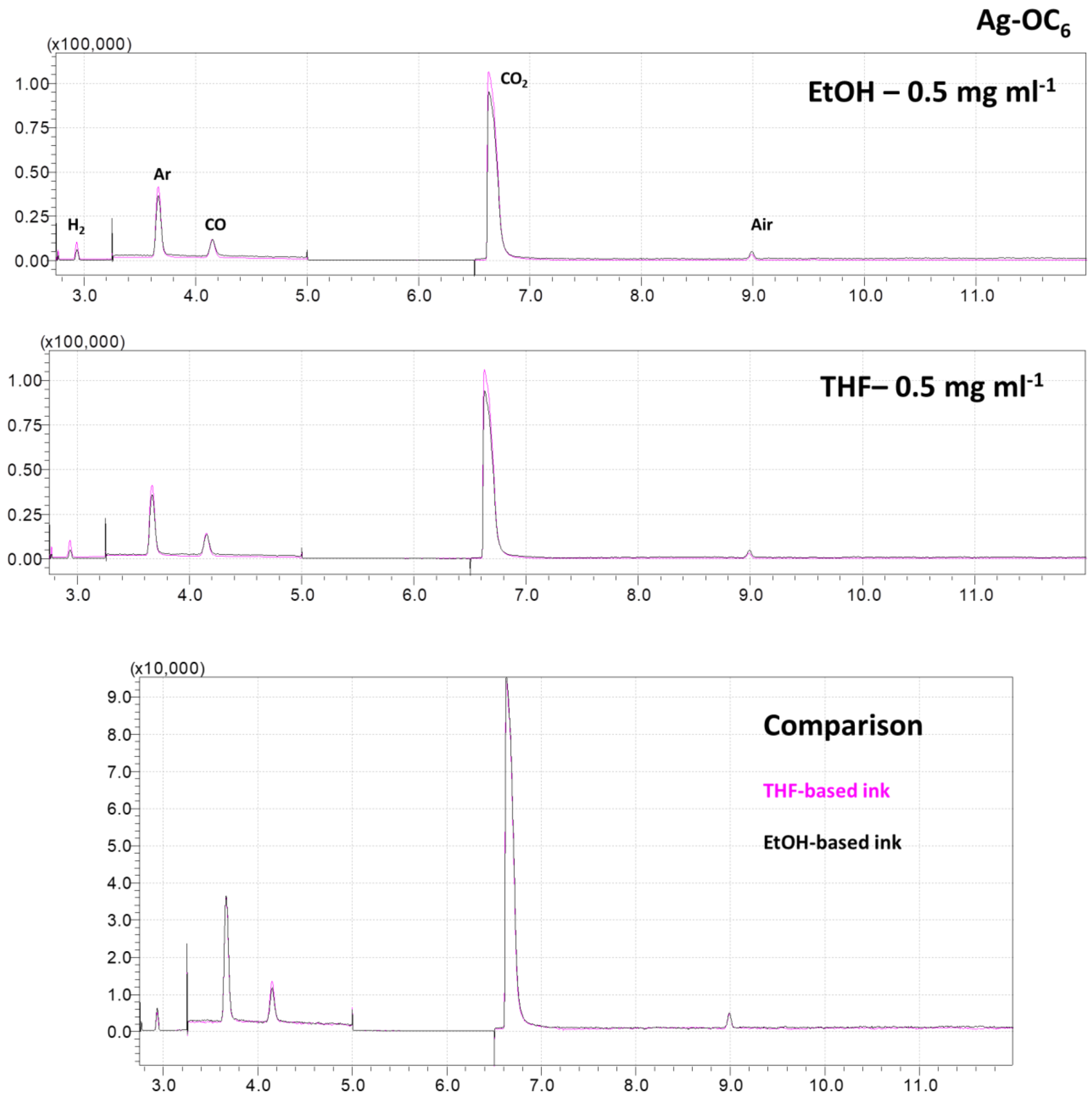

Figure S 17: Exemplary GC-traces for the Ag-OC_6 tested GDEs.

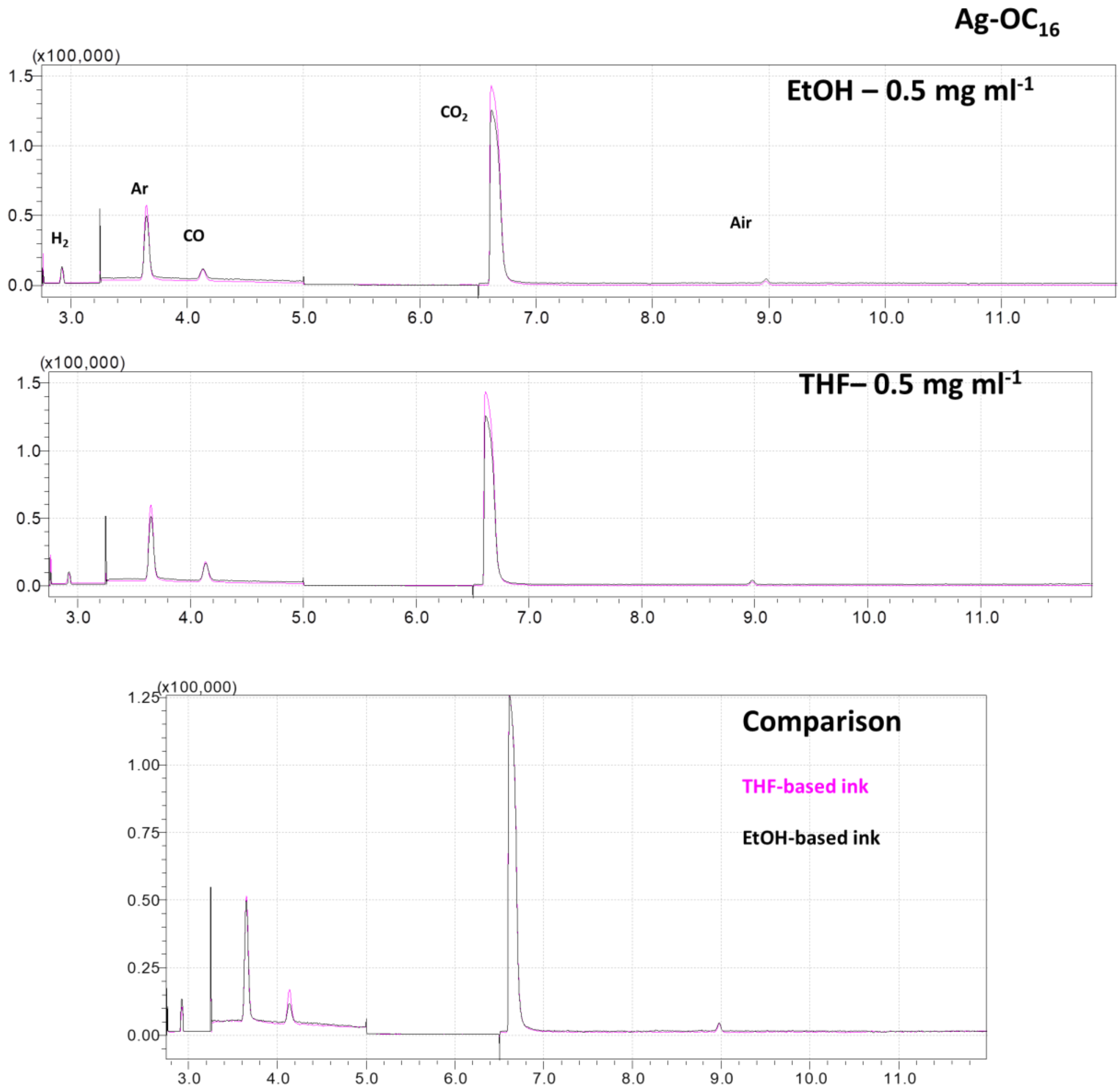

Figure S 18: Exemplary GC-traces for the Ag-OC_{16} tested GDEs.

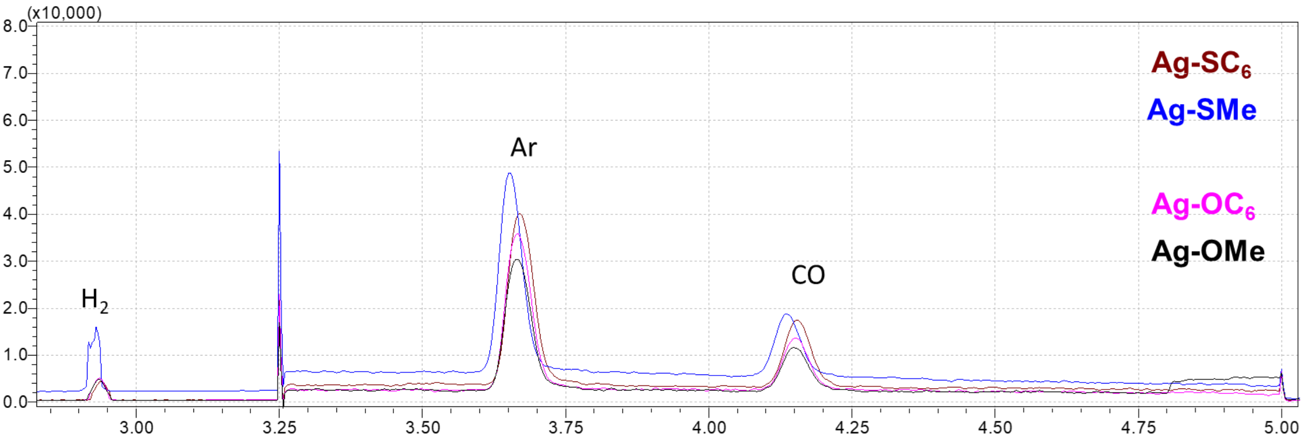

Figure S 19: Comparison of the GC-traces between oxygen and sulfur linked Ag-BIANs.

Characterization Data

SC6-Aniline. 4-*n*-hexylthioaniline

^{1}H NMR (300 MHz, $CDCl_3$, 25 °C) δ / ppm: 0.87 (t, J = 6.8 Hz, 3 H, CH_3), 1.21 – 1.42 (m, 6 H, CH_2), 1.56 (p, J = 7.4 Hz, 2 H, CH_2), 2.76 (t, J = 6.8 Hz, 2 H, CH_2), 3.67 (bs, 2 H, NH_2), 6.62 (dt, J_1 = 8.6 Hz, J_2 = 2.8 Hz, 2 H, ArH), 7.23 (dt, J_1 = 8.6 Hz, J_2 = 2.8 Hz, 2 H, ArH)

^{13}C NMR (126 MHz, $CDCl_3$, 25 °C) δ / ppm: 14.2, 22.7, 28.5, 29.5, 31.5, 36.5, 115.7, 124.0, 133.8, 145.8

HRMS (ESI) *m*/*z* calcd. for $C_{36}H_{41}N_2S_2^+$: 210.1316 [*M*-H]$^+$; found: 210.1314

OMe-BIAN. *N^1*,*N^2*-bis(4-methoxyphenyl)acenaphthylene-1,2-diimine (4-OMe-BIAN) was synthesized according to the general procedure for the preparation of BIAN ligands employing *p*-anisidine with 15 min reaction time and obtained as red solid in 63% yield.

^{1}H NMR (300 MHz, $CDCl_3$, 25 °C) δ / ppm: 3.90 (s, 6 H, CH_3), 7.00 – 7.04 (m, 6 H, ArH), 7.08 – 7.11 (m, 4 H, ArH), 7.39 (dd, J_1 = 7.3 Hz, J_2 = 8.2 Hz, 2 H, ArH), 7.89 (d, J = 8.2 Hz, 2 H, ArH)

^{13}C NMR (75 MHz, $CDCl_3$, 25 °C) δ / ppm: 55.6, 114.8, 119.9, 123.8, 127.7, 128.9, 129.0, 131.4, 145.1, 157.0, 161.7

UV-vis (ACN) λ_{max} nm (log ε): 212 (4.64), 229 (4.83), 291 (4.08), 424 (3.63)

UV-vis (DCM) λ_{max} nm (log ε): 232 (4.95), 293 (4.19), 431 (3.72)

OC6-BIAN. *N^1*,*N^2*-bis(4-hexyloxyphenyl)acenaphthylene-1,2-diimine (4-*n*-hexyloxy-BIAN) was synthesized according to the general procedure for the preparation of BIAN ligands employing 4-*n*-hexyloxyaniline with 20 min reaction time and obtained as dark red solid in 72% yield.

^{1}H NMR (500 MHz, $CDCl_3$, 25 °C) δ / ppm: 0.92 – 0.95 (m, 6 H, CH_3), 1.36 – 1.41 (m, 8 H, CH_2), 1.49 – 1.55 (m, 4 H, CH_2), 1.81 – 1.87 (m, 4 H, CH_2), 4.03 (t, J = 6.6 Hz, 4 H, CH_2), 6.99 – 7.02 (m, 4 H, ArH), 7.04 – 7.09 (m, 6 H, ArH), 7.39 (t, J = 7.8 Hz, 2 H, ArH), 7.88 (d, J = 8.2 Hz, 2 H, ArH)

^{13}C NMR (126 MHz, $CDCl_3$, 25 °C) δ / ppm: 14.2, 22.8, 26.0, 29.5, 31.8, 68.5, 115.3, 119.9, 123.8, 127.7, 128.9, 128.9, 131.4, 141.8, 144.9, 156.6, 161.7

UV-vis (ACN) λ_{max} nm (log ε): 230 (5.09), 290 (4.44), 422 (3.92)

UV-vis (DCM) λ_{max} nm (log ε): 232 (4.96), 292 (4.20), 434 (3.75)

HRMS (ESI) *m*/*z* calcd for $C_{36}H_{40}N_2O_2$+H$^+$: 533.3163 [*M*+H]$^+$; found: 533.3160

OC16-BIAN. *N*[1],*N*[2]-bis(4-hexadecyloxyphenyl)acenaphthylene-1,2-diimine (4-*n*-hexadecyloxy-BIAN) was synthesized according to the general procedure for the preparation of BIAN ligands employing 4-*n*-hexadecyloxyaniline with 20 min reaction time and obtained as pale red solid in 64% yield.

^{1}H NMR (500 MHz, $CDCl_3$, 25 °C) δ / ppm: 0.88 (t, *J* = 6.9 Hz, 6 H, CH_3), 1.26 – 1.39 (m, 48 H, CH_2), 1.51 (p, *J* = 7.5 Hz, 4 H, CH_2), 1.84 (p, *J* = 7.5 Hz, 4 H, CH_2), 4.03 (t, *J* = 6.6 Hz, 4 H, CH_2), 7.00 (d, *J* = 8.6 Hz, 4 H, ArH), 7.05 (d, *J* = 7.3 Hz, 2 H, ArH), 7.08 (d, *J* = 8.6 Hz, 4 H, ArH), 7.39 (t, *J* = 7.8 Hz, 2 H, ArH), 7.88 (d, *J* = 8.3 Hz, 2 H, ArH)

^{13}C NMR (126 MHz, $CDCl_3$, 25 °C) δ / ppm: 14.3, 22.8, 26.3, 29.5, 29.6, 29.6, 29.8, 29.8, 32.1, 68.5, 115.3, 119.9, 123.8, 127.7, 128.9, 131.4, 141.8, 144.9, 156.6, 161.7

UV-vis (DCM) λ_{max} nm (log *ε*): 232 (5.03), 296 (4.32), 433 (3.90)

HRMS (ESI) *m*/*z* calcd for $C_{56}H_{80}N_2O_2$+H$^+$: 813.6293 [*M*+H]$^+$; found: 813.6292

SMe-BIAN. *N*[1],*N*[2]-bis(4-methylthiophenyl)acenaphthylene-1,2-diimine (4-SMe-BIAN) was synthesized according to the general procedure for the preparation of BIAN ligands employing 4-methylthioaniline with 15 min reaction time and obtained as dark red solid in 74% yield.

^{1}H NMR (500 MHz, $CDCl_3$, 25 °C) δ / ppm: 2.56 (s, 6 H, CH_3), 7.01 (d, *J* = 7.3 Hz, 2 H, ArH), 7.09 (d, *J* = 8.5 Hz, 4 H, ArH), 7.37 – 7.42 (m, 6 H, ArH), 7.90 (d, *J* = 8.2 Hz, 2 H, ArH)

^{13}C NMR (126 MHz, $CDCl_3$, 25 °C) δ / ppm: 16.7, 119.3, 124.1, 127.8, 128.2, 128.6, 129.2, 131.4, 134.0, 141.9, 149.2, 161.6

UV-vis (DCM) λ_{max} nm (log *ε*): 230 (4.82), 261 (4.59), 300 (4.09), 448 (3.58)

HRMS (ESI) *m*/*z* calcd. for $C_{26}H_{20}N_2S_2$+H$^+$: 425.1146 [*M*+H]$^+$; found: 425.1147

SC6-BIAN. *N*[1],*N*[2]-bis(4-hexylthiophenyl)acenaphthylene-1,2-diimine (4-*n*-hexylthio-BIAN) was synthesized according to the general procedure for the preparation of BIAN ligands employing 4-*n*-hexylthioaniline with 20 min reaction time and obtained as orange solid in 58% yield.

^{1}H NMR (300 MHz, $CDCl_3$, 25 °C) δ / ppm: 0.93 (t, *J* = 6.9 Hz, 6 H, CH_3), 1.28 – 1.42 (m, 8 H, CH_2), 1.50 (p, *J* = 7.4 Hz, 4 H, CH_2), 1.74 (p, *J* = 7.6 Hz, 4 H, CH_2), 3.01 (t, *J* = 7.4 Hz, 4 H, CH_2), 7.01 (d, *J* = 7.2 Hz, 2 H, ArH), 7.10 (dt, J_1 = 8.5 Hz, J_2 = 1.8 Hz, 4 H, ArH), 7.40 – 7.50 (m, 6 H, ArH), 7.94 (d, *J* = 8.2 Hz, 2 H, ArH)

^{13}C NMR (126 MHz, $CDCl_3$, 25 °C) δ / ppm: 14.2, 22.7, 28.7, 29.3, 31.6, 34.6, 119.2, 124.1, 127.8, 128.6, 129.3, 130.9, 131.4, 132.5, 141.9, 149.9, 161.5

UV-vis (DCM) λ_{max} nm (log ε): 232 (4.79), 261 (4.56), 302 (4.02), 432 (3.63)

HRMS (ESI) *m*/*z* calcd. for $C_{36}H_{41}N_2S_2^+$: 565.2711 [*M*-H]$^+$; found: 565.2709

Ag-OMe. [Ag(4-OMe-BIAN)$_2$]BF_4 was synthesized according to the general procedure for the preparation of [Ag(I)(BIAN)$_2$]BF_4 complexes employing [Ag(ACN)$_4$]BF_4 with 2 equivalents of **OMe-BIAN** and obtained as red solid in 93% yield.

^{1}H NMR (500 MHz, $CDCl_3$, 25 °C) δ / ppm: 3.90 (s, 12 H, CH_3), 7.04 (d, *J* = 8.9 Hz, 8 H, ArH), 7.18 (d, *J* = 8.9 Hz, 8 H, ArH), 7.27 (d, overlapping, *J* = 8.0 Hz, 4 H, ArH) 7.50 (t, *J* = 8.0 Hz, 8 H, ArH), 8.03 (d, *J* = 8.2 Hz, 4 H, ArH)

^{13}C NMR (126 MHz, $CDCl_3$, 25 °C) δ / ppm: 55.9, 115.2, 121.6, 125.0, 127.2, 128.3, 131.0, 131.2, 141.8, 142.5, 158.7, 161.4

UV-vis (ACN) λ_{max} nm (log ε): 212 (4.92), 229 (5.12), 292 (4.36), 422 (3.88)

UV-vis (DCM) λ_{max} nm (log ε): 231 (5.25), 309 (4.51), 396 (4.13)

HRMS (ESI) *m*/*z* calcd for $C_{52}H_{40}AgN_4O_4^+$: 891.2100 [*M*-BF_4]$^+$; found: 891.2096

Ag-OC6. [Ag(4-*n*-hexyloxy-BIAN)$_2$]BF_4 was synthesized according to the general procedure for the preparation of [Ag(I)(BIAN)$_2$]BF_4 complexes employing [Ag(ACN)$_4$]BF_4 with 2 equivalents of **OC6-BIAN** and obtained as dark red solid in 96% yield.

^{1}H NMR (500 MHz, $CDCl_3$, 25 °C) δ / ppm: 0.94 – 0.95 (m, 12 H, CH_3), 1.37 – 1.38 (m, 16 H, CH_2), 1.48 – 1.54 (m, 8 H, CH_2), 1.80 – 1.85 (m, 8 H, CH_2), 4.02 (t, *J* = 6.4 Hz, 8 H, CH_2), 7.01 (d, *J* = 8.4 Hz, 8 H, ArH), 7.13 (d, *J* = 8.4 Hz, 8 H, ArH), 7.27 (d, overlapping, *J* = 7.5 Hz, 4 H, ArH), 7.50 (t, *J* = 7.8 Hz, 4 H, ArH), 8.04 (d, *J* = 8.2 Hz, 4 H, ArH)

^{13}C NMR (126 MHz, $CDCl_3$, 25 °C) δ / ppm: 14.2, 22.8, 25.9, 29.4, 31.8, 68.7, 115.3, 121.3, 125.0, 127.1, 128.3, 131.1, 131.2, 141.7, 142.5, 158.4, 161.4

UV-vis (ACN) λ_{max} nm (log ε): 230 (5.25), 290 (4.62), 420 (4.09)

UV-vis (DCM) λ_{max} nm (log ε): 231 (5.21), 308 (4.45), 405 (4.08)

HRMS (ESI) *m*/*z* calcd for $C_{72}H_{80}AgN_4O_4^+$: 1172.5303 [*M*-BF_4]$^+$; found: 1172.5306

Ag-OC16. [Ag(4-*n*-hexadecyloxy-BIAN)$_2$]BF_4 was synthesized according to the general procedure for the preparation of [Ag(I)(BIAN)$_2$]BF_4 complexes employing [Ag(ACN)$_4$]BF_4 with 2 equivalents of **OC16-BIAN** and obtained as pale red solid in 99% yield.

^{1}H NMR (500 MHz, $CDCl_3$, 25 °C) δ / ppm: 0.87 (t, *J* = 6.9 Hz, 12 H, CH_3), 1.26 – 1.39 (m, 96 H, CH_2), 1.50 (p, *J* = 7.5 Hz, 8 H, CH_2), 1.83 (p, *J* = 7.5 Hz, 8 H, CH_2), 4.02 (t, *J* = 6.5 Hz, 8 H, CH_2), 6.99 (d, *J* = 8.6 Hz, 8 H, ArH), 7.10 (d, *J* = 8.5 Hz, 8 H, ArH), 7.28 (d, *J* = 7.4 Hz, 4 H, ArH), 7.51 (t, *J* = 7.8 Hz, 4 H, ArH), 8.04 (d, *J* = 8.3 Hz, 4 H, ArH)

^{13}C NMR (126 MHz, $CDCl_3$, 25 °C) δ / ppm: 14.3, 22.8, 26.3, 29.5, 29.7, 29.8, 29.9, 32.1, 68.7, 115.4, 121.3, 125.0, 127.2, 128.3, 131.0, 131.2, 141.7, 142.5, 158.3, 161.4

UV-vis (DCM) λ_{max} nm (log ε): 231 (5.23), 310 (4.56), 403 (4.18)

HRMS (ESI) *m*/*z* calcd for $C_{112}H_{160}AgN_4O_4^+$: 1732.1491 [*M*-BF_4]$^+$; found: 1732.1492

Ag-SMe. [Ag(4-SMe-BIAN)$_2$]BF_4 was synthesized according to the general procedure for the preparation of [Ag(I)(BIAN)$_2$]BF_4 complexes employing [Ag(ACN)$_4$]BF_4 with 2 equivalents of **SMe-BIAN** and obtained as red solid in 78% yield.

^1H NMR (500 MHz, CD_3CN, 25 °C) δ / ppm: 2.50 (s, 12 H, CH_3), 6.91 (d, *J* = 8.6 Hz, 8 H, ArH), 7.11 (d, *J* = 7.3 Hz, 4 H, ArH), 7.20 (d, *J* = 8.6 Hz, 8 H, ArH), 7.52 (t, *J* = 7.7 Hz, 4 H, ArH), 8.11 (d, *J* = 8.2 Hz, 4 H, ArH)

^{13}C NMR (126 MHz, CD_3CN, 25 °C) δ / ppm: 15.3, 120.2, 124.6, 127.6, 128.3, 130.7, 131.3, 136.3, 142.0, 146.6, 160.8

UV-vis (ACN) λ_{max} nm (log ε): 203 (5.11), 229 (5.14), 259 (4.99), 300 (4.43), 429 (3.98)

HRMS (ESI) *m*/*z* calcd. for $C_{52}H_{40}AgN_4S_4^+$: 955.1187 [*M*-BF_4]$^+$; found: 955.1185

Ag-SC6. [Ag(4-*n*-hexylthio-BIAN)$_2$]BF_4 was synthesized according to the general procedure for the preparation of [Ag(I)(BIAN)$_2$]BF_4 complexes employing [Ag(ACN)$_4$]BF_4 with 2 equivalents of **SC6-BIAN** and obtained as dark red solid in 97% yield.

^1H NMR (500 MHz, $CDCl_3$, 25 °C) δ / ppm: 0.89 (t, *J* = 6.9 Hz, 12 H, CH_3), 1.30 – 1.34 (m, 16 H, CH_2), 1.48 (p, *J* = 7.4 Hz, 8 H, CH_2), 1.71 (p, *J* = 7.5 Hz, 8 H, CH_2), 3.03 (t, *J* = 7.4 Hz, 8 H, CH_2), 7.01 (d, *J* = 8.3 Hz, 8 H, ArH), 7.10 (d, *J* = 7.3 Hz, 4 H, ArH), 7.35 (d, *J* = 8.4 Hz, 8 H, ArH), 7.46 (t, *J* = 7.8 Hz, 4 H, ArH), 8.03 (d, *J* = 8.3 Hz, 2 H, ArH)

^{13}C NMR (126 MHz, $CDCl_3$, 25 °C) δ / ppm: 14.2, 22.7, 28.7, 29.1, 31.6, 34.2, 120.4, 125.2, 127.2, 128.3, 130.0, 131.0, 131.2, 135.0, 142.6, 147.2, 161.6

UV-vis (DCM) λ_{max} nm (log ε): 233 (5.14), 266 (5.03), 441 (4.10)

HRMS (ESI) *m*/*z* calcd. for $C_{72}H_{80}N_4S_4Ag^+$: 1235.4316 [*M*-BF_4]$^+$; found: 1235.4315

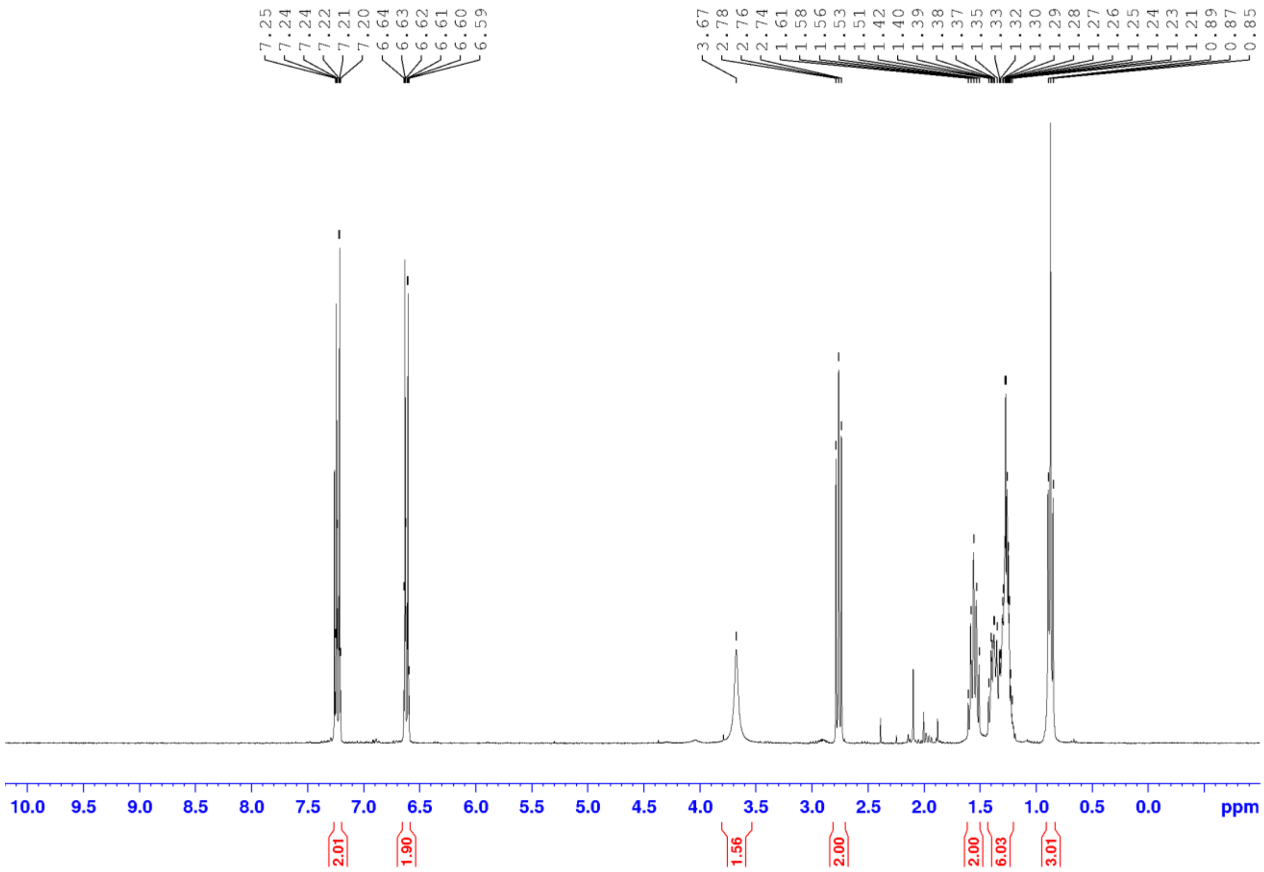

Figure S 20: ^{1}H NMR (300 MHz, $CDCl_3$, 25 °C) of 4-(*n*-hexylthio)aniline **SC6-Aniline**.

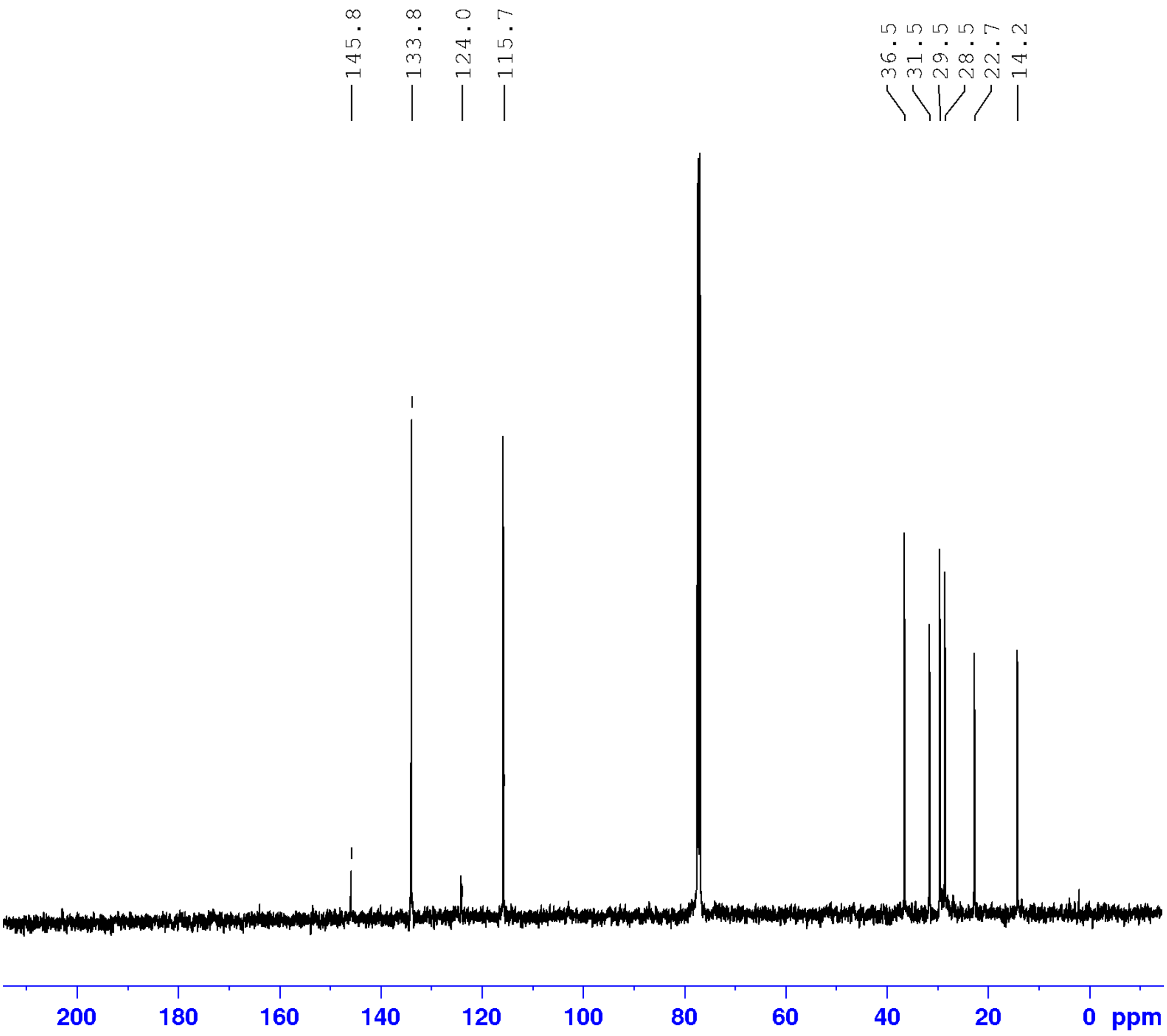

Figure S 21: ^{13}C NMR (126 MHz, $CDCl_3$, 25 °C) of 4-(*n*-hexylthio)aniline **SC6-Aniline**.

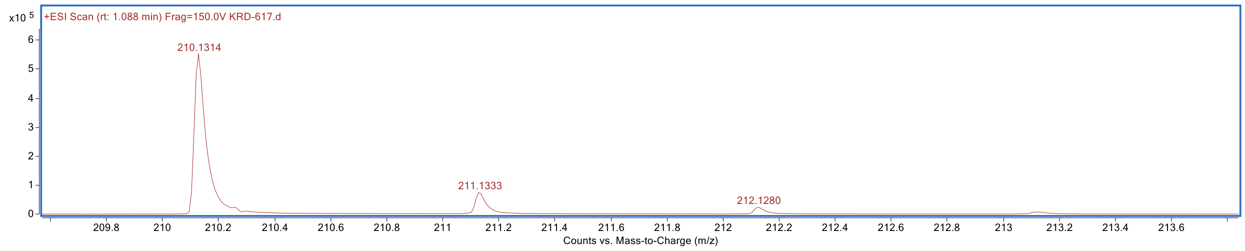

Figure S 22: HRMS spectrum of 4-(*n*-hexylthio)aniline **SC6-Aniline**.

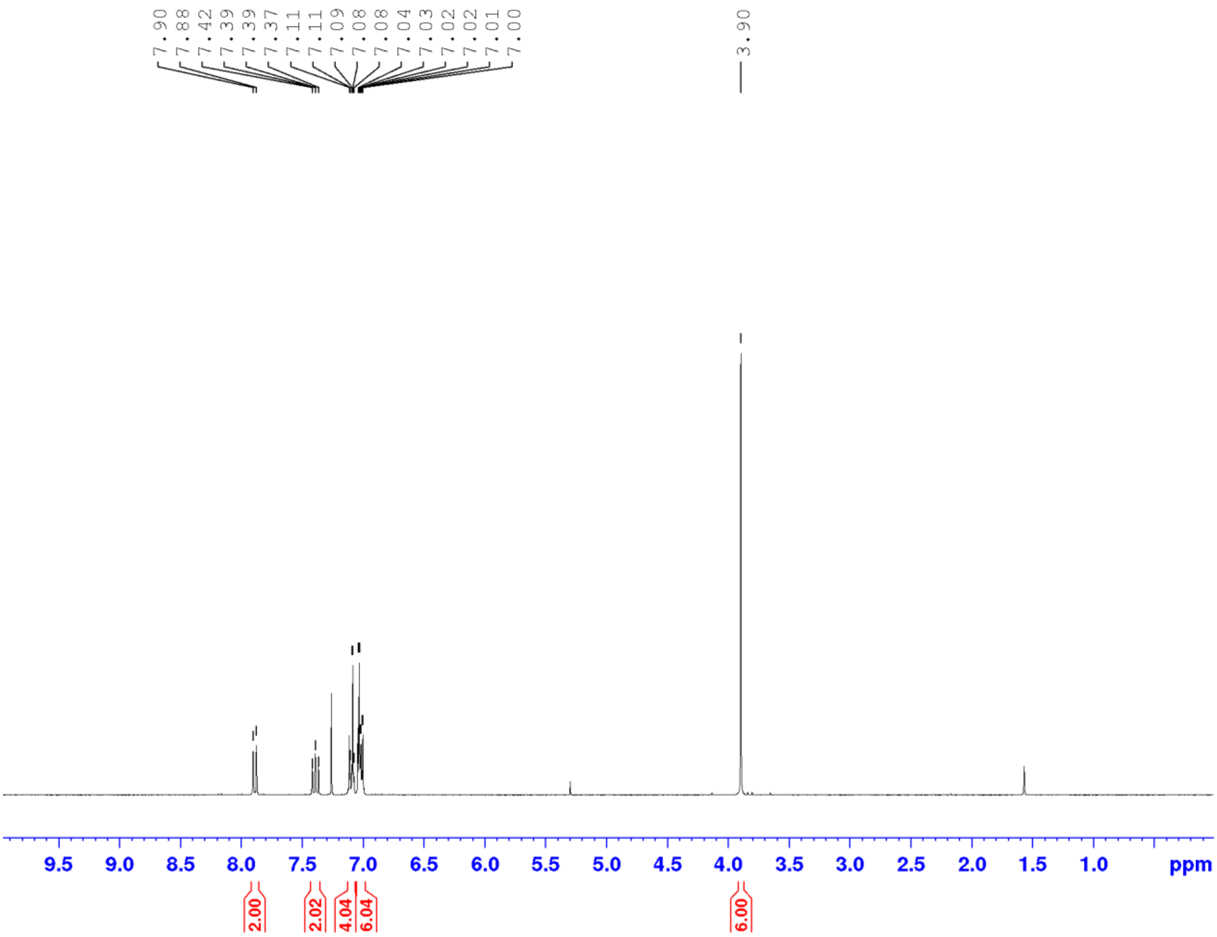

Figure S 23: ^{1}H NMR (300 MHz, $CDCl_3$, 25 °C) of *N*[1],*N*[2]-bis(4-methoxyphenyl)acenaphthylene-1,2-diimine **OMe-BIAN**.

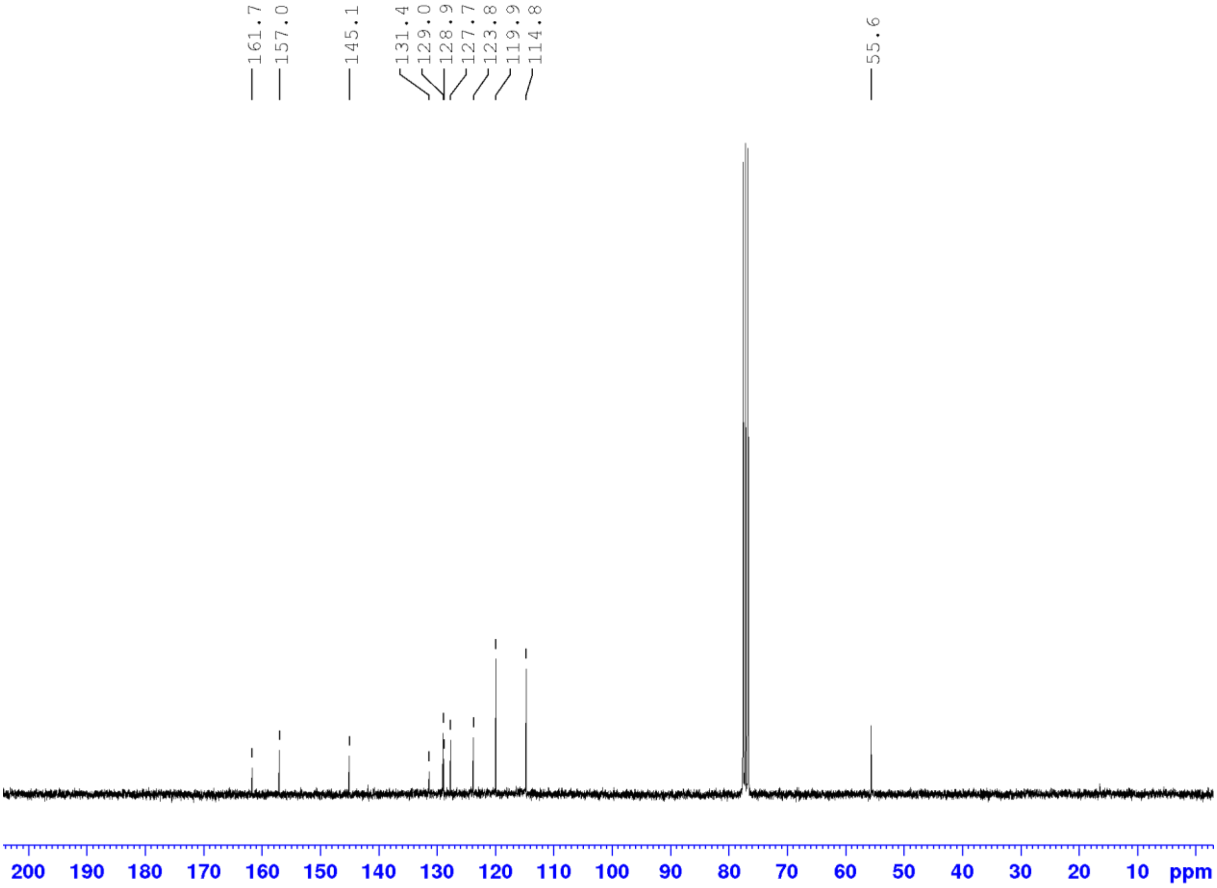

Figure S 24: ^{13}C NMR (75 MHz, $CDCl_3$, 25 °C) of N^1,N^2-bis(4-methoxyphenyl)acenaphthylene-1,2-diimine **OMe-BIAN**.

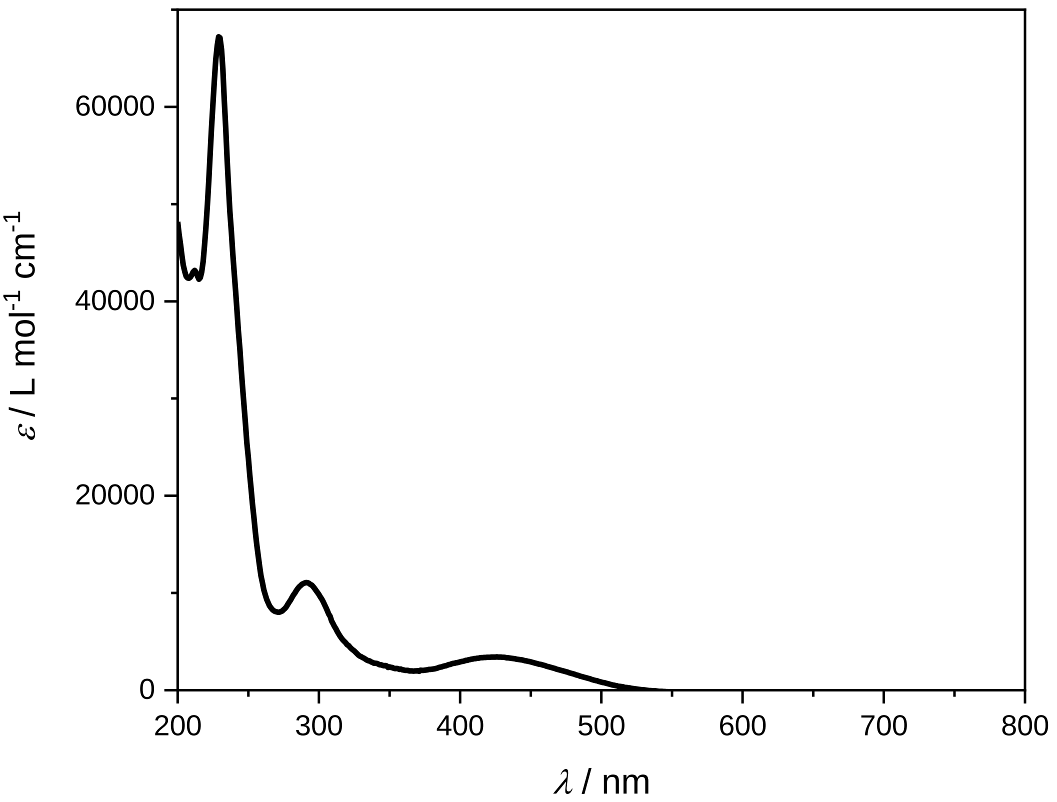

Figure S 25: UV-vis absorption spectrum of *N*[1],*N*[2]-bis(4-methoxyphenyl)acenaphthylene-1,2-diimine **OMe-BIAN** in ACN.

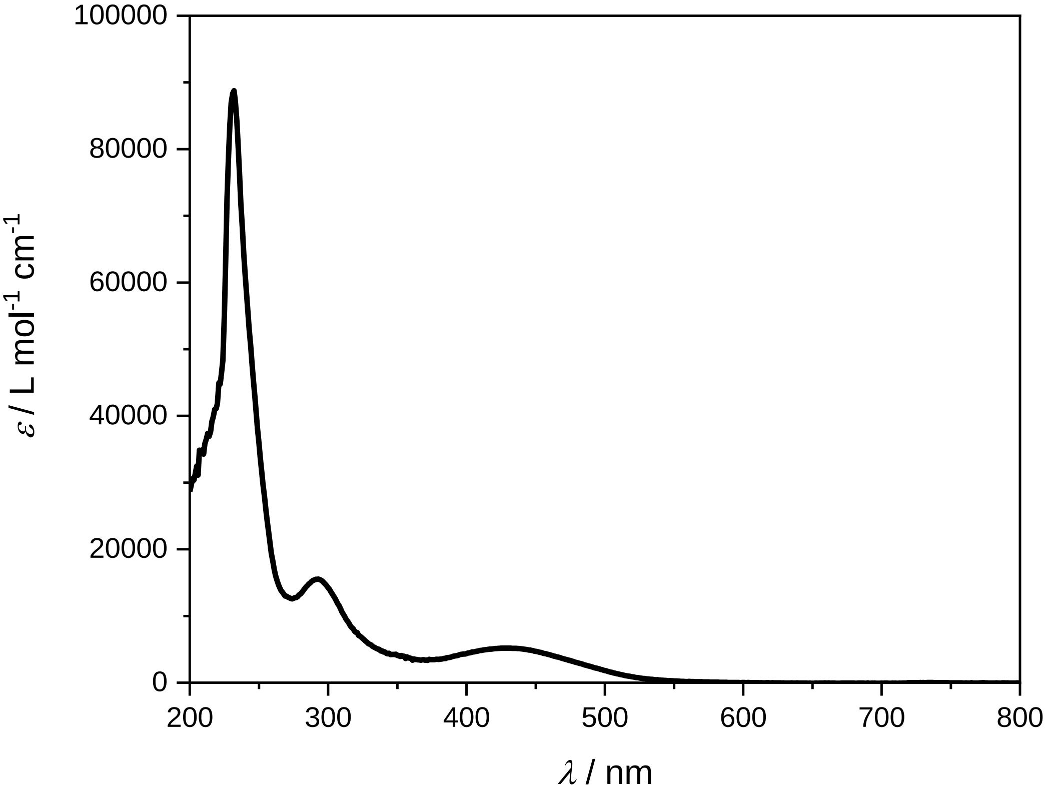

Figure S 26: UV-vis absorption spectrum of *N*[1],*N*[2]-bis(4-*n*-methoxyphenyl)acenaphthylene-1,2-diimine **OMe-BIAN** in DCM.

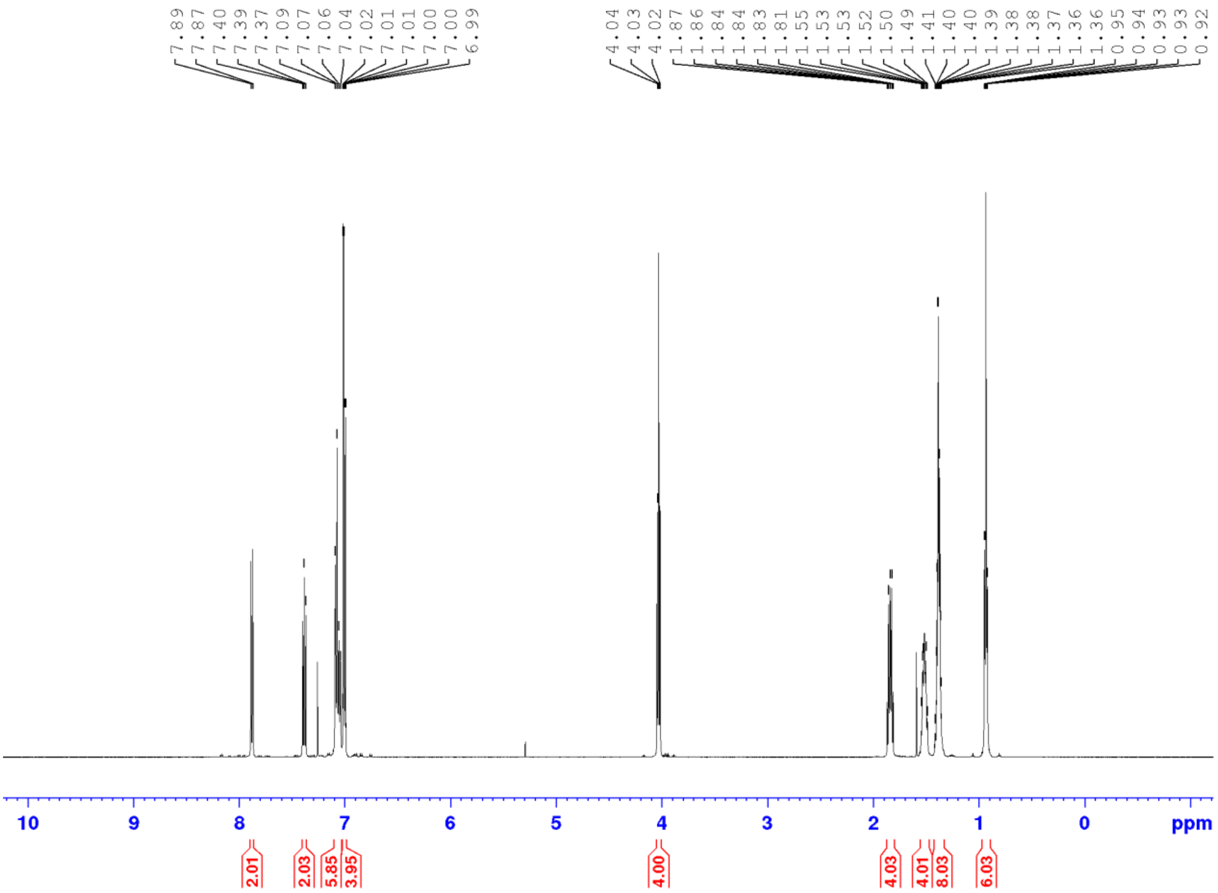

Figure S 27: ^{1}H NMR (500 MHz, $CDCl_3$, 25 °C) of *N¹*,*N²*-bis(4-*n*-hexyloxyphenyl)acenaphthylene-1,2-diimine **OC6-BIAN**.

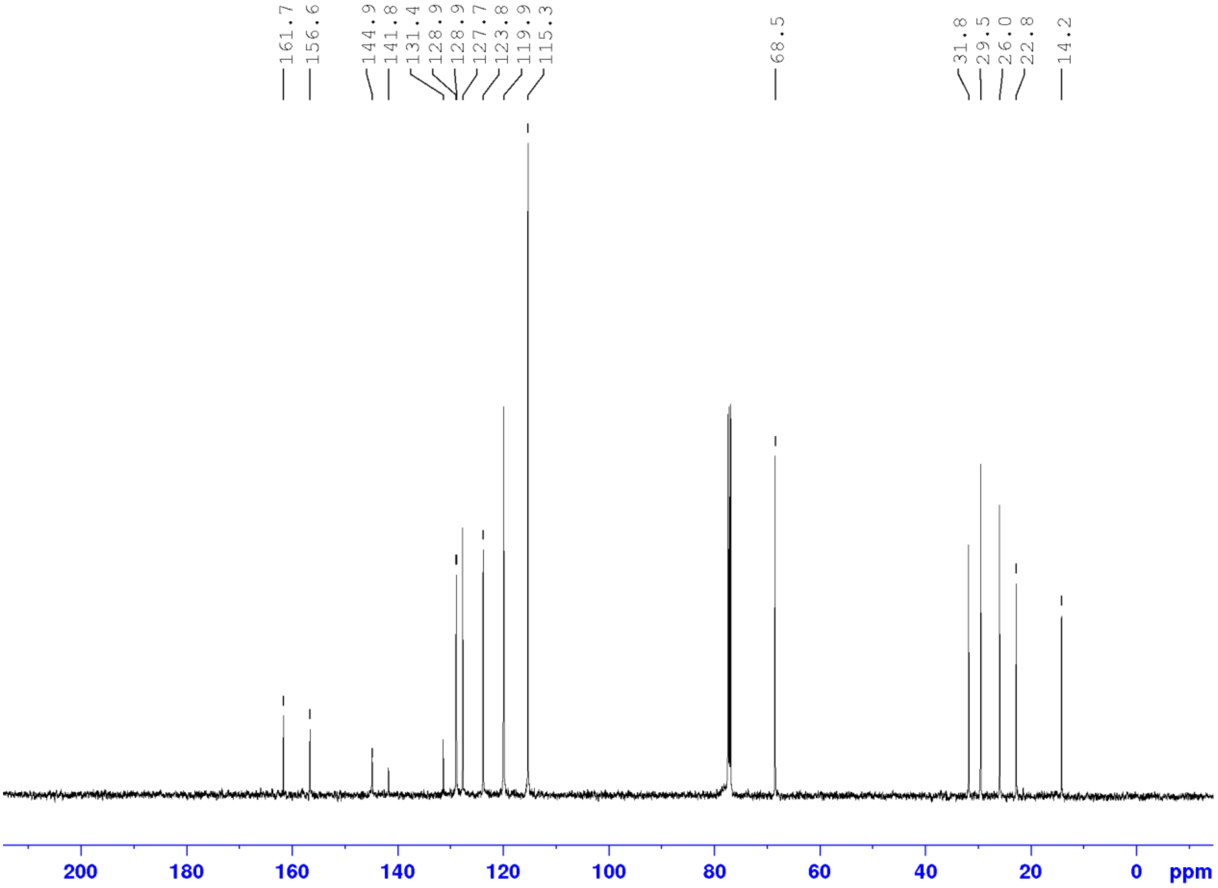

Figure S 28: ^{13}C NMR (126 MHz, $CDCl_3$, 25 °C) of *N*^{1},*N*^{2}-bis(4-*n*-hexyloxyphenyl)acenaphthylene-1,2-diimine **OC6-BIAN**.

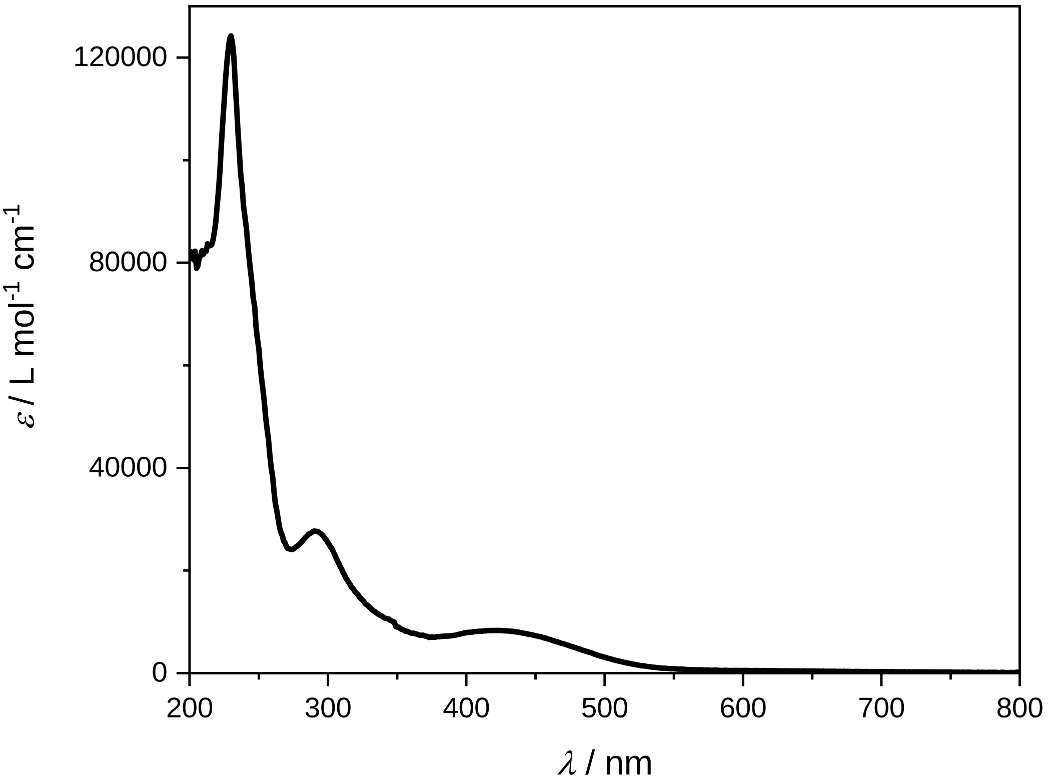

Figure S 29: UV-vis absorption spectrum of N^1,N^2-bis(4-*n*-hexyloxyphenyl)acenaphthylene-1,2-diimine **OC6-BIAN** in ACN.

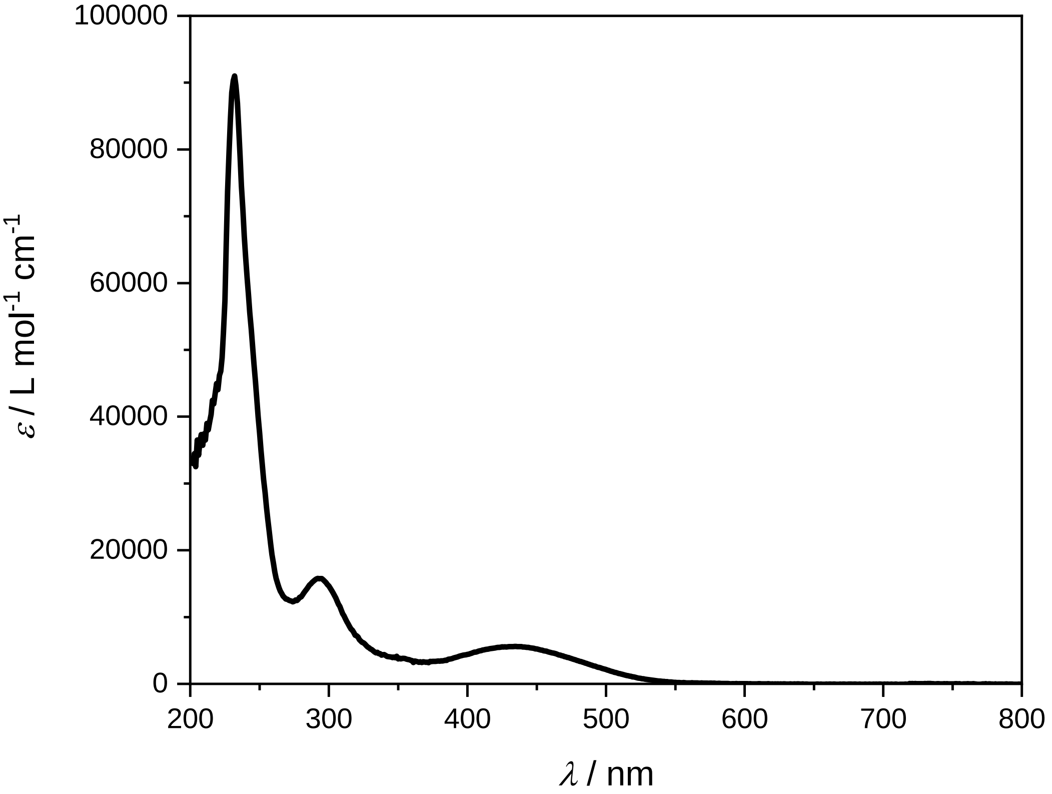

Figure S 30: UV-vis absorption spectrum of N^1,N^2-bis(4-*n*-hexyloxyphenyl)acenaphthylene-1,2-diimine **OC6-BIAN** in DCM.

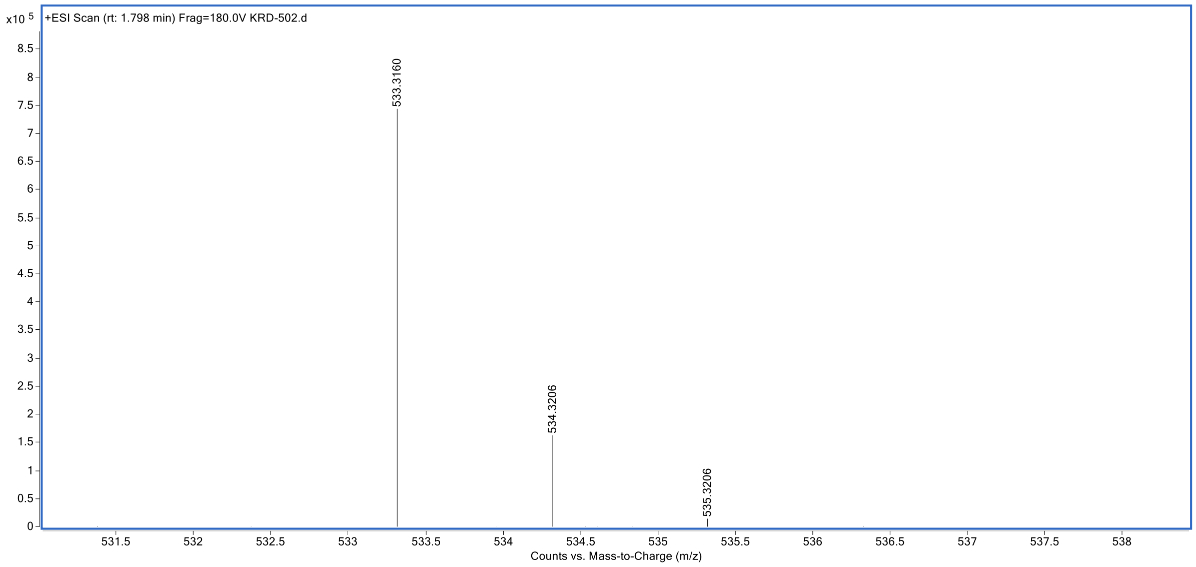

Figure S 31: HRMS spectrum of N^1,N^2-bis(4-*n*-hexyloxyphenyl)acenaphthylene-1,2-diimine **OC6-BIAN**.

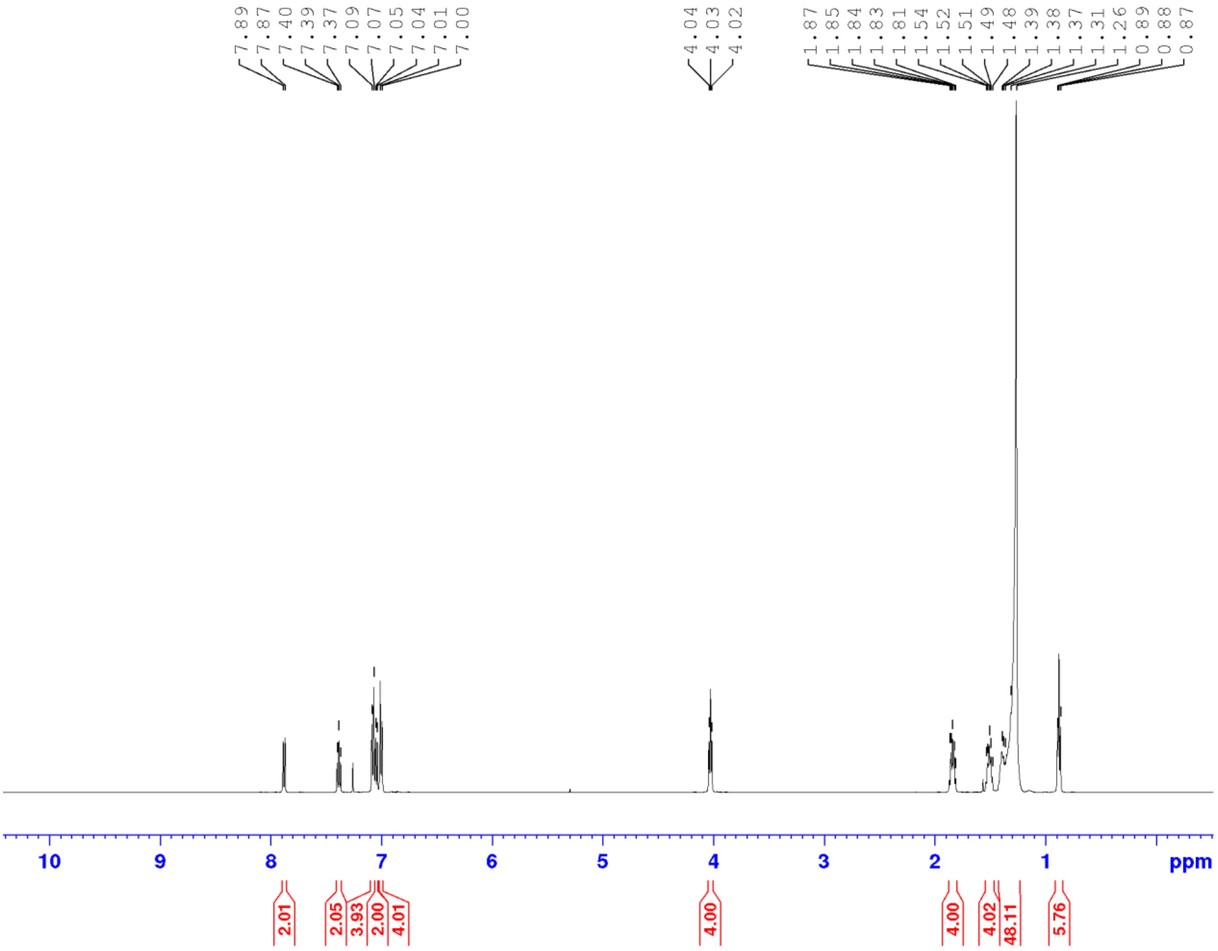

Figure S 32: ¹H NMR (500 MHz, $CDCl_3$, 25 °C) of N^1,N^2-bis(4-*n*-hexadecyloxyphenyl)acenaphthylene-1,2-diimine **OC16-BIAN**.

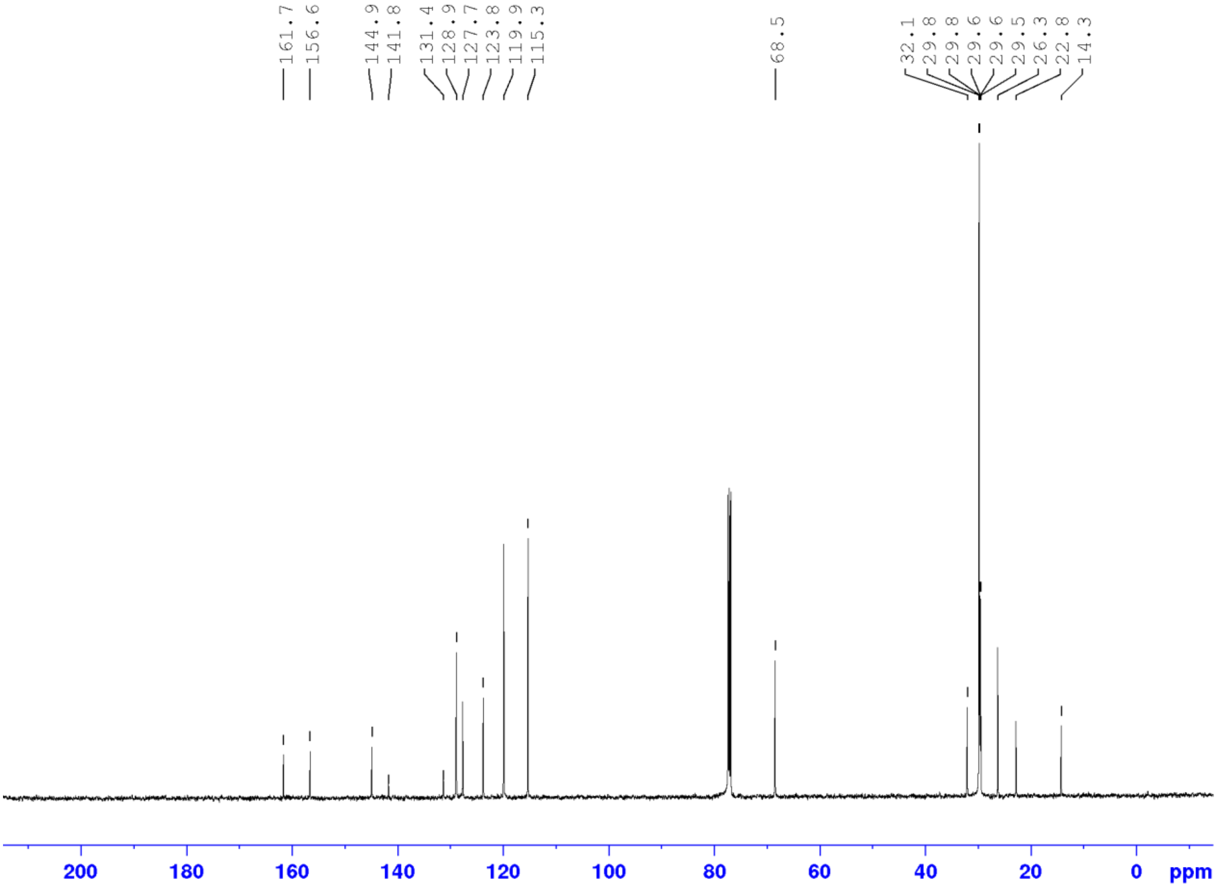

Figure S 33: ^{13}C NMR (126 MHz, $CDCl_3$, 25 °C) of *N*^{1},*N*^{2}-bis(4-*n*-hexadecyloxyphenyl) acenaphthylene-1,2-diimine **OC16-BIAN**.

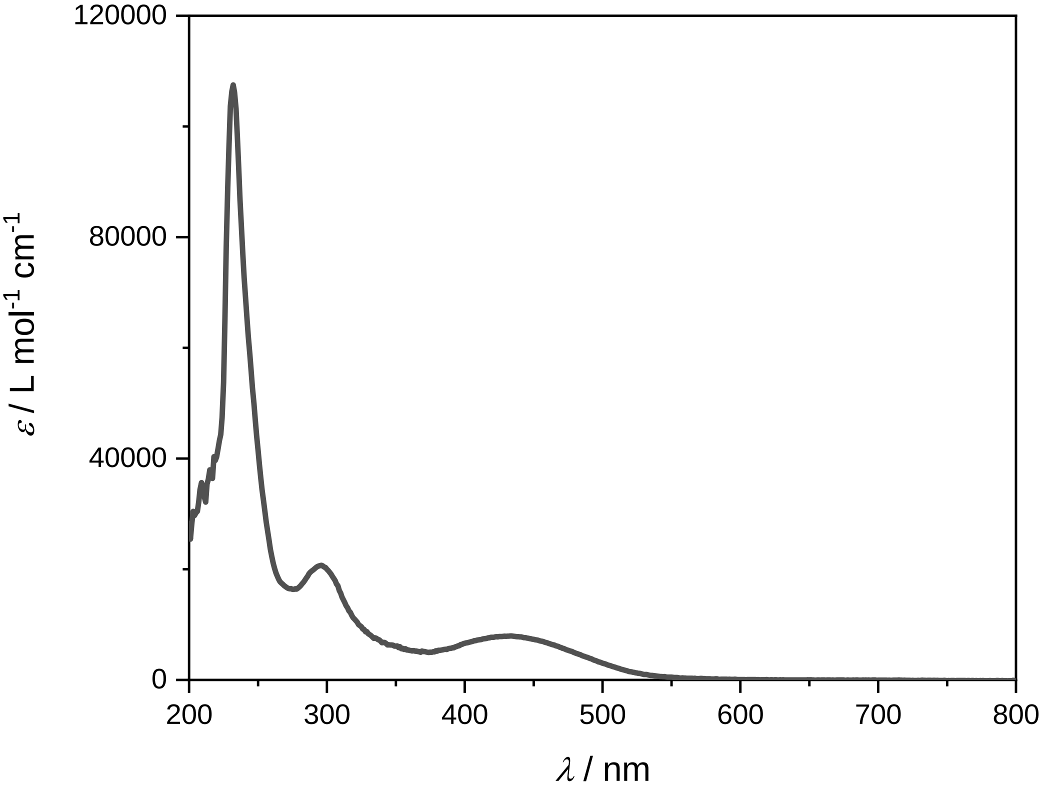

Figure S 34: UV-vis absorption spectrum of N^1,N^2-bis(4-*n*-hexadecyloxyphenyl)acenaphthylene-1,2-diimine **OC16-BIAN** in DCM.

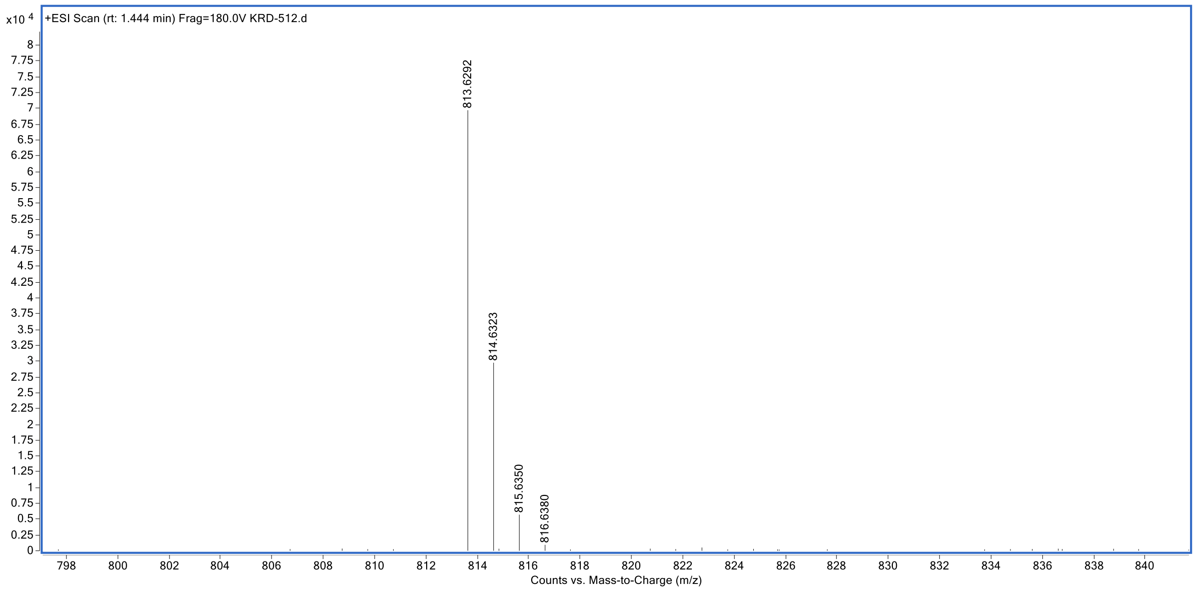

Figure S 35: HRMS spectrum of N^1,N^2-bis(4-*n*-hexadecyloxyphenyl)acenaphthylene-1,2-diimine **OC16-BIAN**.

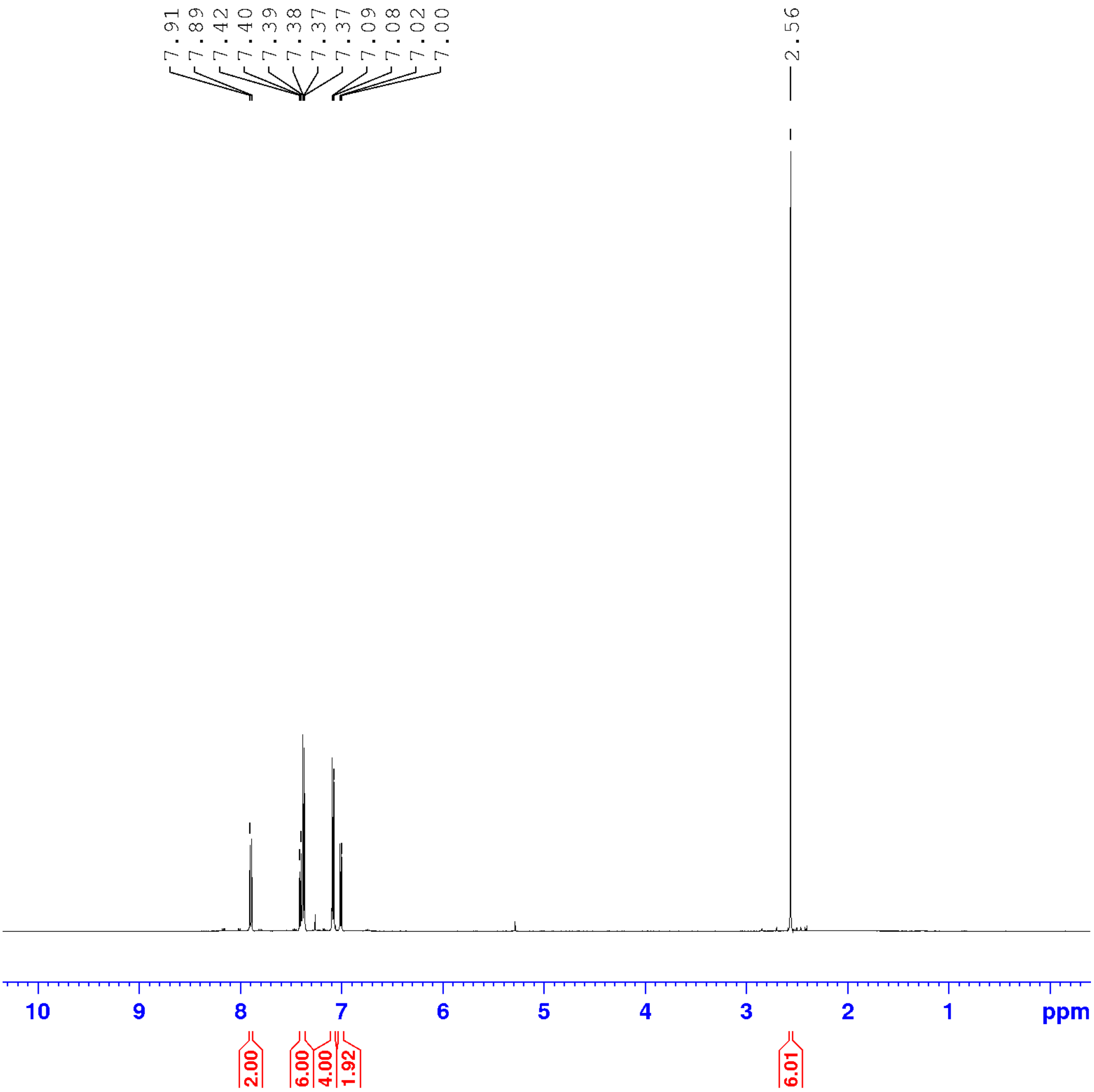

Figure S 36: ^{1}H NMR (500 MHz, $CDCl_3$, 25 °C) of N^1,N^2-bis(4-(methylthio)phenyl)acenaphthylene-1,2-diimine **SMe-BIAN**.

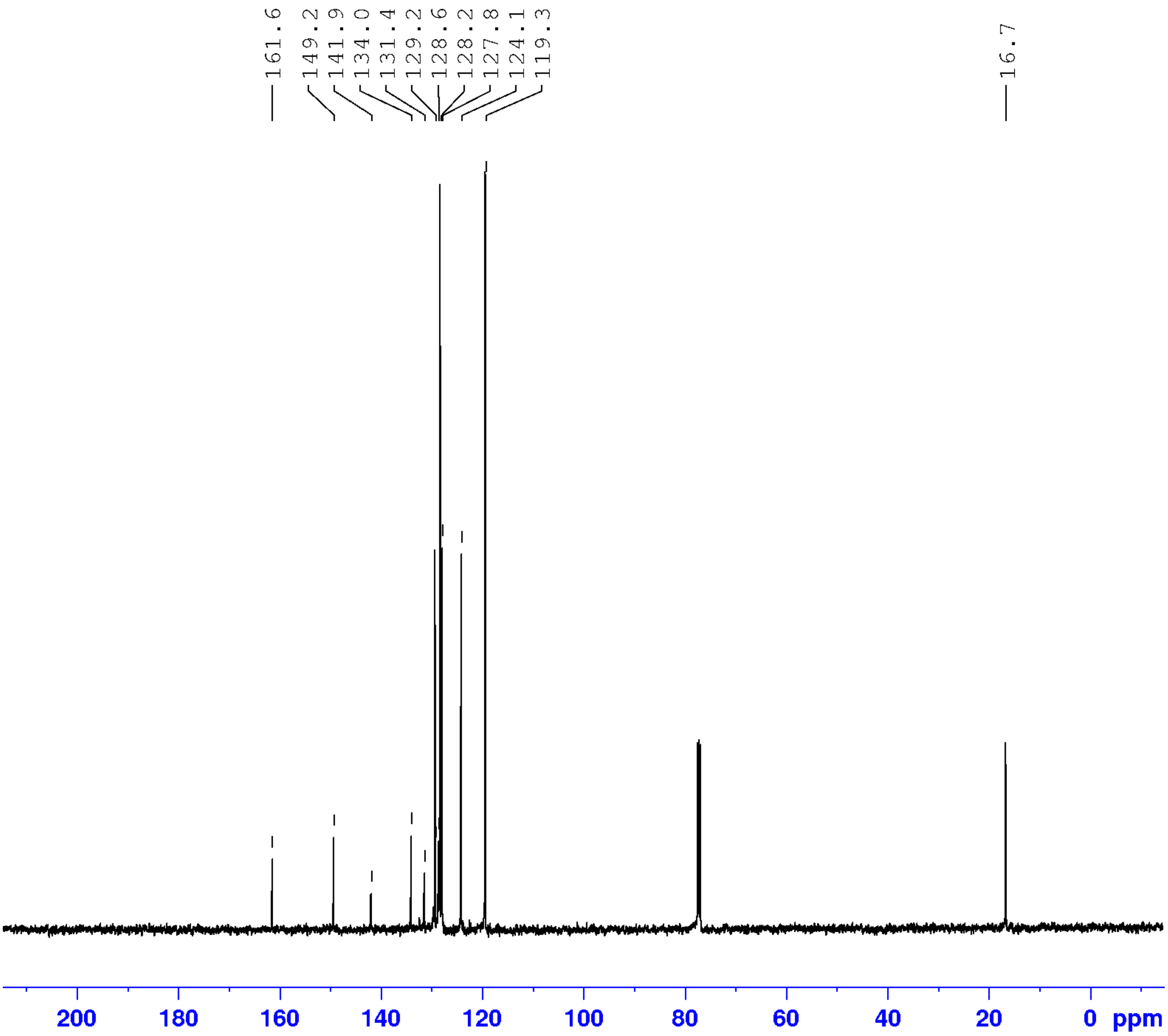

Figure S 37: ^{13}C NMR (126 MHz, $CDCl_3$, 25 °C) of N^1,N^2-bis(4-(methylthio)phenyl)acenaphthylene-1,2-diimine **SMe-BIAN**.

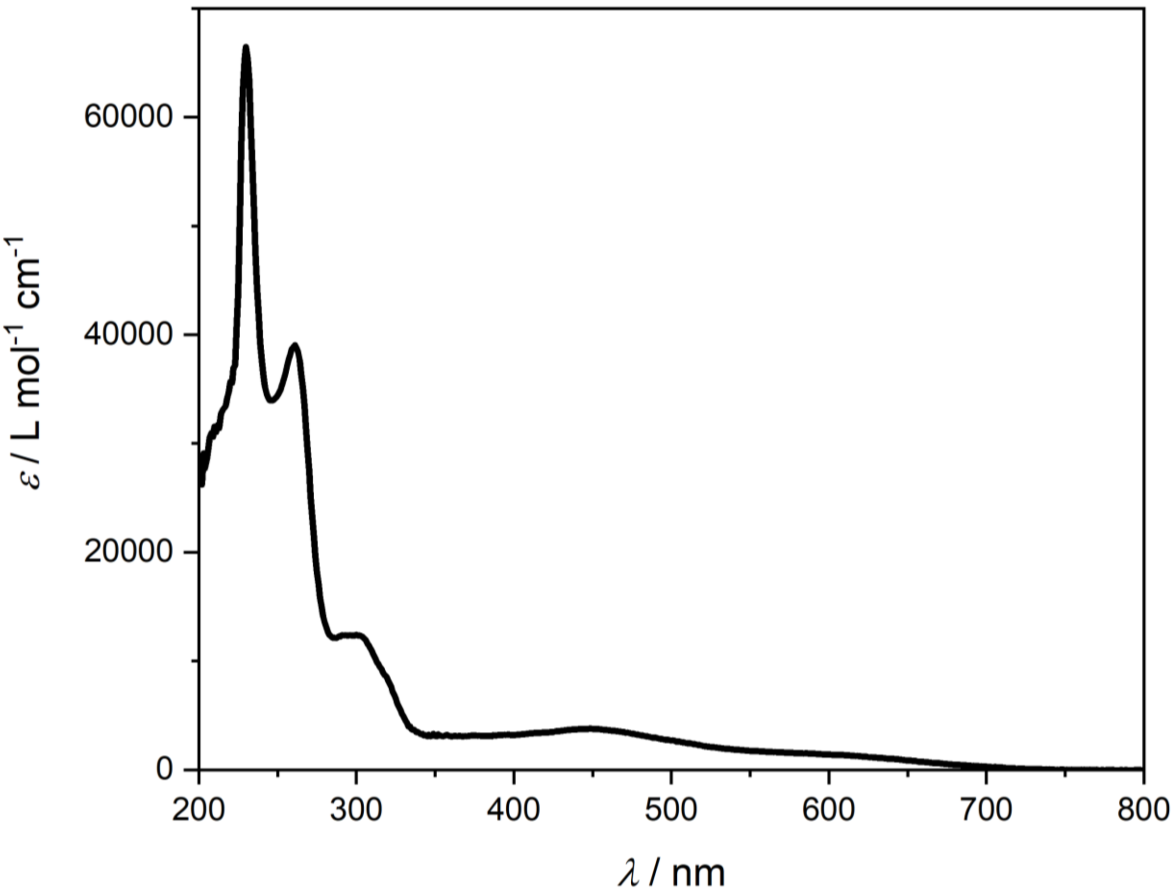

Figure S 38: UV-vis absorption spectrum of N^1,N^2-bis(4-(methylthio)phenyl)acenaphthylene-1,2-diimine **SMe-BIAN** in DCM.

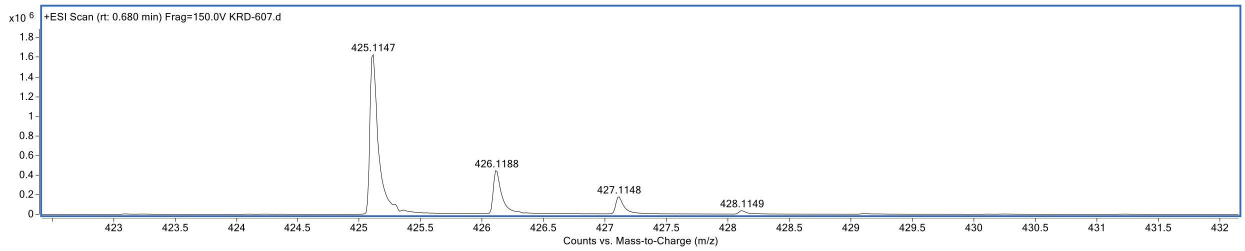

Figure S 39: HRMS spectrum of N^1,N^2-bis(4-(methylthio)phenyl)acenaphthylene-1,2-diimine **SMe-BIAN**.

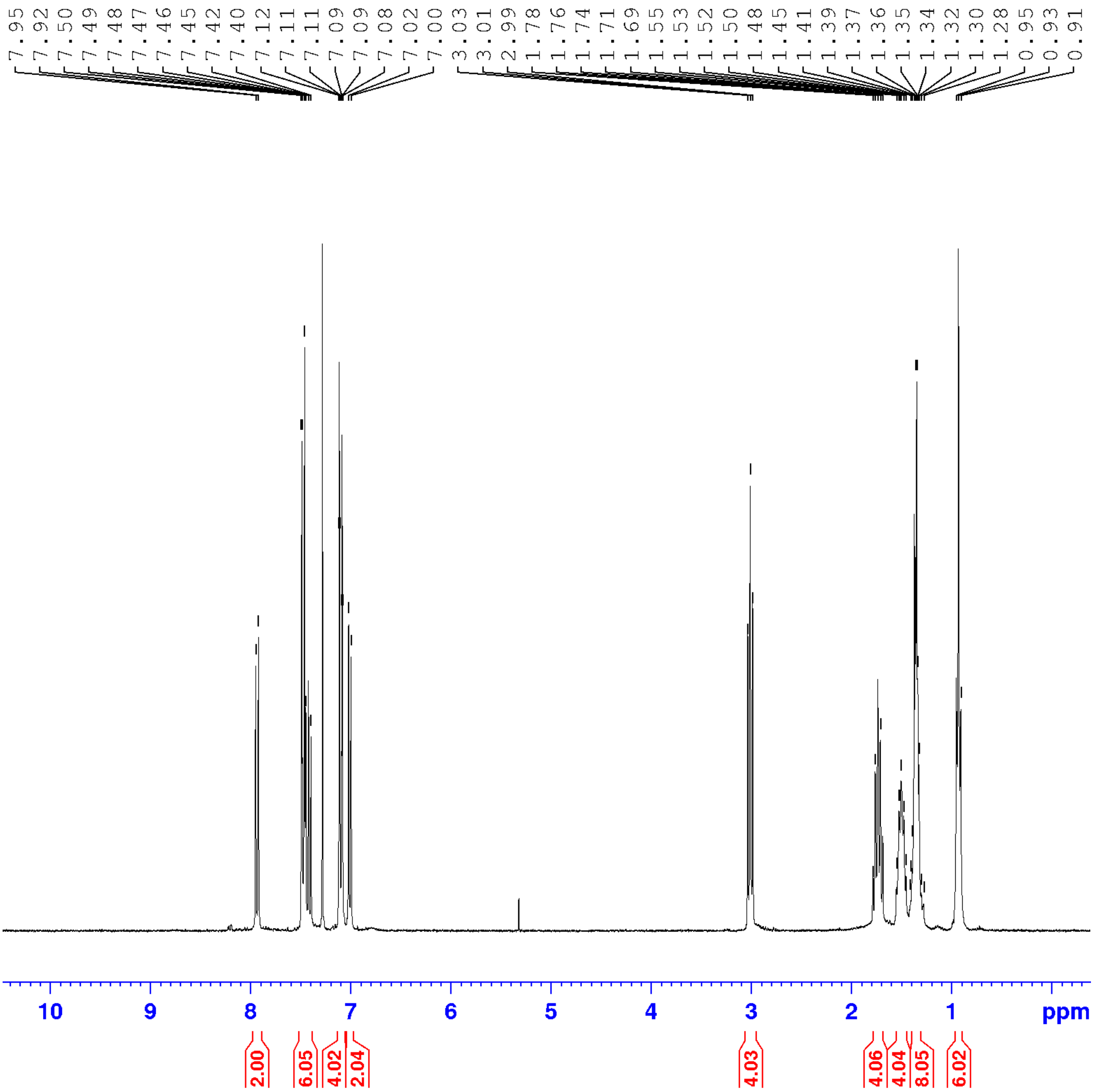

Figure S 40: ^{1}H NMR (300 MHz, $CDCl_3$, 25 °C) of N^1,N^2-bis(4-(*n*-hexylthio)phenyl)acenaphthylene-1,2-diimine **SC6-BIAN**.

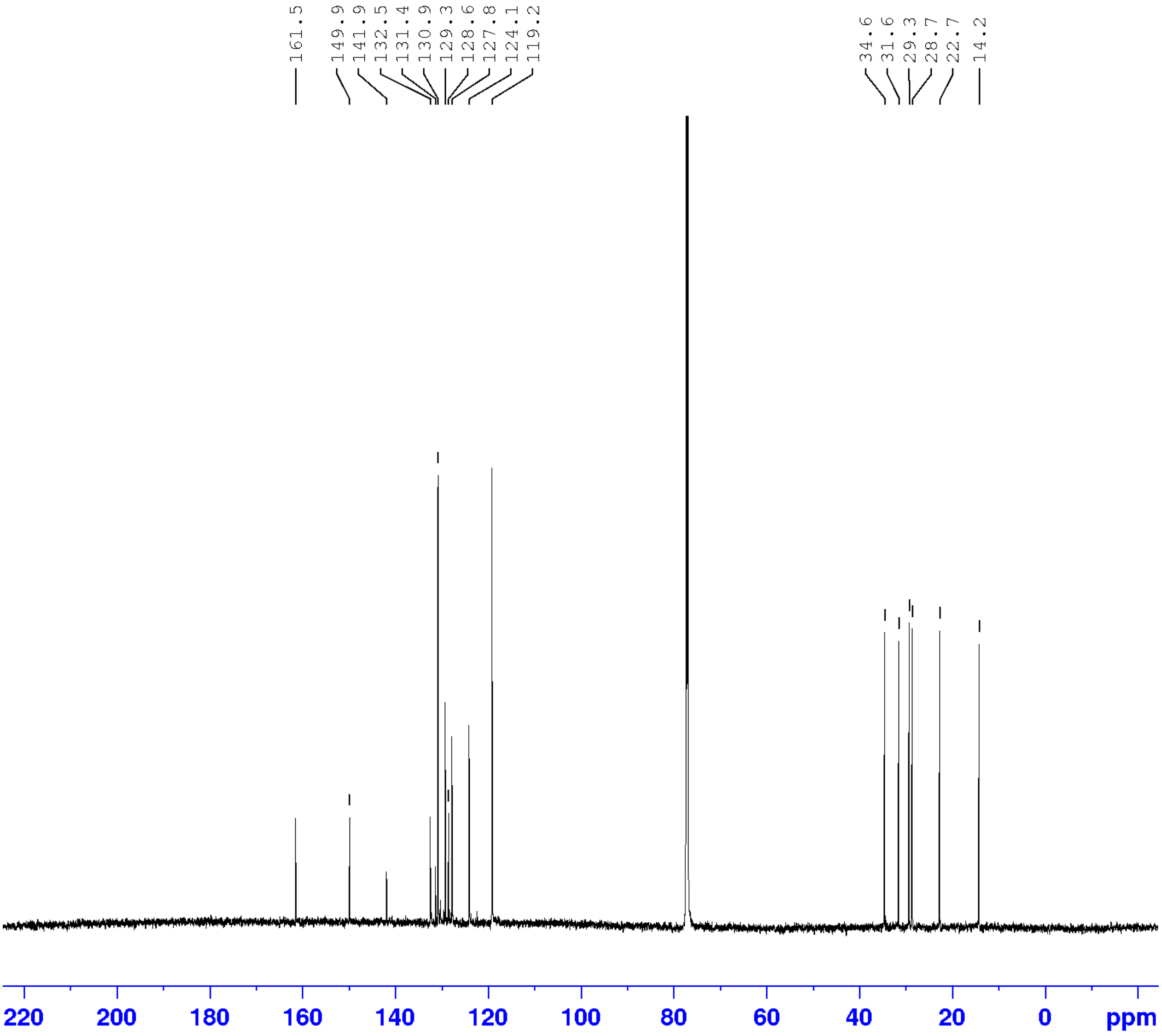

Figure S 41: ^{13}C NMR (126 MHz, $CDCl_3$, 25 °C) of N^1,N^2-bis(4-(*n*-hexylthio)phenyl)acenaphthylene-1,2-diimine **SC6-BIAN**.

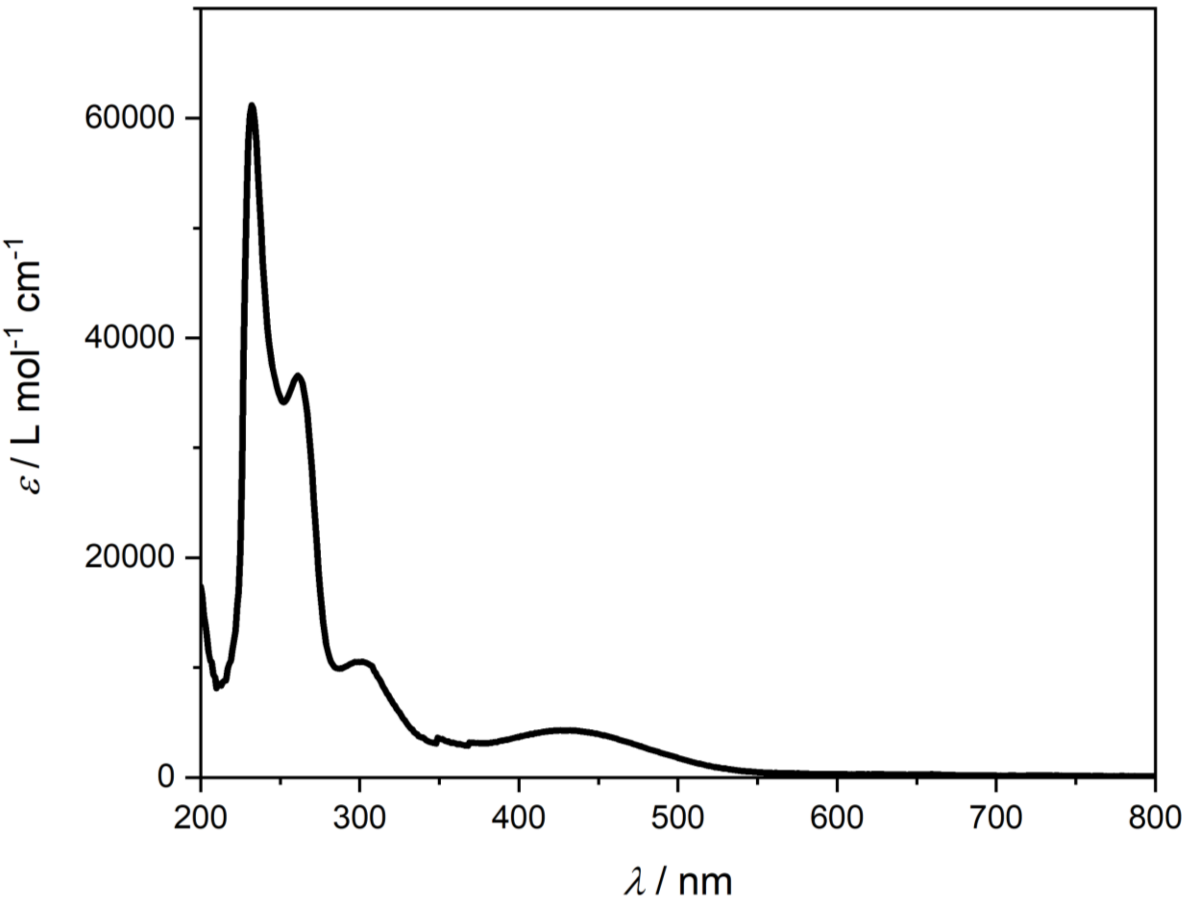

Figure S 42: UV-vis absorption spectrum of N^1,N^2-bis(4-(hexylthio)phenyl)acenaphthylene-1,2-diimine **SC6-BIAN** in DCM.

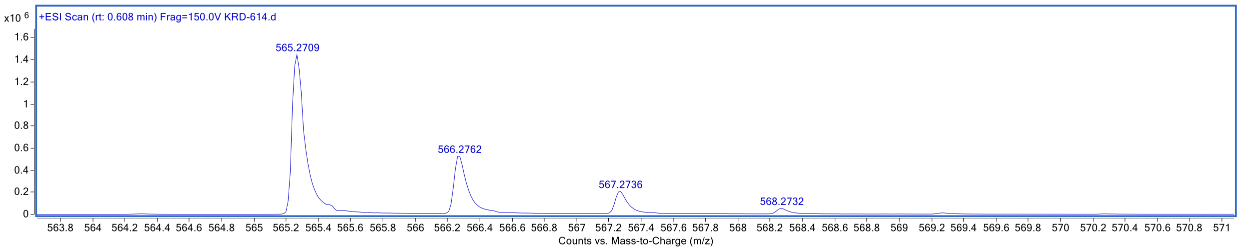

Figure S 43: HRMS spectrum of N^1,N^2-bis(4-(hexylthio)phenyl)acenaphthylene-1,2-diimine **SC6-BIAN**.

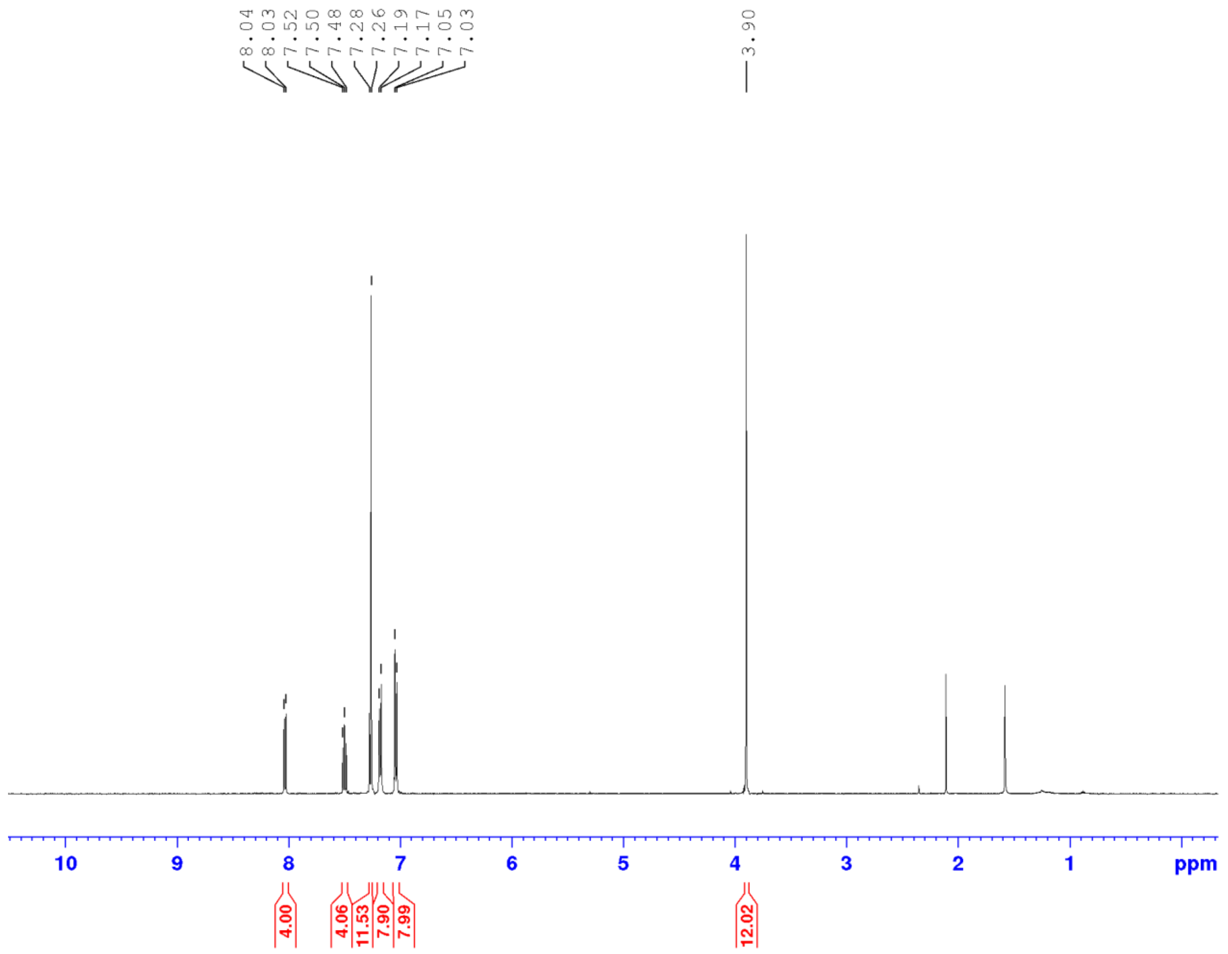

Figure S 44: ^{1}H NMR (500 MHz, $CDCl_3$, 25 °C) of $[Ag(4\text{-}OMe\text{-}BIAN)_2]BF_4$ **Ag-OMe**.

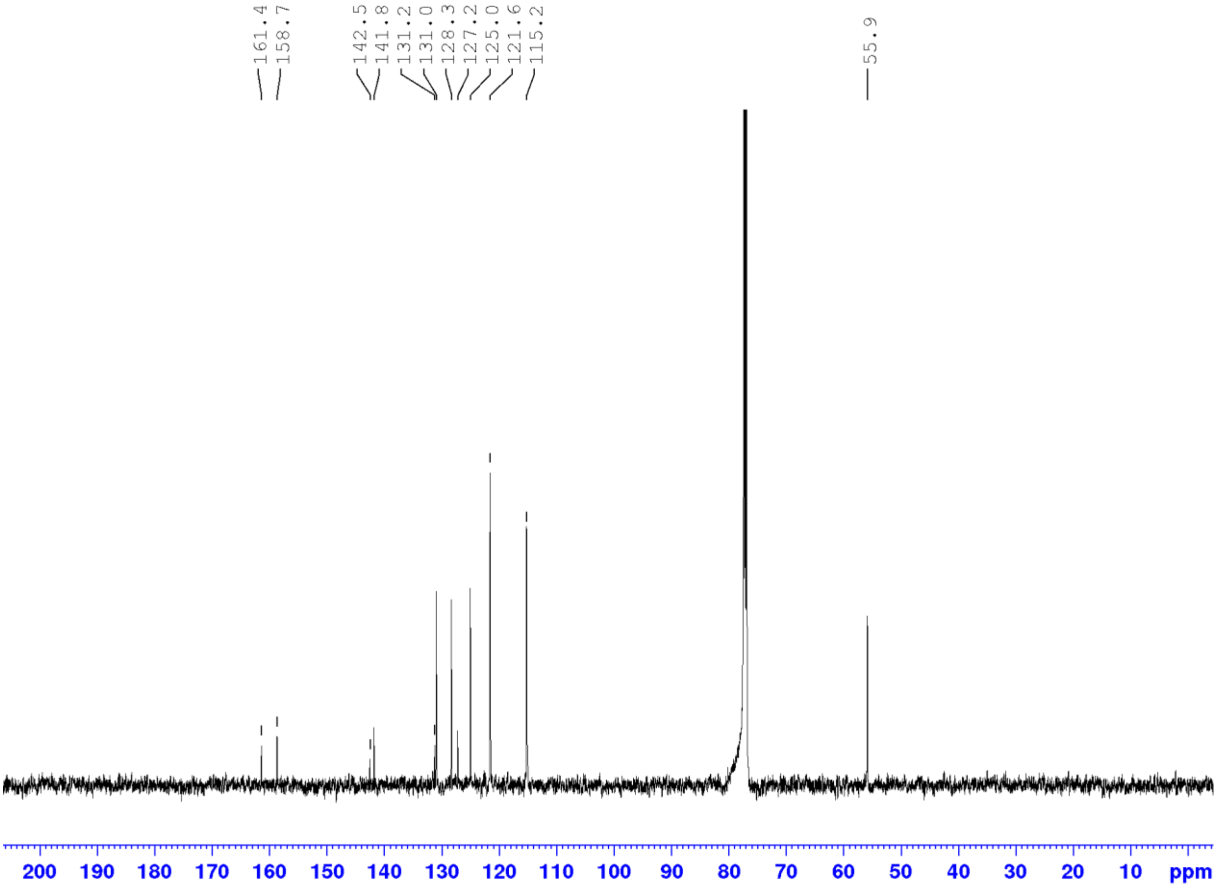

Figure S 45: ^{13}C NMR (126 MHz, $CDCl_3$, 25 °C) of $[Ag(4\text{-OMe-BIAN})_2]BF_4$ **Ag-OMe**.

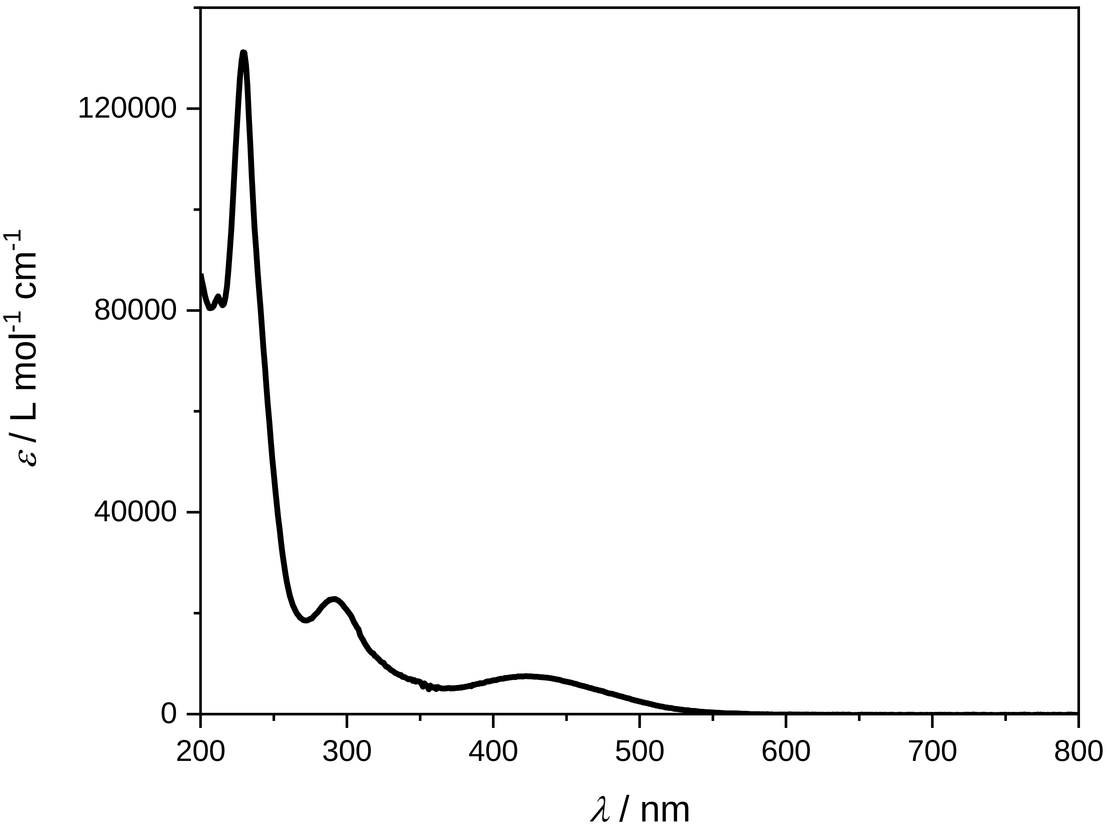

Figure S 46: UV-vis absorption spectrum of [Ag(4-OMe-BIAN)$_2$]BF_4 **Ag-OMe** in ACN.

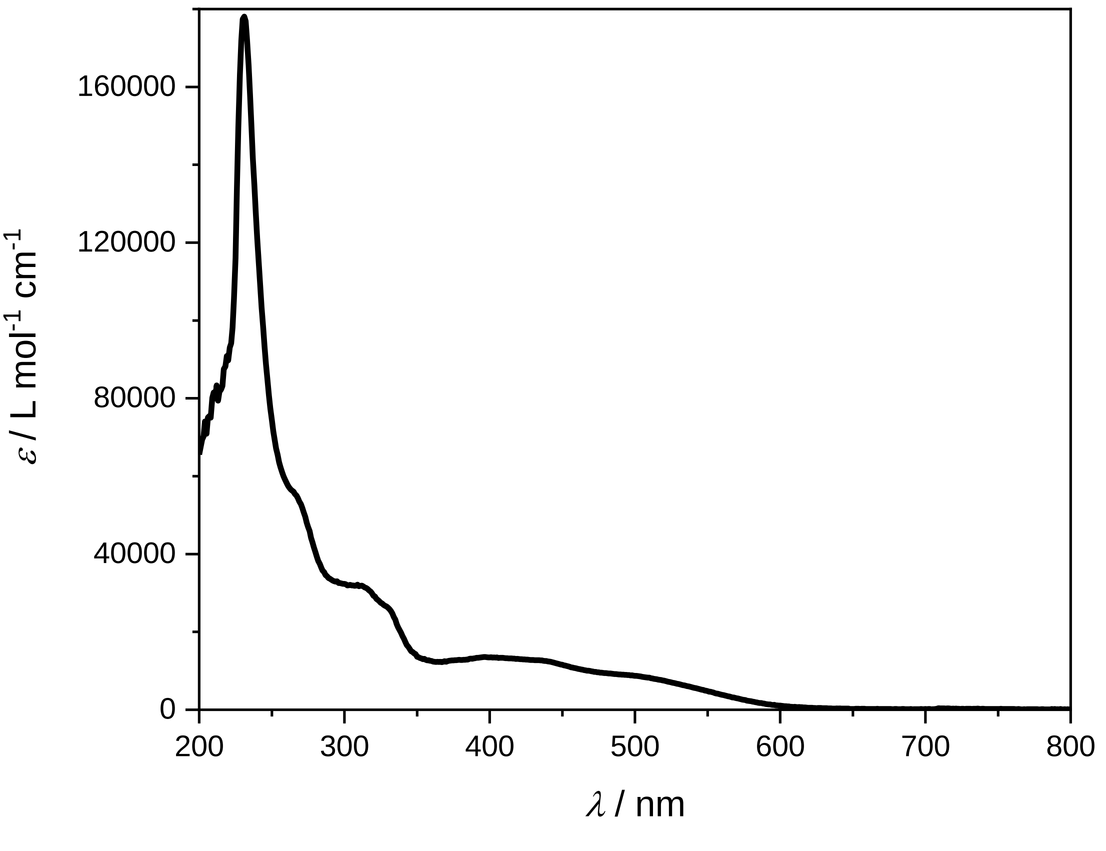

Figure S 47: UV-vis absorption spectrum of [Ag(4-OMe-BIAN)$_2$]BF_4 **Ag-OMe** in DCM.

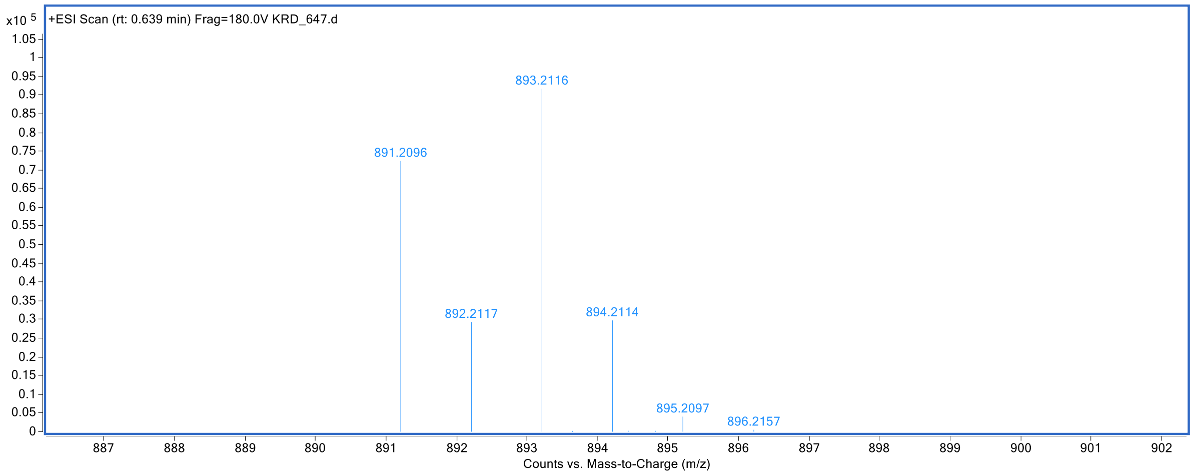

Figure S 48: HRMS spectrum of $[Ag(4\text{-}OMe\text{-}BIAN)_2]BF_4$ **Ag-OMe**.

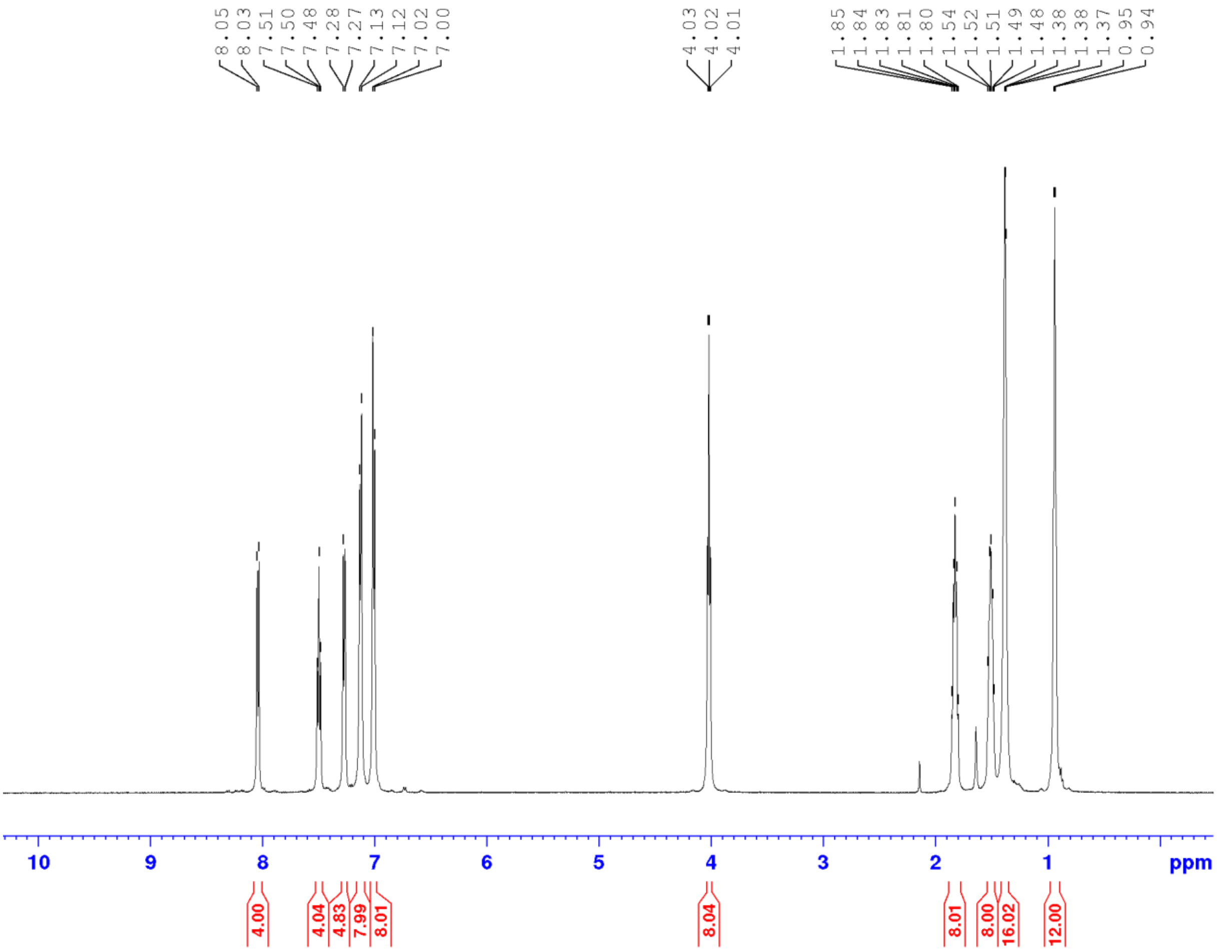

Figure S 49: ^{1}H NMR (500 MHz, $CDCl_3$, 25 °C) of $[Ag(4\text{-}n\text{-}hexyloxy\text{-}BIAN)_2]BF_4$ **Ag-OC6**.

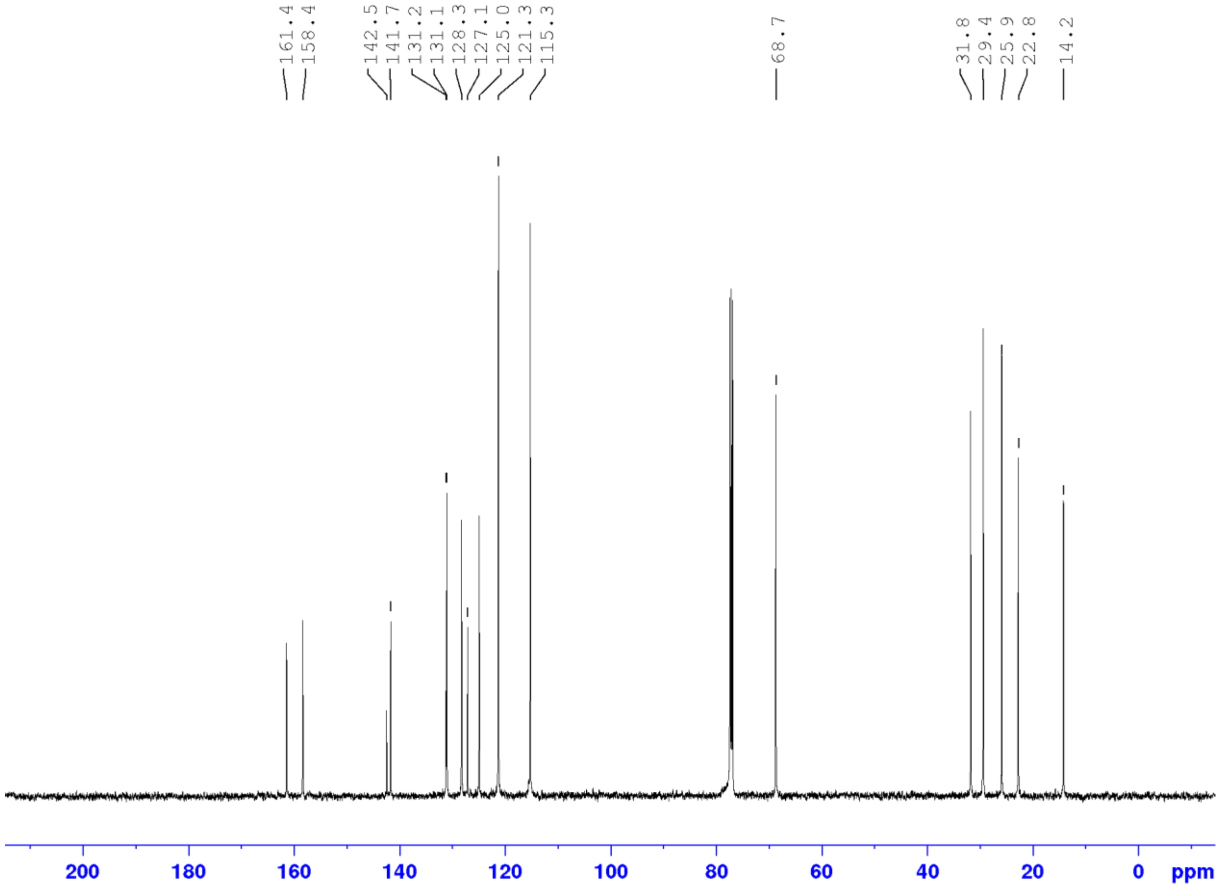

Figure S 50: ^{13}C NMR (126 MHz, $CDCl_3$, 25 °C) of $[Ag(4\text{-}n\text{-hexyloxy-BIAN})_2]BF_4$ **Ag-OC6**.

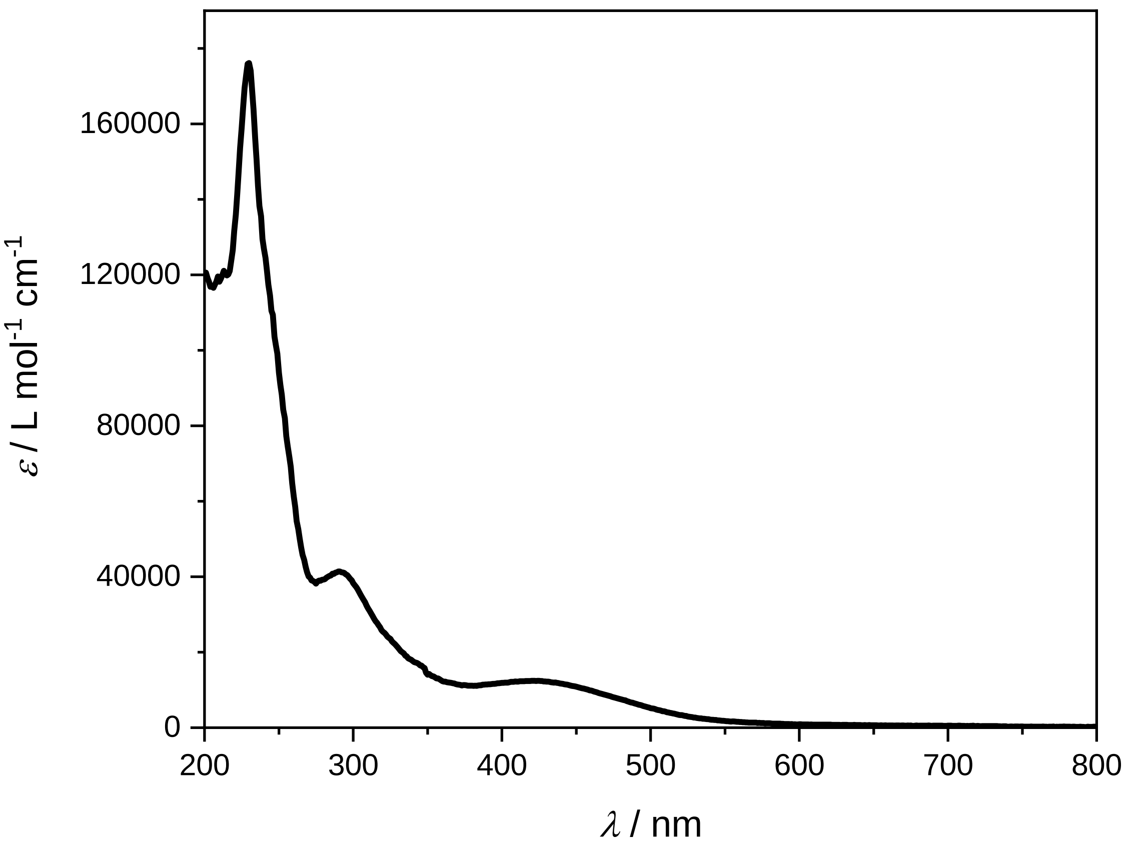

Figure S 51: UV-vis absorption spectrum of [Ag(4-*n*-hexyloxy-BIAN)$_2$]BF_4 **Ag-OC6**in ACN.

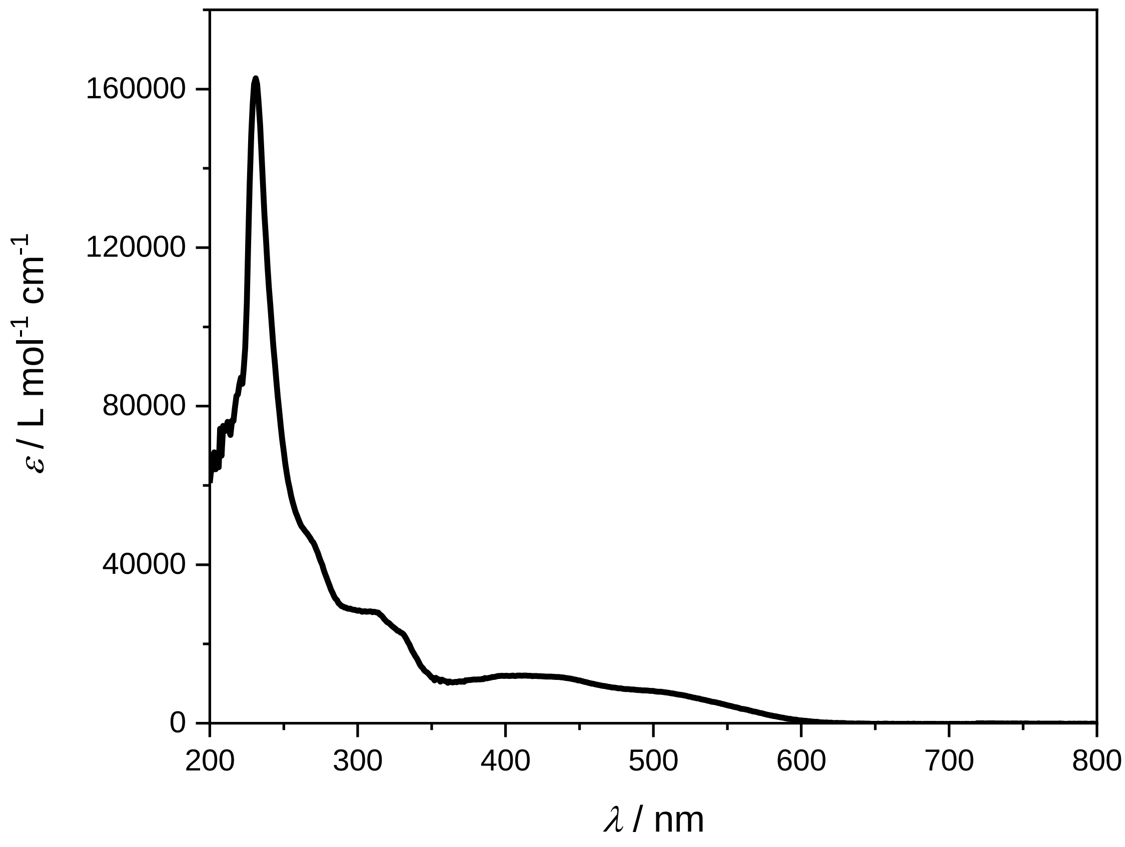

Figure S 52: UV-vis absorption spectrum of [Ag(4-*n*-hexyloxy-BIAN)$_2$]BF_4 **Ag-OC6**in DCM.

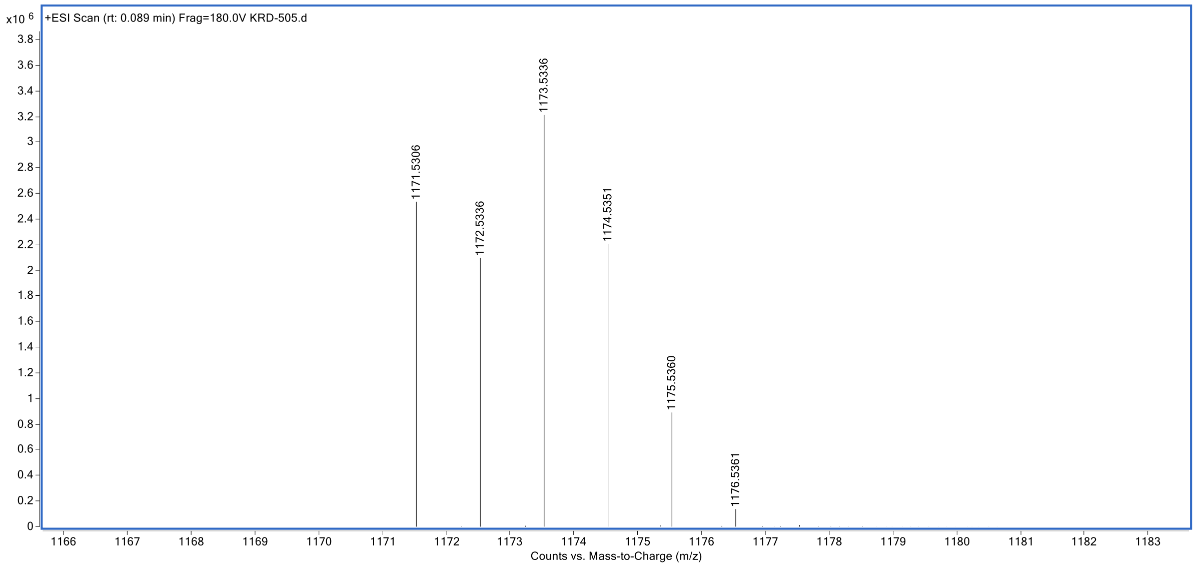

Figure S 53: HRMS spectrum of [Ag(4-*n*-hexyloxy-BIAN)$_2$]BF_4 **Ag-OC6**.

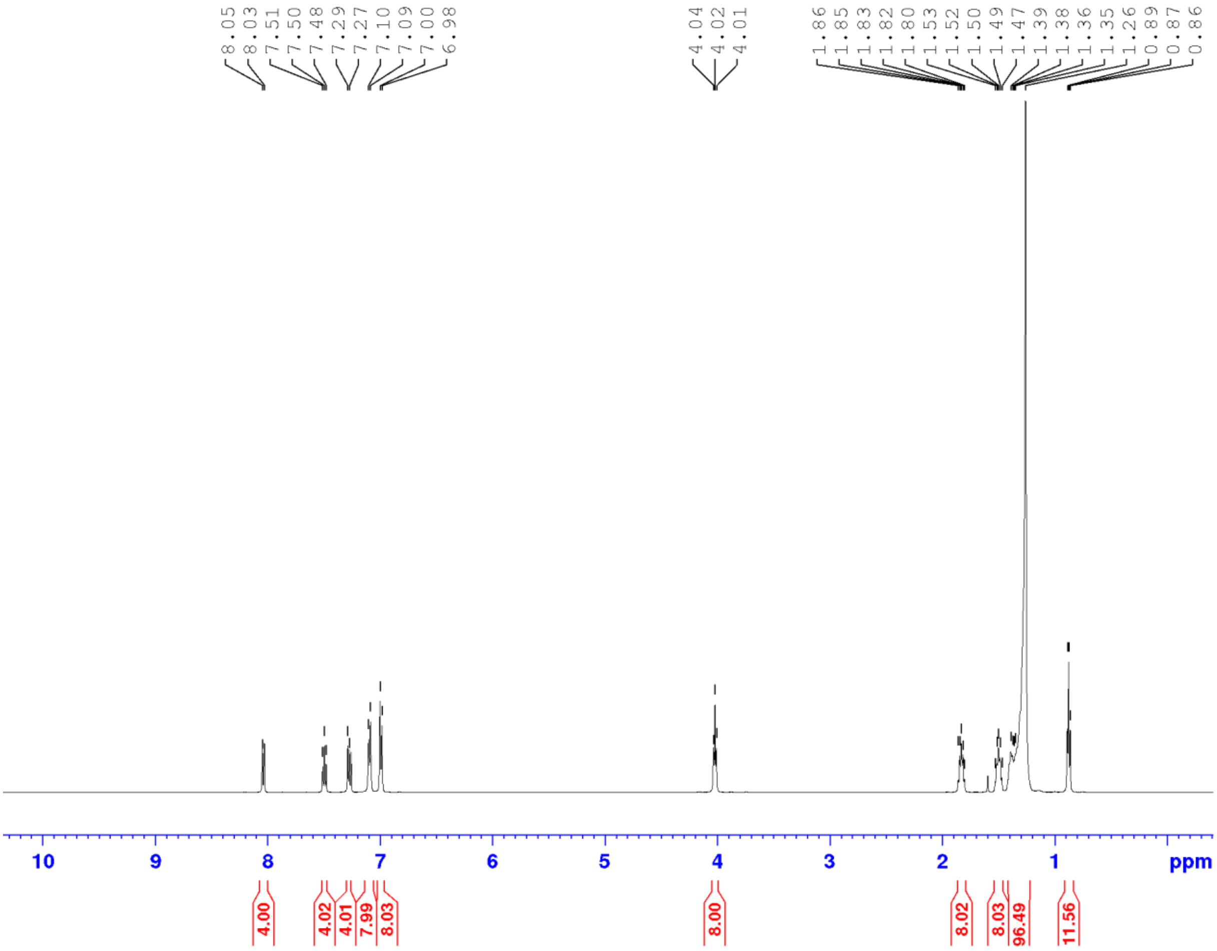

Figure S 54: ^{1}H NMR (500 MHz, $CDCl_3$, 25 °C) of [Ag(4-*n*-hexadecyloxy-BIAN)$_2$]BF_4 **Ag-OC16**.

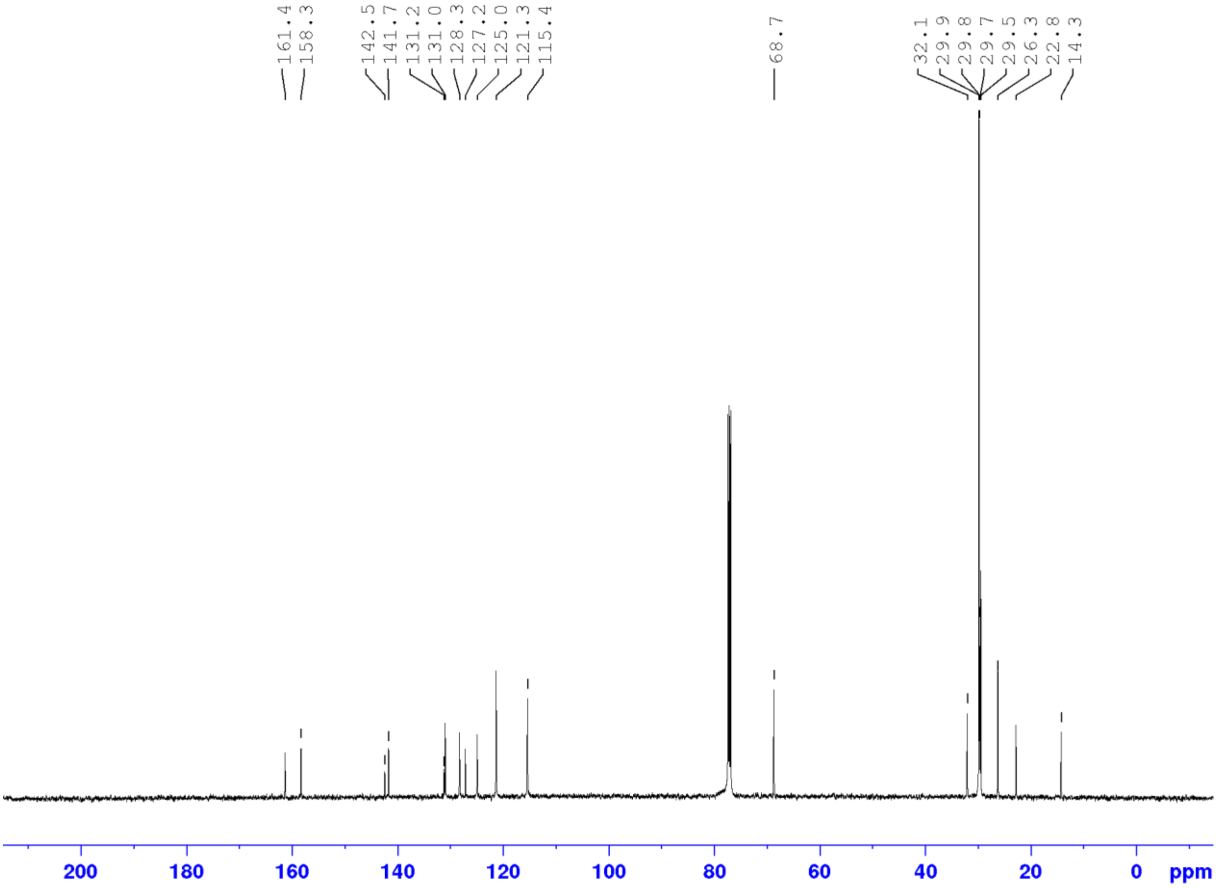

Figure S 55: ^{13}C NMR (126 MHz, $CDCl_3$, 25 °C) of $[Ag(4\text{-}n\text{-hexadecyloxy-BIAN})_2]BF_4$ **Ag-OC16**.

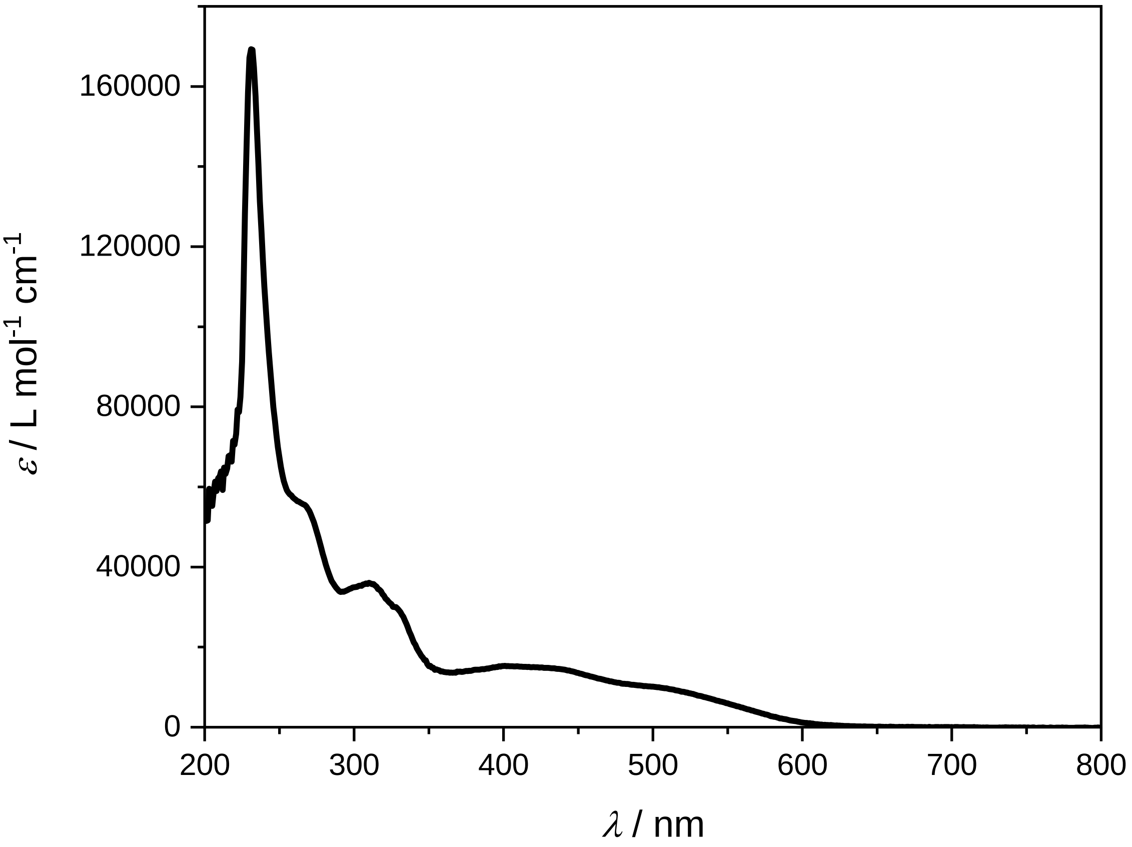

Figure S 56: UV-vis absorption spectrum of $[Ag(4\text{-}n\text{-hexadecyloxy-BIAN})_2]BF_4$ **Ag-OC16** in DCM.

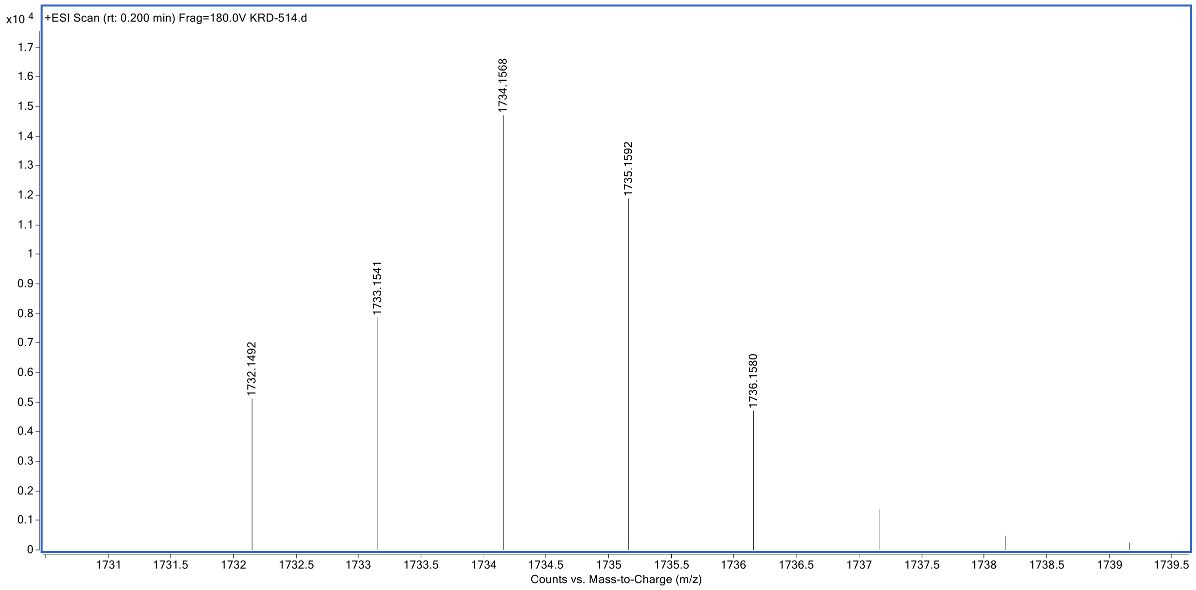

Figure S 57: HRMS spectrum of $[Ag(4\text{-}n\text{-hexadecyloxy-BIAN})_2]BF_4$ **Ag-OC16**.

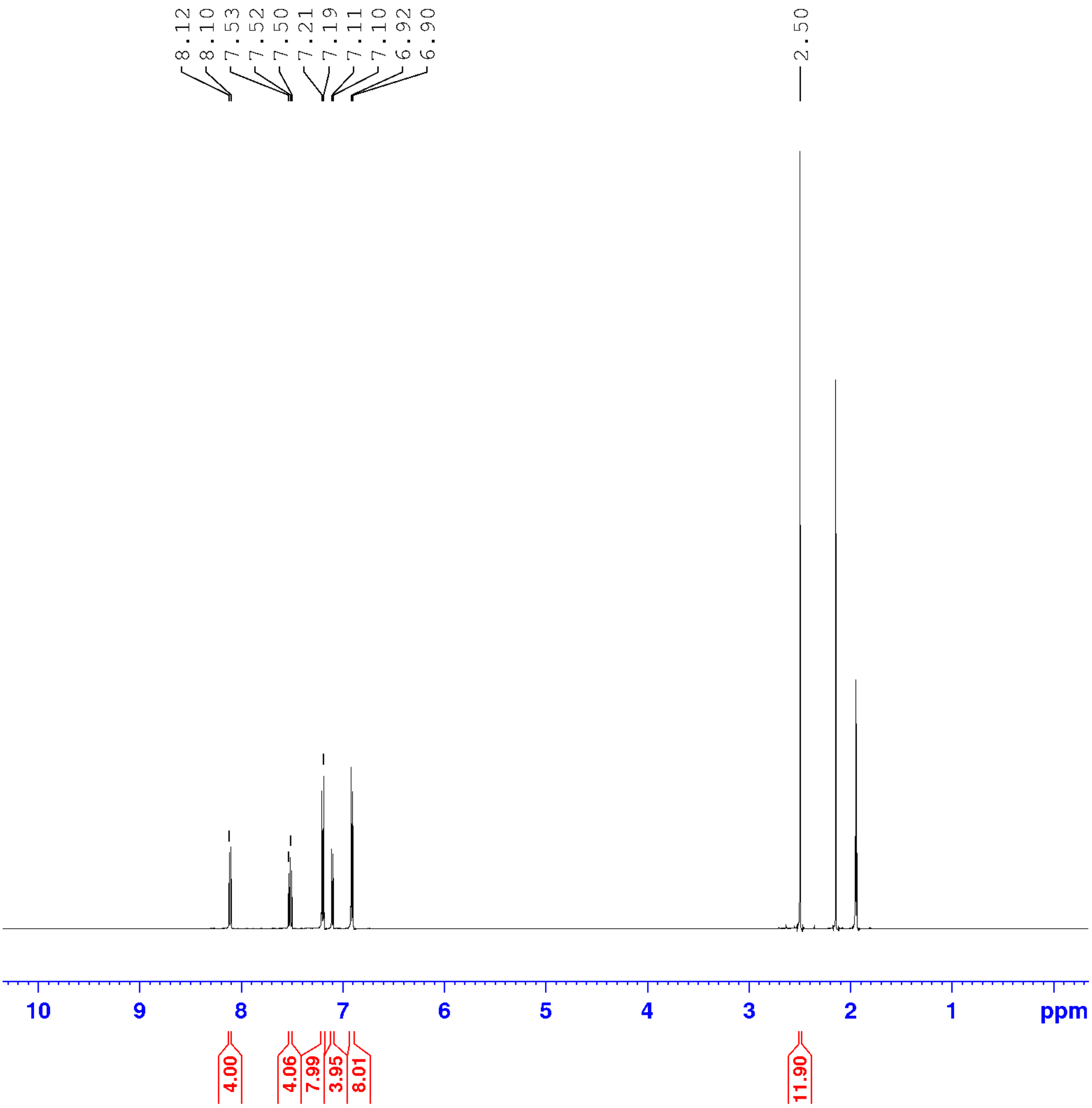

Figure S 58: ^{1}H NMR (500 MHz, CD_3CN, 25 °C) of $[Ag(4\text{-}SMe\text{-}BIAN)_2]BF_4$ **Ag-SMe**.

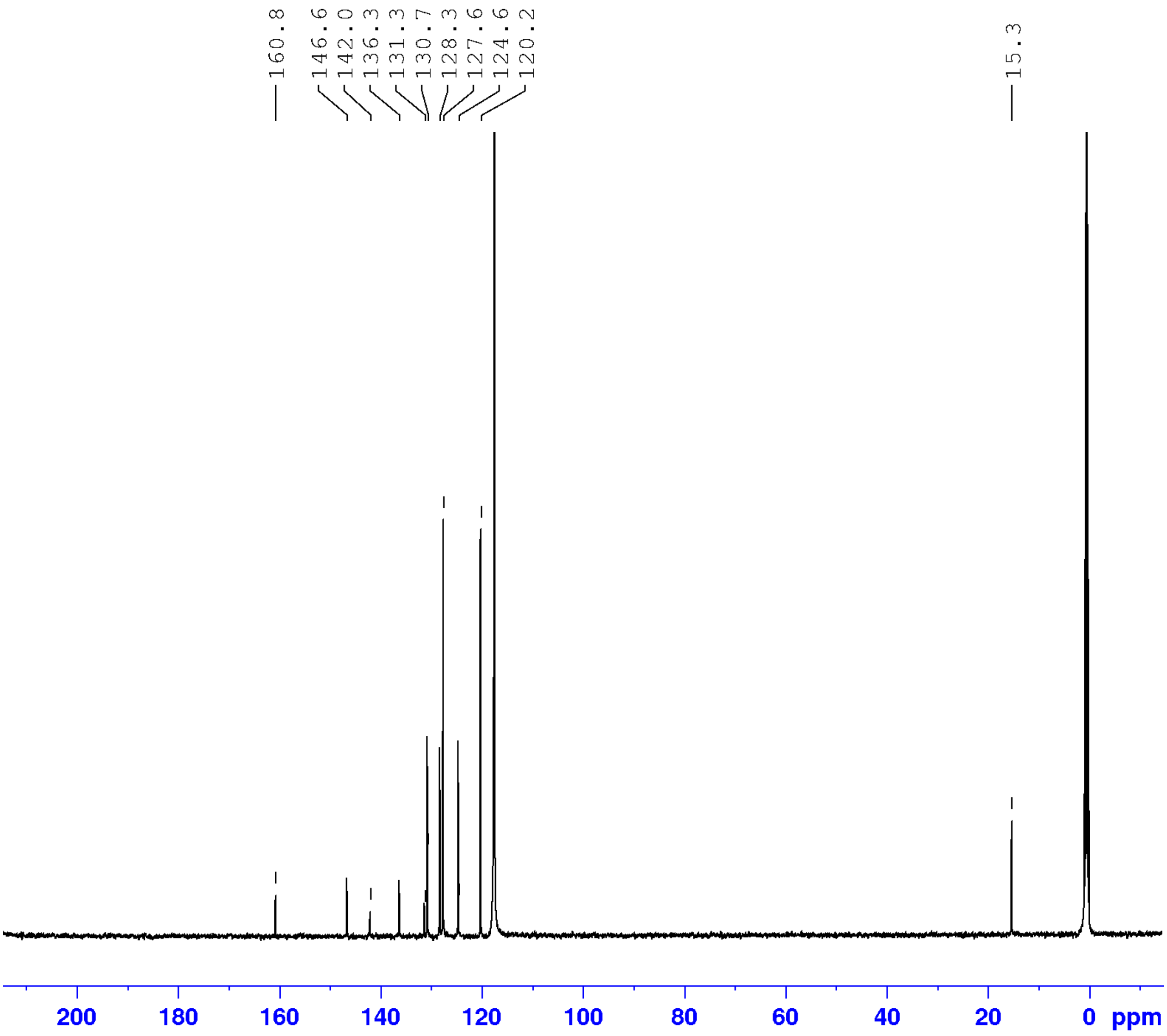

Figure S 59: ^{13}C NMR (126 MHz, CD_3CN, 25 °C) of [Ag(4-SMe-BIAN)$_2$]BF_4 **Ag-SMe**.

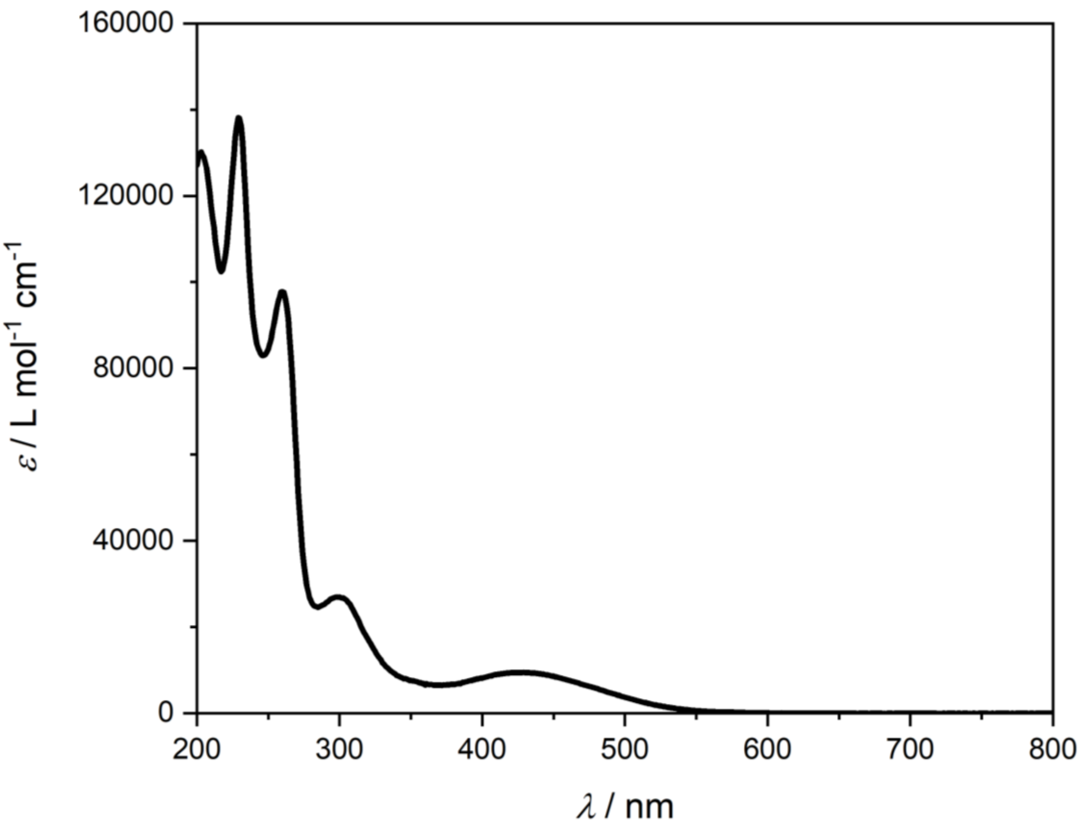

Figure S 60: UV-vis absorption spectrum of $[Ag(4\text{-}SMe\text{-}BIAN)_2]BF_4$ **Ag-SMe** in ACN.

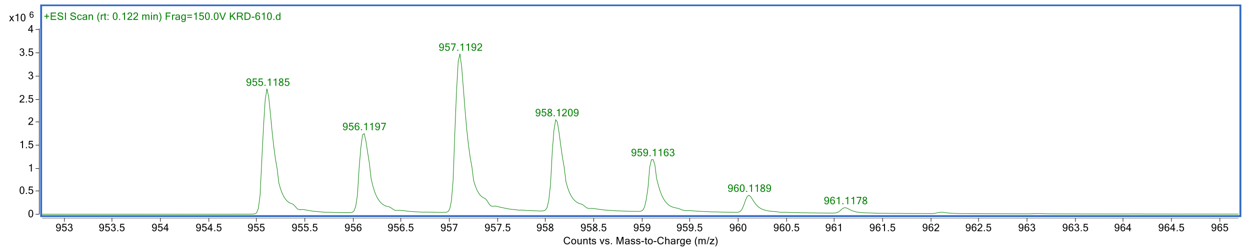

Figure S 61: HRMS spectrum of $[Ag(4\text{-}SMe\text{-}BIAN)_2]BF_4$ **Ag-SMe**.

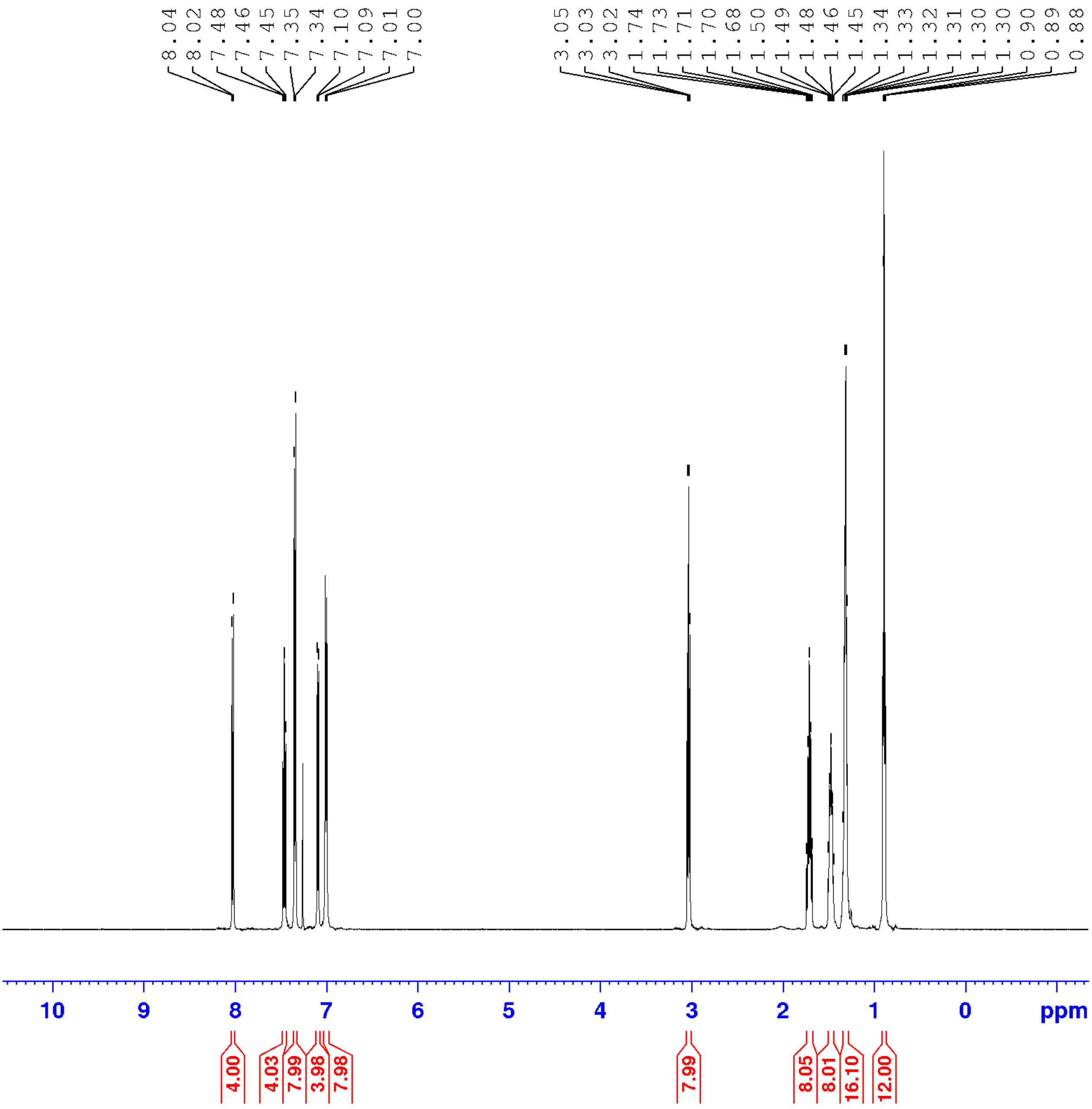

Figure S 62: ^{1}H NMR (500 MHz, $CDCl_3$, 25 °C) of $[Ag(4\text{-}SC_6\text{-}BIAN)_2]BF_4$ **Ag-SC6**.

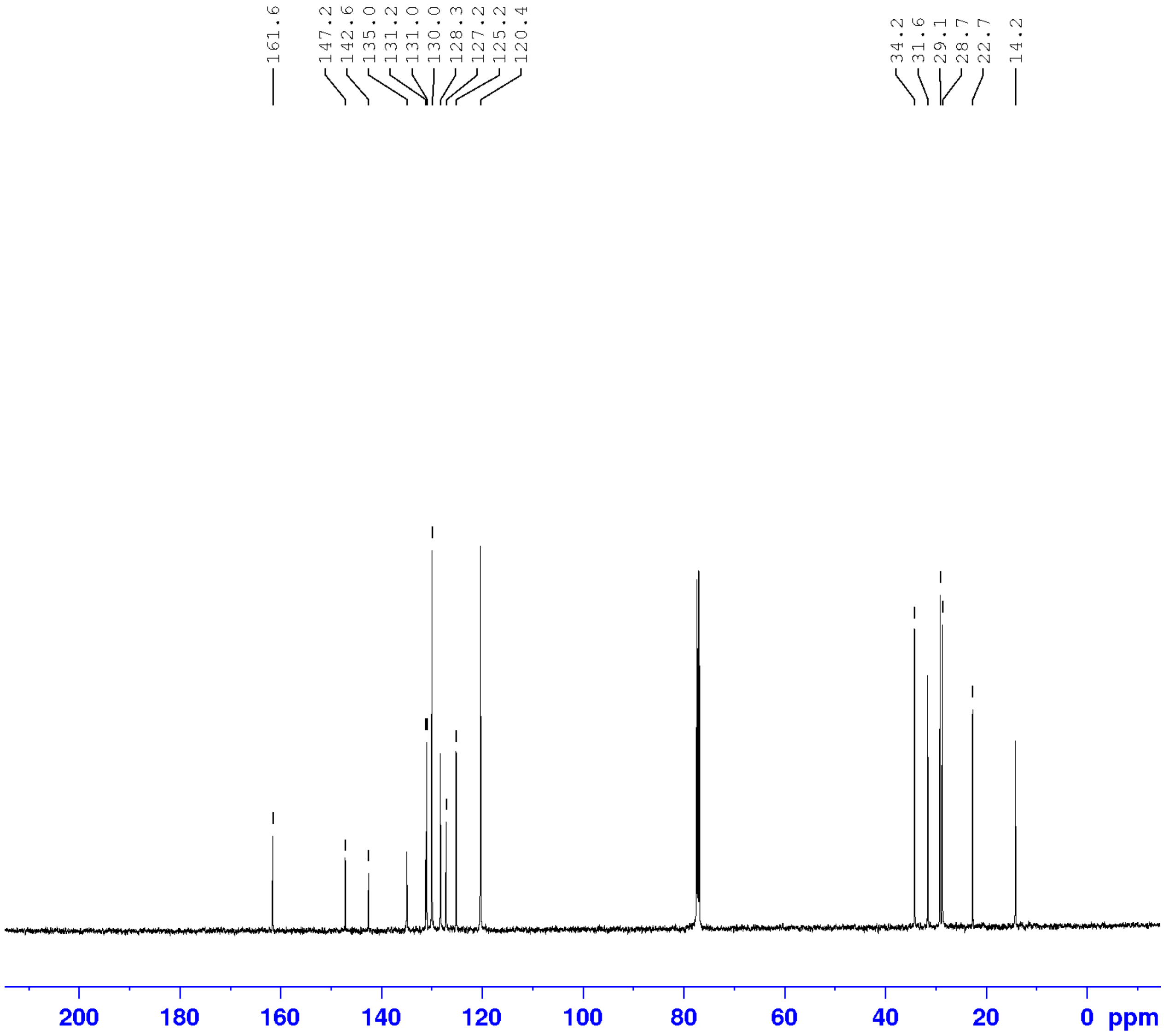

Figure S 63: ^{13}C NMR (126 MHz, $CDCl_3$, 25 °C) of [Ag(4-SC_6-BIAN)$_2$]BF_4 **Ag-SC6**.

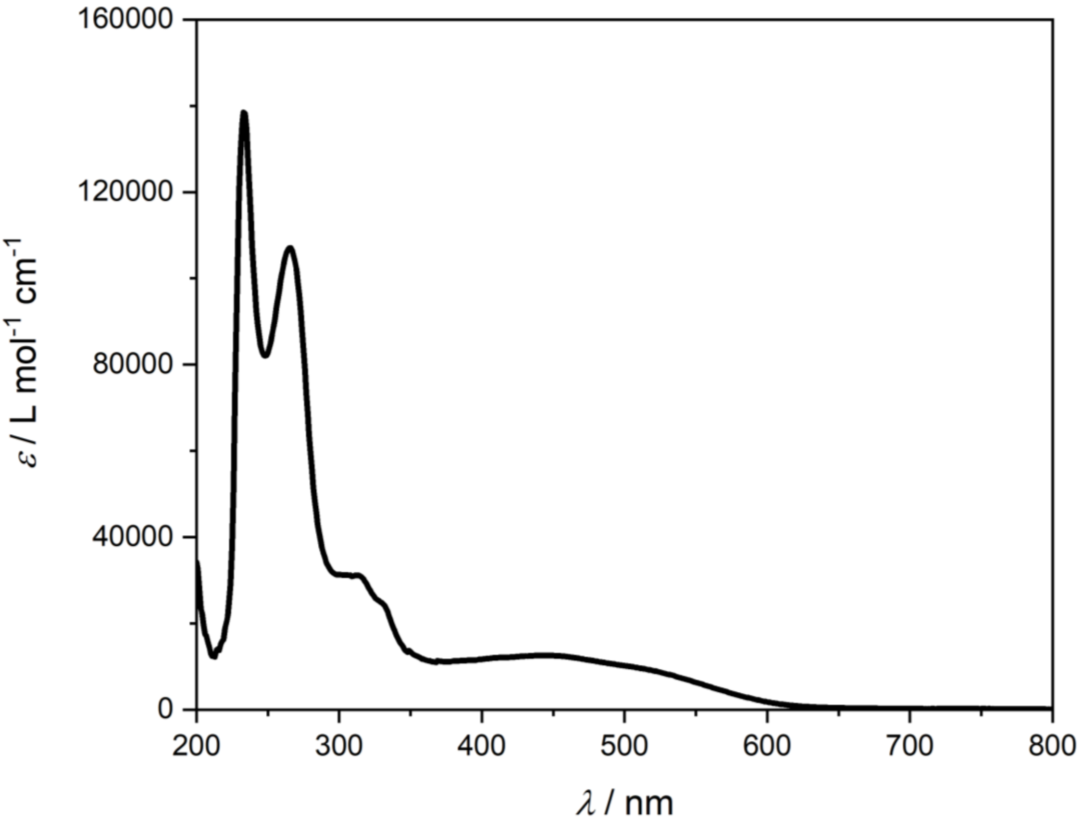

Figure S 64: UV-vis absorption spectrum of [Ag(4-SC_6-BIAN)$_2$]BF_4 **Ag-SC6**in DCM.

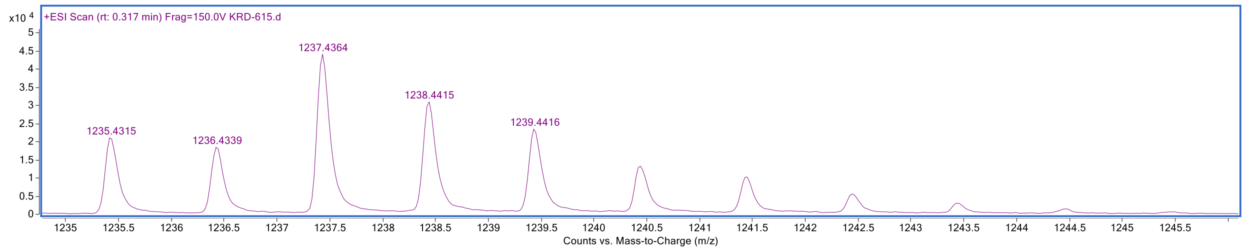

Figure S 65: HRMS spectrum of [Ag(4-SC_6-BIAN)$_2$]BF_4 **Ag-SC6**.

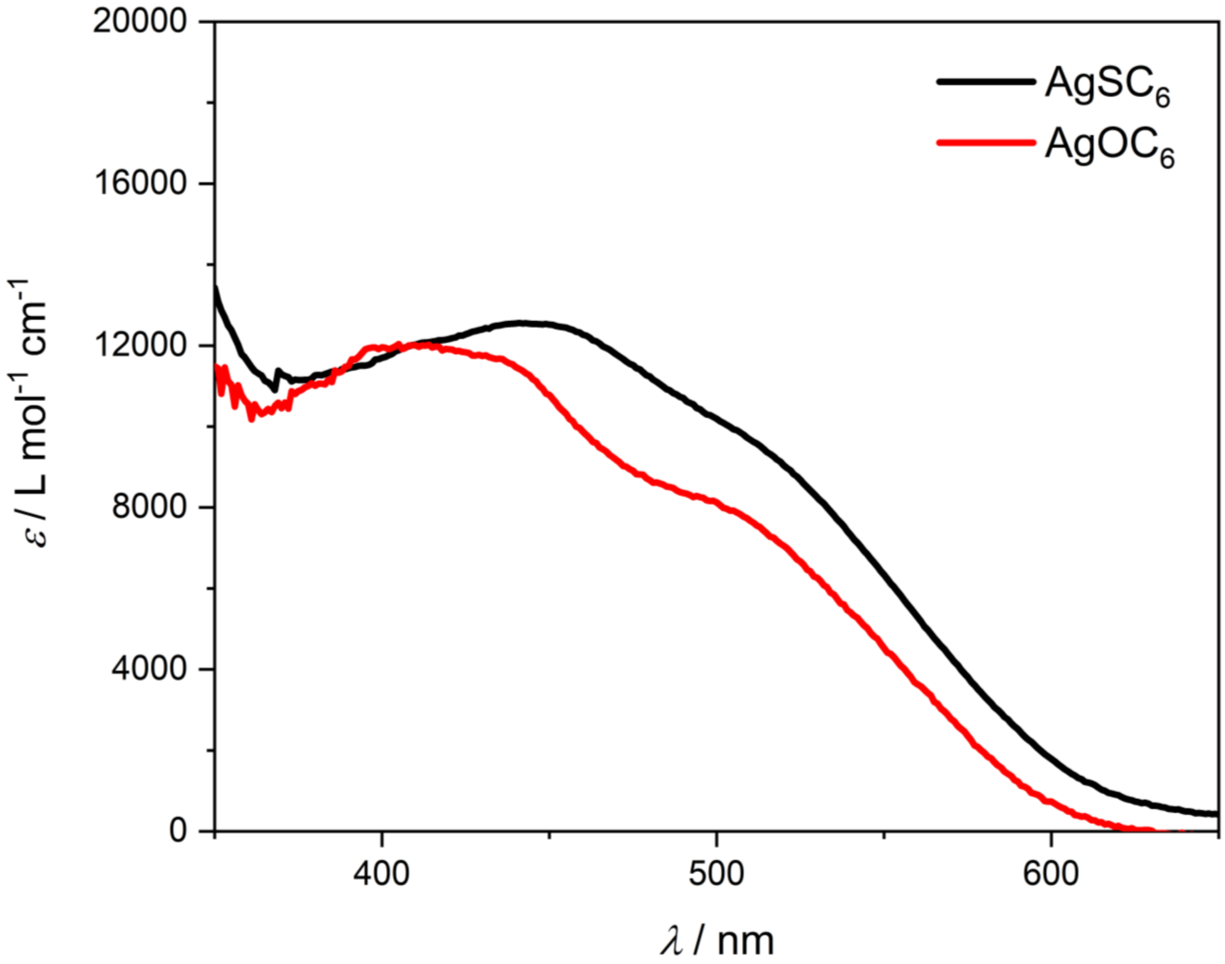

Figure S 66: Zoomed overlay of UV-Vis spectra from **Ag-OC6** and **Ag-SC6** in DCM.

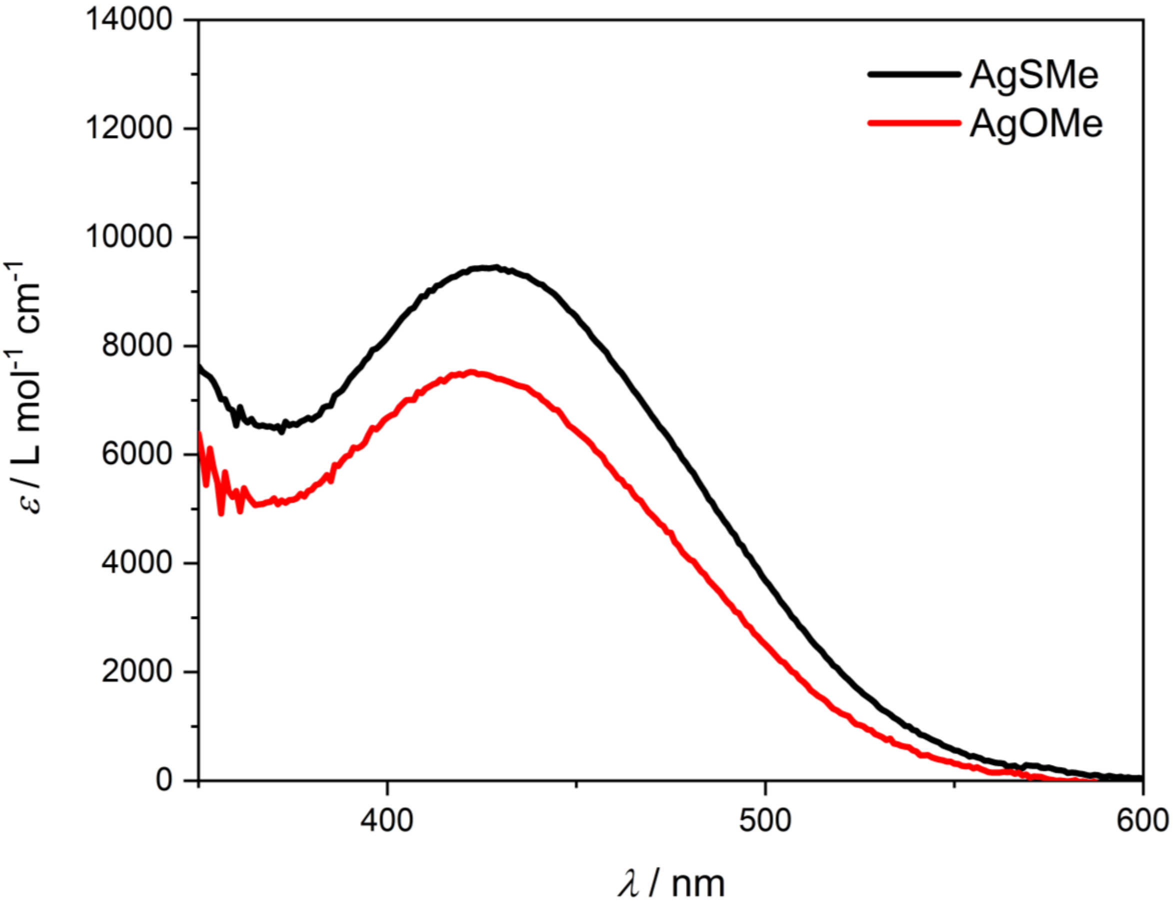

Figure S 67: Zoomed overlay of UV-Vis spectra from **Ag-OMe** and **Ag-SMe** in ACN.

[1] M. Gasperini, F. Ragaini, S. Cenini, *Organometallics* **2002**, *21*, 2950.
[2] V. Rosa, C. I. M. Santos, R. Welter, G. Aullón, C. Lodeiro, T. Avilés, *Inorganic chemistry* **2010**, *49*, 8699.
[3] L. Hoof, N. Thissen, K. Pellumbi, K. junge Puring, D. Siegmund, A. K. Mechler, U.-P. Apfel, *Cell Reports Physical Science* **2022**, *3*, 100825.
[4] B. Endrődi, E. Kecsenovity, A. Samu, T. Halmágyi, S. Rojas-Carbonell, L. Wang, Y. Yan, C. Janáky, *Energy Environ. Sci.* **2020**, *13*, 4098.
[5] S. Ma, Y. Lan, G. M. J. Perez, S. Moniri, P. J. A. Kenis, *ChemSusChem* **2014**, *7*, 866.
[6] X. Wu, J. W. Sun, P. F. Liu, J. Y. Zhao, Y. Liu, L. Guo, S. Dai, H. G. Yang, H. Zhao, *Adv Funct Materials* **2022**, *32*.
[7] S. Zhou, L.-J. Zhang, L. Zhu, C.-H. Tung, L.-Z. Wu, *Advanced materials (Deerfield Beach, Fla.)* **2023**, *35*, e2300923.
[8] K. Seteiz, J. N. Häberlein, P. A. Heizmann, J. Disch, S. Vierrath, *RSC advances* **2023**, *13*, 18916.
[9] K. Seteiz, J. N. Häberlein, P. A. Heizmann, L. Bohn, S. Vierrath, J. Disch, *ACS Appl. Eng. Mater.* **2024**, *2*, 1654.
